# Colonization dynamics of extended-spectrum beta-lactamase-producing Enterobacterales in the gut of Malawian adults

**DOI:** 10.1038/s41564-022-01216-7

**Published:** 2022-09-05

**Authors:** Joseph M. Lewis, Madalitso Mphasa, Rachel Banda, Mathew A. Beale, Eva Heinz, Jane Mallewa, Christopher Jewell, Brian Faragher, Nicholas R. Thomson, Nicholas A. Feasey

**Affiliations:** 1 Malawi-Liverpool Wellcome Clinical Research Programme, Blantyre, Malawi; 2 Liverpool School of Tropical Medicine, Liverpool, UK; 3 University of Liverpool, Liverpool, UK; 4 Wellcome Sanger Institute, Hinxton, UK; 5 Kamuzu University of Health Sciences, Blantyre, Malawi; 6 University of Lancaster, Lancaster, UK; 7 London School of Hygiene and Tropical Medicine, London, UK

**Keywords:** Antimicrobial resistance, Bacterial genetics, Bacteriology, Clinical microbiology

## Abstract

Drug-resistant bacteria of the order Enterobacterales which produce extended-spectrum beta-lactamase enzymes (ESBL-Enterobacterales, ESBL-E) are global priority pathogens. Antimicrobial stewardship interventions proposed to curb their spread include shorter courses of antimicrobials to reduce selection pressure but individual-level acquisition and selection dynamics are poorly understood. We sampled stool of 425 adults (aged 16–76 years) in Blantyre, Malawi, over 6 months and used multistate modelling and whole-genome sequencing to understand colonization dynamics of ESBL-E. Models suggest a prolonged effect of antimicrobials such that truncating an antimicrobial course at 2 days has a limited effect in reducing colonization. Genomic analysis shows largely indistinguishable diversity of healthcare-associated and community-acquired isolates, hence some apparent acquisition of ESBL-E during hospitalization may instead represent selection from a patient’s microbiota by antimicrobial exposure. Our approach could help guide stewardship protocols; interventions that aim to review and truncate courses of unneeded antimicrobials may be of limited use in preventing ESBL-E colonization.

## Abstract (web summary)

Longitudinal sampling, modelling and genomic analyses of stool samples from Malawian adults reveal how antimicrobial exposure and hospitalization promote ESBL-Enterobacterales colonization.

## Main

Antimicrobials are one of the most successful therapies available to modern medicine but the spread of antimicrobial resistance (AMR) is a threat to their effective use. Considerable global effort is being directed at antimicrobial stewardship programmes which the World Health Organization considers a key tool in reducing AMR^1^. Antimicrobial stewardship at the individual level often emphasizes rationalization of antimicrobials through narrowing their spectrum of action as soon as possible after commencement of broad empiric antimicrobial therapy in severely unwell individuals. The time frame (for example, 48 h) for this is typically pragmatically selected to match likely availability of diagnostic test results. Rationalization of therapy is partly based on the assumption that it will reduce emergence of AMR but the mechanism by which antimicrobial exposure acts at the individual level to promote colonization and/or infection with resistant pathogens, and the dynamics of colonization and decolonization, are not well understood^2–5^. Improved understanding of the dynamics of individual-level AMR-acquisition under antimicrobial pressure can therefore inform the design of stewardship protocols.

One setting in which antimicrobial stewardship is a considerable challenge is in the treatment of severe febrile illness in the low- and middle-income countries of sub-Saharan Africa (sSA). In Blantyre, Malawi, for example, as in much of sSA, limited availability of diagnostics results in prolonged courses of broad-spectrum antimicrobials—mainly ceftriaxone, a third-generation cephalosporin (3GC) antibiotic^6^—for severe febrile illness. Ceftriaxone has been extensively used since its introduction to the Malawian national formulary in 2005^7^ but this has been associated with an increase in 3GC resistance^8^, particularly in bacteria of the order Enterobacterales. This is mainly mediated by extended-spectrum beta-lactamase (ESBL) enzymes^8–10^. ESBL-producing Enterobacterales (henceforth ESBL-E) are an increasing public health challenge throughout much of sSA^11,12^ and often have few or no locally available treatment options; in Blantyre, 91% of invasive *Klebsiella pneumoniae* are now 3GC resistant^8^ and strategies to reduce ESBL-E infections are needed.

Gut mucosal colonization with ESBL-E is thought to precede invasive infection, is common across sSA and has often been found to be associated with prior hospitalization and/or antimicrobial exposure^12,13^. An improved mechanistic understanding of colonization dynamics following these exposures therefore has the potential to inform evidence-based interventions to reduce colonization and hence opportunity for transmission. Here, we present the results from a clinical study of longitudinal ESBL-E carriage in Blantyre, Malawi, sampling adults as they pass through the hospital and are exposed to antimicrobials. We use multistate modelling^14^ and whole-genome sequencing as a high-resolution bacterial typing tool to describe and understand the dynamics of ESBL-E colonization.

## Results

### Antimicrobial exposure drives increase in ESBL-E prevalence

**Fig. 1:**
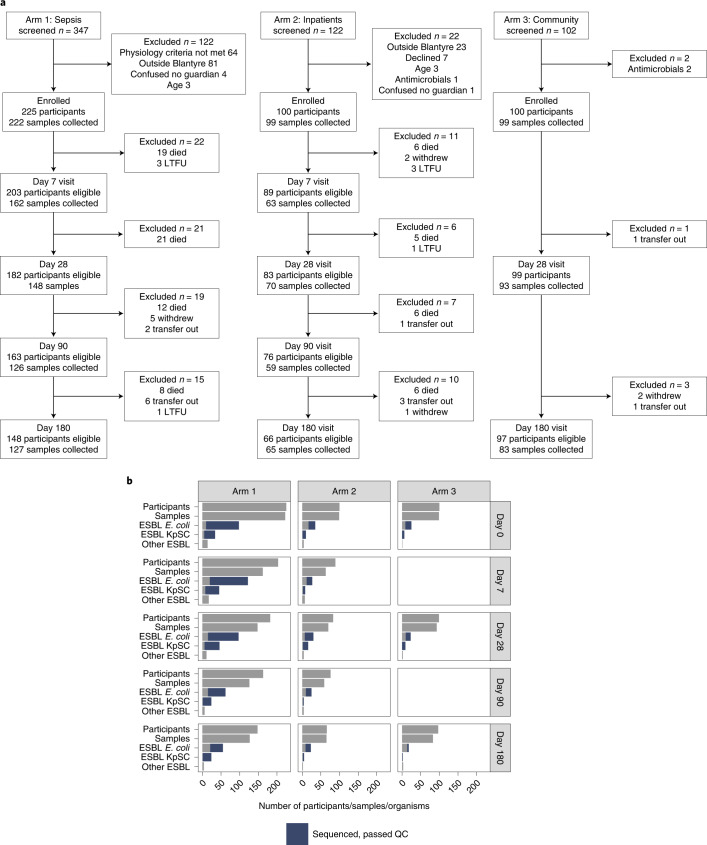
Study overview. **a**,**b**, Flow of patients through study (**a**) and number of samples collected at each time point (**b**) showing number of samples in which ESBL *E. coli*, *K. pneumoniae* sequence complex or other species were identified and how many *E. coli* or *K. pneumoniae* sequence complex isolates were sequenced and passed QC (blue colouring of bar). QC, quality control; LTFU, loss to follow up.

**Extended Data Fig. 1:**
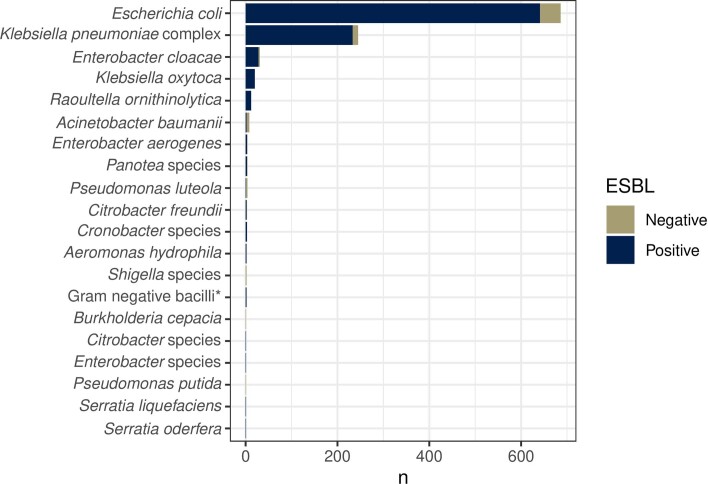
Species of bacteria isolated from stool. Samples were labelled “Gram negative bacilli” if they could not be speciated using the API system.

**Extended Data Fig. 2:**
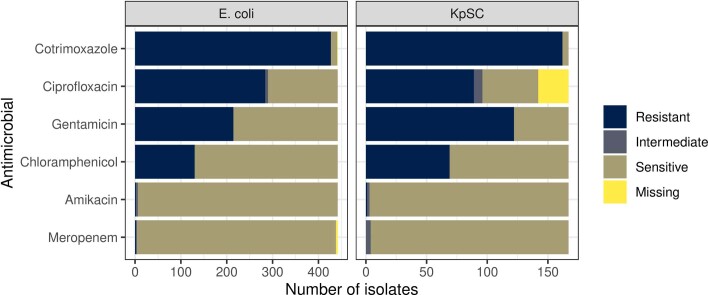
Results of antimicrobial sensitivity testing (AST). AST of cultured *E. coli* and *K. pneumoniae* sequence complex (KpSC) isolates using the disc diffusion method. A subset of isolates (442/473 E. coli and 167/203 KpSC) underwent AST.

Between 19 February 2017 and 2 October 2018, we recruited 425 adults: (1) 225 patients with sepsis and antimicrobial exposure, admitted to Queen Elizabeth Central Hospital (QECH), Blantyre; (2) 100 antimicrobial-unexposed inpatients and (3) 100 antimicrobial-unexposed community participants (Table 1). There were 1,631 study visits, with successful stool or rectal swab collection at 1,417/1,631 (87%) visits; missing samples were equally distributed across all study arms and visits (Fig. 1 and Supplementary Fig. 1). At least one ESBL-E species was cultured in 723/1,417 (51%) of samples. A total of 1,032 organisms were isolated, most commonly *Escherichia coli* (*n* = 686) and *K. pneumoniae* species complex (KpSC, *n* = 245; Fig. 1, Supplementary Table 1 and Extended Data Fig. 1). Phenotypic resistance to other antimicrobial classes (determined for 442 *E. coli* and 167 KpSC) was common (Extended Data Fig. 2).

**Table 1:** Baseline characteristics of included participants

Variable	Sepsis, receiving antibiotics (*n* = 225)	Inpatient, not receiving antibiotics (*n* = 100)	Community, not receiving antibiotics (*n* = 100)	*P*	Total (*n* = 425)
**Demographics**					
Age (yr)	35.9 (27.8–43.5)	40.4 (29.1–48.3)	32.5 (24.0–38.4)	<0.001	35.6 (26.9–43.9)
Male	114/225 (51%)	51/100 (51%)	40/100 (40%)	0.163	205/425 (48%)
**HIV status**					
HIV-positive	143/225 (64%)	12/100 (12%)	18/100 (18%)	<0.001	173/425 (41%)
HIV-negative	70/225 (31%)	77/100 (77%)	22/100 (22%)		169/425 (40%)
HIV unknown	12/225 (5%)	11/100 (11%)	60/100 (60%)		83/425 (20%)
**ART status**^a^					
Current CPT	98/141 (70%)	5/12 (42%)	7/18 (39%)	0.013	110/171 (64%)
Current ART	117/143 (82%)	9/12 (75%)	18/18 (100%)	0.082	144/173 (83%)
Months on ART	28.7 (3.7–72.6)	35.1 (2.9–79.8)	31.5 (13.0–79.9)	0.698	29.5 (3.8-72.8)
**Healthcare exposure**					
Antibiotics within 28 d^b^	60/225 (27%)	0/100 (0%)	0/100 (0%)	<0.001	60/425 (14%)
Hospitalized within 28 d	18/225 (8%)	1/100 (1%)	0/100 (0%)	<0.001	19/425 (4%)
Current TB treatment	10/225 (4%)	0/100 (0%)	4/100 (4%)	0.083	14/425 (3%)
**Household**					
Number of adults	2.0 (2.0–3.0)	3.0 (2.0–4.0)	2.0 (2.0–4.0)	0.907	3.0 (2.0–4.0)
Number of children	2.0 (1.0–3.0)	2.0 (1.0–3.0)	2.0 (1.0–3.0)	0.395	2.0 (1.0–3.0)
Keep animals	71/225 (32%)	43/100 (43%)	15/100 (15%)	<0.001	129/425 (30%)
-Poultry	46/71 (65%)	34/43 (79%)	10/15 (67%)		90/129 (70%)
-Dogs	18/71 (25%)	11/43 (26%)	9/15 (60%)		38/129 (29%)
-Goats	12/71 (17%)	7/43 (16%)	1/15 (7%)		20/129 (16%)
-Other	3/71 (4%)	6/43 (14%)	0/15 (0%)		9/129 (7%)
Electricity in house	119/225 (53%)	41/100 (41%)	58/100 (58%)	0.041	218/425 (51%)
Flush toilet^c^	14/225 (6%)	5/100 (5%)	1/100 (1%)	0.110	20/425 (5%)
Protected water source^d^	216/225 (96%)	92/100 (92%)	98/100 (98%)	0.124	406/425 (96%)
Treat drinking water with chlorine	19/225 (8%)	5/100 (5%)	0/100 (0%)	0.004	24/425 (6%)

*P* values are from two-sided Fisher’s exact test or Kruskal–Wallis for categorical or continuous variables, respectively.

ART, antiretroviral therapy; CPT, co-trimoxazole preventative therapy; TB, tuberculosis. Numeric variables are presented as median (IQR) and categorical variables as proportions. *P* values are from Fisher’s exact test or Kruskal–Wallis tests (categorical or continuous variables, respectively) across the three groups; *P* value for HIV status compares distribution of HIV status across the three groups. In some cases, denominator may be less than the total number of participants due to missing data.

^a^Denominator for ART status is HIV reactive participants only. ^b^Excluding TB treatment and CPT. ^c^Flush toilet versus latrine (pit or hanging) or no toilet. ^d^Protected water source includes borehole, water piped into or outside dwelling or public standpipe; unprotected sources include surface water or unprotected springs.

**Fig. 2:**
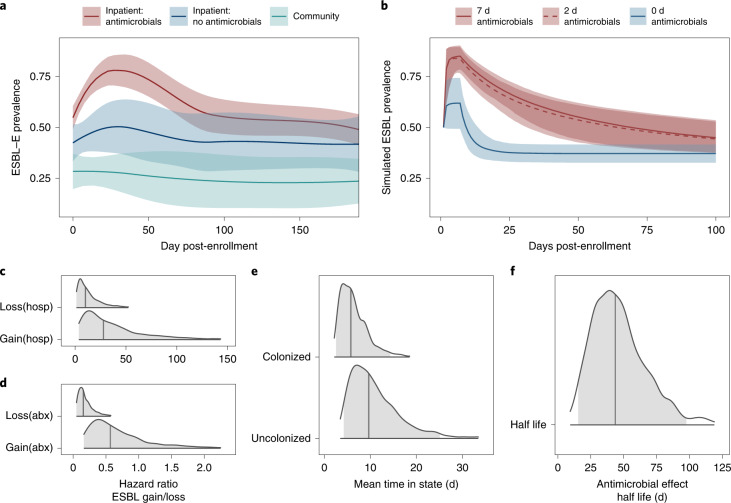
Prevalence and determinants of longitudinal ESBL-E carriage. **a**, ESBL prevalence stratified by the three study groups; inpatients exposed to antimicrobials (red), inpatients without antimicrobial exposure (blue), community members (green), showing sharp increase in prevalence following antimicrobial exposure. Prevalence is estimated using a LOESS non-parametric regression with 95% CI. Community members are censored on antimicrobial exposure or hospitalization and antimicrobial-unexposed inpatients on antimicrobial exposure. **b**, Simulated ESBL-E prevalence (with 95% CrI) using final fitted model for a hypothetical cohort of patients with initial ESBL-E colonization prevalence 50%, admitted to hospital for 7 d and exposed to 7, 2 or 0 d of antimicrobials, showing that there is little difference between 7 and 2 d**. c**–**f**, Posterior estimates of parameter values from final fitted model. Shaded grey areas shows 95% CrI and grey vertical line shows median parameter estimate. **c**,**d**, Hazard ratio of gain or loss of ESBL-E (expressed as natural logarithm) showing that antimicrobial exposure (abx) acts primarily to prolong carriage by reducing ESBL-E loss, whereas hospitalization (hosp) acts to increase both gain and loss, with a net effect to increase prevalence. **e**, Mean time in colonized/uncolonized states with all covariate values set to 0 (that is, no hospitalization or antimicrobial exposure). **f**, Half-life of effect of antimicrobial exposure, showing that antimicrobial exposure acts with a prolonged effect to prolong colonization.

Baseline prevalence of ESBL-E colonization was 178/420 42% (95% confidence interval (CI) 38–47%, Fig. 2a and Supplementary Table 1). In multivariable modelling (Supplementary Table 2), co-trimoxazole preventative therapy exposure (CPT, administered lifelong for people living with HIV as per WHO guidelines) was associated with ESBL-E colonization (adjusted odds ratio (aOR) of colonization 2.34, 95% CI 1.00–5.66) as was use of unprotected water sources (aOR 2.96, 95% CI 1.07–8.75), rainy season (aOR 2.21, 95% CI 1.07–8.75), number of adults in the household (aOR 1.20, 95% CI 1.03–1.40) and recent hospitalization (aOR 6.64, 95% CI 1.98–30.75)).

**Extended Data Fig. 3:**
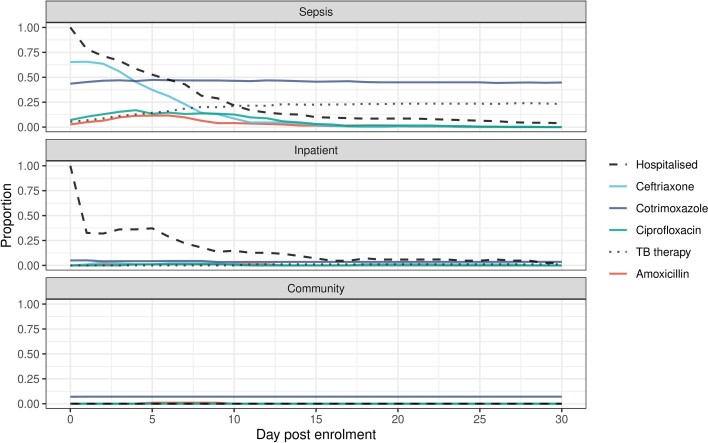
Participant antimicrobial exposure and hospitalization stratified by study arm. Y axis shows proportion of participants with a given exposure on each day post enrolment.

Following enrolment, there was rapid increase in ESBL-E colonization prevalence in antimicrobial-exposed inpatients (109/222 (49%) day 0 to 127/162 (78%) day 7) compared to the antimicrobial-unexposed inpatients (41/99 (41%) day 0 to 32/62 (51%) day 7; Fig. 2a and Supplementary Fig. 2). Ceftriaxone was the most commonly received antimicrobial (183/225, 80%) followed by co-trimoxazole (110/225, 49%), ciprofloxacin (61/225 27%) and antitubercular chemotherapy (52/225, 23%) but person-days of co-trimoxazole exposure was higher because of chronic CPT administration (Extended Data Fig. 3 and Supplementary Table 3). Median (interquartile range, IQR) length of hospital stay was longer in the antimicrobial-exposed (5 (IQR 2–10) ﻿days) compared to the antimicrobial-unexposed (2 (IQR 2–7) days) inpatient groups.

**Extended Data Fig. 4:**
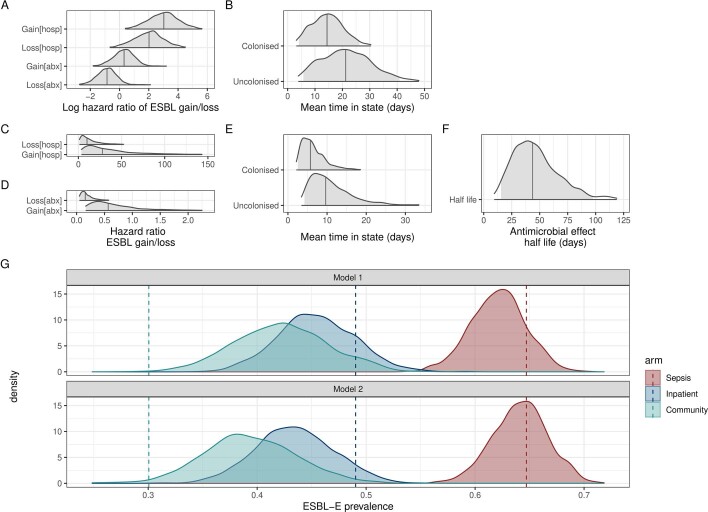
Comparing ESBL-E carriage models. Comparing a model of ESBL-E carriage that include a stepwise-constant effect of hospitalization and antimicrobial exposure (where the effect of covariates ceases when exposure ceases in Model 1) to a model that allows the effect of antimicrobial exposure to persist when exposure finishes, modelled as an exponential decay in Model 2. A-B: Parameter estimates from Model 1 expressed as natural logarithm of hazard ratio of gain or loss of ESBL-E for antimicrobial exposure [abx] and hospitalization [hosp] (A) and mean time in the colonized or uncolonised state (B) with covariates set to 0 (that is no antimicrobials, not hospitalized). C-F: Parameter estimates from Model 2, with the same interpretation and the addition of the half-life (in days) of the decaying effect of antimicrobial exposure (F). In A–F, vertical line represents posterior median, and grey shaded area 95% CrI. G: Posterior parameter checks of two models showing actual prevalence of ESBL-E carriage stratified by study arm (dashed lines) with kernel density plots of predicted prevalence from fitted models, obtained by using all posterior parameter estimates (n = 2000, discarding warmup iterations) to predict probability of ESBL-E from the actual data, and sampling from a binomial distribution using this probability. Model 1 underfits the antimicrobial-exposed arm of the study, which is improved by the addition of the prolonged effect of antimicrobials.

We used continuous-time multistate Markov models to understand determinants of ESBL-E carriage and to account for differences in exposures across the arms of the study. In this model, each patient is ‘colonized’ or ‘non-colonized’, with the transition rate governed by a linear function of time-varying covariates (hospitalization and antimicrobial exposure). When comparing a stepwise-constant covariate model (where the effect of hospitalization and antimicrobial exposure cease immediately as exposure ceases) to a model which included a prolonged effect of antimicrobial exposure, modelled as an exponential decay that continues to exert an effect when exposure ceases, the latter was a better fit to the data as assessed by leave-one-out cross-validation (estimated expected pointwise log predictive value (ELPD) difference 10.5 (standard error 4.2) in favour of the exponential decay model) and posterior predictive checks (Extended Data Fig. 4).

**Extended Data Fig. 5:**
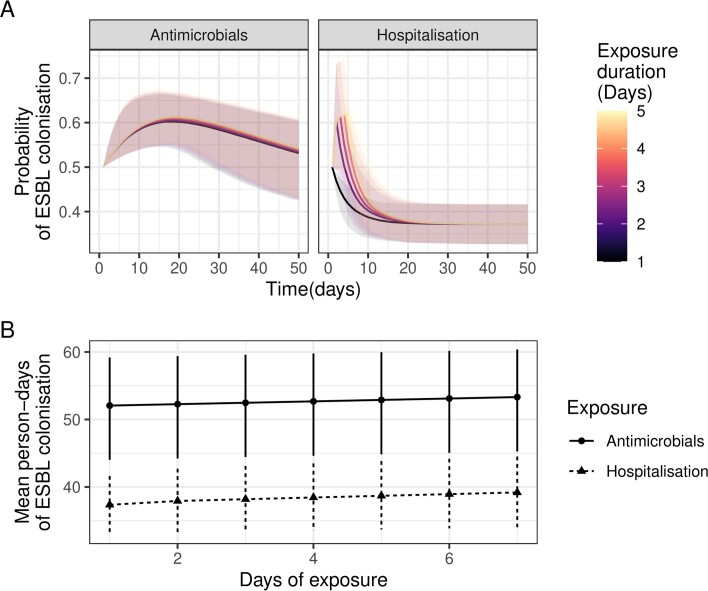
Visualizing relative effects of hospitalization and antimicrobial exposure. A: Predicted probability from final fitted model of ESBL colonization assuming 0.5 probability of colonization at time t = 0 and between 1–5 days of antimicrobial exposure (left) or hospitalization (right). B: Estimated mean person-days of colonization for the exposures in A out to 100 days (that is the area under the curves in A top 100 days). In all panels confidence intervals are 95% credible intervals; the full model posterior (n = 2000 replicates) was used to estimate the quantities of interest and confidence intervals constructed from quantiles of estimates.

In this model, hospitalization increased both ESBL-E gain and loss parameters resulting in a modest increase in overall carriage prevalence, whereas antibacterial therapy largely acted to prolong ESBL-E carriage by reducing loss and acted with a prolonged effect with half-life 43.7 (95% credible interval (CrI) 15.4–97.7) days (Fig. 2c,e and Supplementary Table 4). Posterior plots of pairs of parameters revealed some non-identifiability between the gain and loss parameters, manifesting as correlation (Supplementary Fig. 3). Overall, in terms of estimated person-days of colonization, antimicrobial exposure had a greater effect than hospitalization (Extended Data Fig. 5). Posterior predictive simulations from the final fitted model (Fig. 2b) considering an hypothetical 7-day hospital admission with 7, 2 or 0 days of antimicrobial therapy suggest that antimicrobial therapy and hospitalization act together to produce the observed rapid increase in ESBL-E but that there is very little difference in ESBL-E prevalence carriage from truncating 7 days of antimicrobial therapy to 2 days.

**Extended Data Fig. 6:**
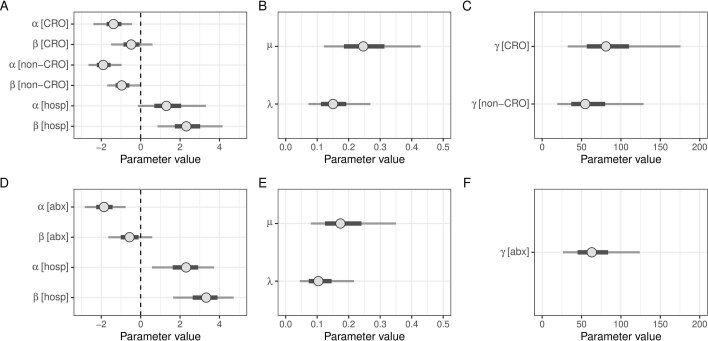
Parameter estimates from models considering ceftriaxone and non-ceftriaxone antimicrobials. (A-C) show parameter estimates from this model; (D-F) show original model. In each case, point shows posterior median and bar and whiskers show undertainty in parameter estimate by 50% and 95% credible interval, respectively, constructed from quantiles of all the posterior estimates (n = 2000 replicates). Parameters are: a, loss parameters, b, gain parameters and g, decay parameter of effect of antimicrobials. [CRO] indicates that the parameter refers to ceftriaxone, [non-CRO] that the parameter refers to non-ceftriaxone antimicrobials, and [hosp] to hospitalization.

In sensitivity analysis, we refit the final model but disaggregated antimicrobial exposure into ceftriaxone and non-ceftriaxone exposure. The effect of ceftriaxone was similar to non-ceftriaxone antimicrobials (Extended Data Fig. 6) suggesting coselection of ESBL-E carriage by exposure to non-beta-lactam antimicrobials.

### Within-host ESBL persistence mechanism not horizontal gene transfer

**Extended Data Fig. 7:**
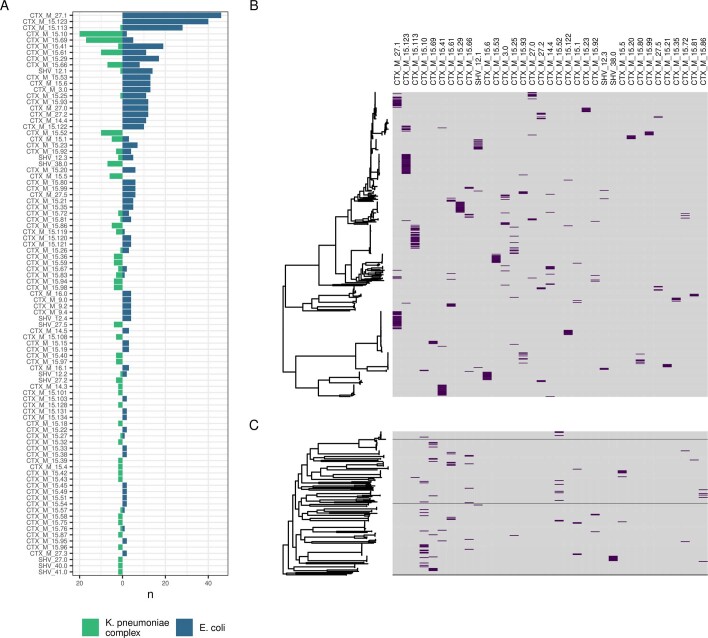
Distribution of contig-clusters between and within genera. (A) shows distribution of contig-clusters by genus. (B-C) show contig-cluster presence (purple)-absence (grey) mapped back to core-gene maximum-likelihood phylogeny for *E. coli* (B) and *K. pneumoniae* subsp. *pneumoniae* (C).

Next we used short-read whole-genome sequencing to track bacteria and ESBL genes within study participants. Following quality control, 473 *E. coli* and 203 KpSC genomes were included in the analysis with a median (IQR) 3 (2–4) *E. coli* isolates per participant from 230 participants and 2 (1–2) *KpSC* isolates per participant from 142 participants. Most (*n* = 190) KpSC isolates were *K. pneumoniae* subsp. *pneumoniae*. An analysis of population structure, core-gene phylogeny and AMR and plasmid gene content of these isolates has previously been made^15,16^ and AMR gene and plasmid incompatibility group (Inc-types) content is summarized in Supplementary Figs. 4–6. To track bacteria within-participant we mapped reads to reference genomes and defined high-level sequence clusters using popPUNK-^17^ and single nucleotide polymorphism (SNP)-clusters as isolates with whole-genome SNP distance ≤5. PopPUNK grouped *E. coli* into 87 clusters representing 58 sequence types (STs) and KpSC into 91 clusters representing 75 STs, 55 of these *K. pneumoniae* subsp. *pneumoniae* (Supplementary Figs. 7 and 8). These clusters (henceforth, popPUNK-clusters) were largely concordant with the core-gene phylogenies (Supplementary Fig. 9). To track ESBL genes and their genomic environment (because full plasmids usually fail to assemble into a single molecule with de novo assembly of short reads due to repeat-regions) we clustered de novo assembled contigs containing 3GC-resistance genes using the cd-hit^18^ algorithm, including those from both KpSC and *E. coli*. A total of 714 3GC-resistance gene-containing contigs were identified in 672/676 samples; 18 different genes formed 195 clusters (henceforth, contig-clusters) of median size 1 (range 1–42; Supplementary Fig. 10). They were genus- and lineage-associated (Extended Data Fig. 7), although 21/195 (11%) of contig-clusters contained both *E. coli* and KpSC genomes. In sensitivity analysis, cluster membership was stable to increasing the sequence identity and length cut-off of the cd-hit algorithm (Supplementary Fig. 11), although with some fragmentation of clusters apparent at sequence identity of 1.0. The nucleotide diversity and insertion sequence, AMR gene and plasmid replicon content of the ten most common contig-clusters (present in 248/714 (35%) of samples) is shown in Supplementary Figs. 12–21. Generally, shorter assembled contigs terminated in insertion sequences (consistent with compound transposons), often IS26, and showed low nucleotide diversity to the portion of the cluster representative to which they were matched. Where nucleotide diversity was present, it was often flanking transposable elements, which could be consistent with transfer/rearrangement events. In some cases, the ESBL gene was assembled onto a contig with a plasmid replicon but this was not the norm.

**Fig. 3:**
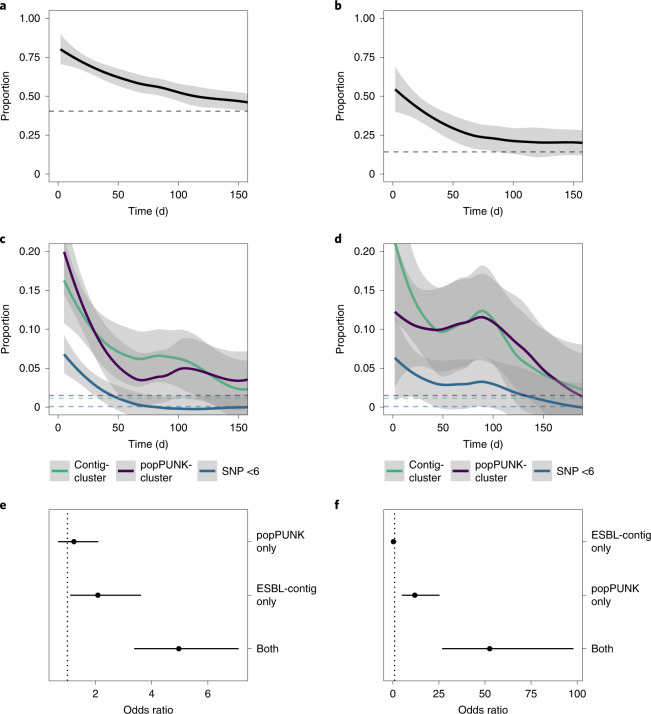
Within-participant dynamics of ESBL-E colonization. **a**,**b**, Proportion of participants who, at time 0 have detectable ESBL-producing *E. coli* (**a**) or KpSC (**b**) who remain colonized as a function of time. Horizontal dotted lines in each panel show the baseline proportion for the dataset of between-participant samples that contain the same genus. **c**,**d**, Proportion of participants with detectable ESBL-producing *E. coli* (**c**) or KpSC (**d**) at time 0, who remain colonized with the same contig-cluster, popPUNK-cluster or an isolate of SNP distance ≤5 as a function of time with dotted lines showing the baseline (between-participant) proportion, as above. **e**,**f**, Odds ratio from logistic regression with 95% CIs of within-participant sample pairs containing the same popPUNK-cluster alone, contig-cluster alone or both, compared to between-participant pairs for *E. coli* (**e**) and KpSC (**f**) showing that the element that is most likely to be conserved is the popPUNK-cluster contig-cluster combination. This analysis (**e**,**f**) uses the sample pair as the unit of analysis so each sample may be included more than once.

For participants colonized with *E. coli* or KpSC at a time *t* = 0, the probability of remaining colonized returned to a baseline by 100–150 d (Fig. 3a,b) but the probability of remaining colonized with the same contig-cluster or popPUNK-cluster was lower and the probability of remaining colonized with an organism differing by five or fewer SNPs was lower still (Fig. 3c,d), suggesting considerable within-participant strain diversity. Nevertheless, a temporal signal was present: two samples closer together in time were more likely to contain the same popPUNK-, contig- and SNP-cluster, enabling us to seek hospital-associated transmission events. Sensitivity analysis varying the definition of SNP-cluster from 0 to 20 SNPs did not alter these conclusions (Supplementary Fig. 22). Comparing within-patient sample pairs to between-patient sample pairs, the popPUNK-cluster contig-cluster combination was conserved more than either popPUNK-cluster or contig-cluster alone (Fig. 3e,f), consistent with the suggestion that within-participant persistence of ESBL, where it occurs, is caused by persistence of ESBL-containing bacteria rather than horizontal gene transfer and persistence of ESBL genes.

### Hospital-linked lineages/transmission clusters are unusual

**Fig. 4:**
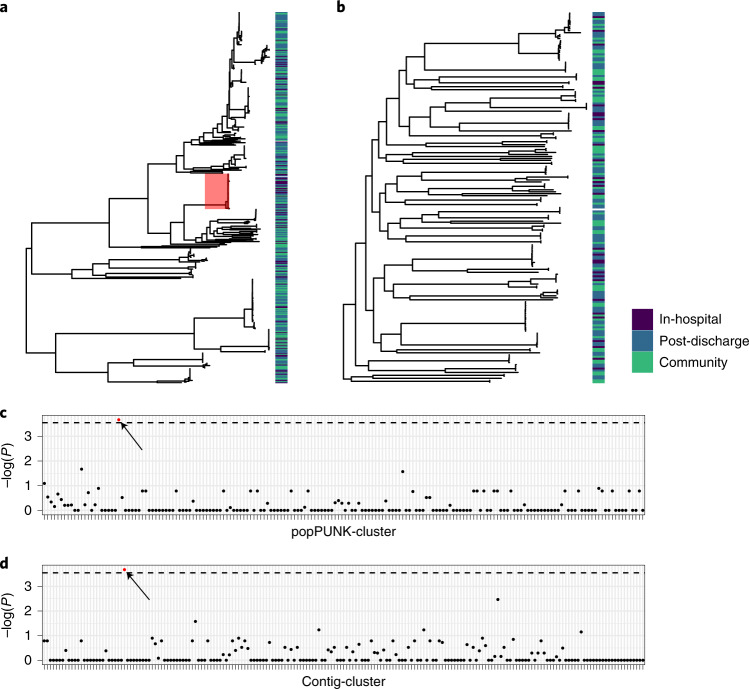
Hospital association of popPUNK-clusters and contig-clusters. **a**,**b**, Maximum-likelihood core-gene phylogenetic tree for *E. coli* (**a**) and *K. pneumoniae* subsp. *pneumoniae* (**b**) showing in-hospital (dark blue), post-discharge (light blue) and community (green) isolates, where post-discharge is defined as up to 120 d post-hospital discharge. Hospital-associated samples are distributed across the tree but only the popPUNK-cluster highlighted in red shows an association with in-hospital isolation. **c**,**d**, Manhattan plots showing *P* value of two-sided Fisher’s exact test for association of popPUNK-cluster (**c**) and contig-cluster (**d**) with in-hospital isolation. Dotted line shows Bonferroni-corrected value corresponding to *P* = 0.05. Only one popPUNK-cluster is significantly associated with in-hospital isolation (highlighted in red on the plot (**c**) and core-gene tree (**a**)) at this level. Similarly, one contig-cluster is associated with in-hospital isolation, highlighted in red; this is the contig-cluster associated with the hospital-associated lineage.

Next, we examined any hospital association of popPUNK-clusters. In-hospital and post-discharge isolates were distributed throughout the core-gene phylogenies and only one popPUNK-cluster contained more hospital isolates than would be expected by chance following correction for multiple comparisons (Fig. 4a,c). This corresponded to *E. coli* ST410. Similarly, one contig-cluster was associated with in-hospital isolation (Fig. 4d); this *bla*_CTX-M-15_ containing contig-cluster was primarily associated with *E. coli* ST410 (CTX_M_15.113 in Extended Data Fig. 7b). Sensitivity analysis aggregating in-hospital and post-discharge isolates to a ‘healthcare-associated’ category did not identify any distinctly healthcare-associated popPUNK- or contig-clusters (Supplementary Fig. 23).

**Fig. 5:**
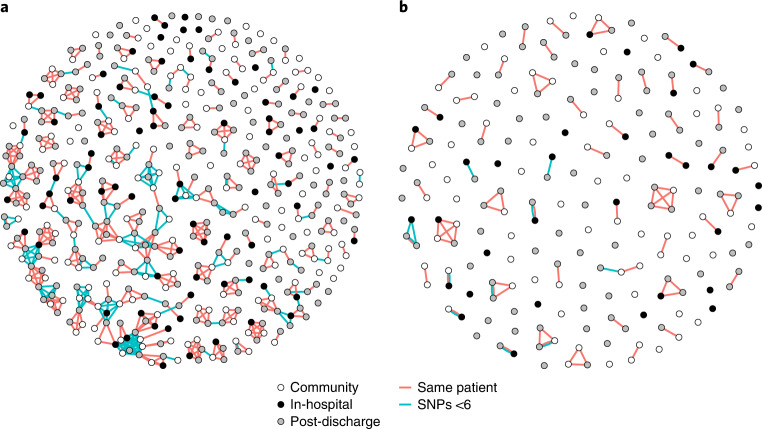
Visualising networks of SNP-clusters. **a**,**b**, Network plot of SNP-clusters (putative transmission clusters) for *E. coli* (**a**) and *K. pneumoniae* species complex (**b**) showing that putative transmission clusters are not exclusively hospital associated. Points are samples, coloured by place of isolation (in-hospital (black), community (white) or up to 120 d post-discharge (grey)). Red lines link samples that are within a single participant. Blue lines link samples that are differ by five or fewer SNPs. The plot shows that most samples are not members of an SNP-cluster; that most SNP-clusters encompass samples from different (rather than multiple samples from the same) participants; and that SNP-clusters are not exclusively hospital-associated, that is they contain in-hospital, community and post-discharge samples.

As hospital-associated popPUNK-clusters were infrequent, we investigated putative hospital-related transmission SNP-clusters which could represent transmission clusters. We found that 151/473 (32%) *E. coli* and 21/203 (10%) KpSC were members of an SNP-cluster and hence represent possible transmission events (Fig. 5). The clusters were generally small (median size 2 (IQR 2–5) for *E. coli* and 2 (IQR 2–3) for KpSC) and, in *E. coli*, mainly contained samples from different participants rather than the same participant: only 6% (10/175) of pairwise comparisons of within-SNP-cluster *E. coli* samples were from the same participant. Fewer KpSC formed an SNP-cluster but more were from the same participant (58% (7/12)) rather than between participants. Most clusters (149/192 (78%) *E. coli* and 31/57 (54%) KpSC) contained two or more healthcare-associated isolates, which might represent transmission events. However, the proportion of samples that were members of an SNP-cluster were similar between healthcare-associated isolates and community isolates. For *E. coli* 54/171 (32%) of community isolates versus 96/300 (32%) of healthcare-associated isolates were members of an SNP-cluster (*P* = 1.00, Fisher’s exact test). For KpSC 4/74 (5%) of community isolates versus 17/128 (13%) of healthcare-associated isolates were members of an SNP-cluster (*P* = 0.15). This is not consistent with widespread hospital-associated transmission above the level of community transmission. Sensitivity analysis varying the SNP threshold from 0 to 10 did not materially alter the conclusions (Supplementary Figs. 24–26).

## Discussion

Combining longitudinal sampling, multistate modelling and whole-genome sequencing, we describe the dynamics of ESBL-E colonization in Malawian adults. These findings advance our understanding of the effects of antimicrobial exposure on AMR-acquisition, with potentially notable implications for the directions of future research into the design of both antimicrobial stewardship and infection prevention and control interventions.

First, baseline sampling provides insight into drivers of ESBL-E colonization in Blantyre. ESBL-E colonization is very common and identification of community risk factors for baseline colonization, suggests considerable community transmission consistent with other studies across sSA^12^ and elsewhere^19^. ESBL-E colonization was associated with unprotected water use for drinking and higher prevalence in rainy season which suggests inadequate access to water, sanitation and hygiene (WASH) infrastructure and/or WASH behavioural practices may be contributing. Associations of colonization with household crowding suggests within-household transmission.

Second, Markov models fitted to longitudinal sampling data allow insight into the dynamics of ESBL-E colonization. We demonstrate a rapid increase in ESBL-E colonization following hospital admission and antimicrobial exposure. Modelling suggests that both hospitalization and antimicrobial exposure may act to drive this increase, although antimicrobial exposure has a greater effect by exerting an effect long after antimicrobial exposure finishes, with a half-life of 43.7 (95% CrI 15.4–97.7) days. Simulations suggest that, due to the sustained effect of antimicrobials, short courses of antimicrobials could exert a similar effect to that of prolonged courses in terms of ESBL-E carriage. This finding has clear implications for antimicrobial stewardship protocols, suggesting that truncating courses of antimicrobials may have limited effect on ESBL-E carriage compared to avoiding antimicrobial administration altogether. In addition, non-ceftriaxone antimicrobials exerted a similar effect on ESBL-E carriage as ceftriaxone; this, along with the high prevalence of resistance to other antimicrobial classes in these isolates, suggests that coselection for ESBL-E by non-beta-lactam agents is occurring. Hence, switching ceftriaxone to other classes of agent in treatment protocols may have a limited effect on ESBL-E carriage in this setting.

Previous ESBL-E longitudinal sampling and modelling studies examining the effect of antimicrobials on colonization have examined community and post-travel carriage in adults in the Netherlands^2,3,5^ and transmission of ESBL-E in neonatal units in the high-prevalence setting of Cambodia^20^. In the former studies, some association of ESBL-E carriage with antimicrobial exposure was found but antimicrobial exposure was not common; further, sampling was neither intensive nor linked to antimicrobial exposure to fully define the effects. In a Cambodian neonatal unit, antimicrobial therapy was robustly linked to an increased daily probability of acquiring *K. pneumoniae* colonization but long-term sampling was not available to define post-antimicrobial effects as we have done here. Further work to understand the dynamics of ESBL-E colonization and decolonization under antimicrobial pressure to guide stewardship efforts should be a priority in other settings to guide antimicrobial stewardship programmes.

More broadly, these findings highlight a need to define and measure clinically relevant individual-level AMR carriage as an endpoint in trials of antimicrobial treatment strategies. An expanding evidence base has demonstrated equivalence of clinical outcomes in a variety of clinical infection syndromes for shorter versus longer courses of antimicrobial therapy^21^ but a nonlinear relationship between antimicrobial exposure and colonization with AMR-bacteria (as we demonstrate here) may mean that 7 days compared to 14 days of antimicrobials (for example) has little benefit in reducing ESBL-E colonization. Defining clinically relevant AMR endpoints for trials and measuring them to understand their relationship with antimicrobial exposure is thus crucial for optimizing the way in which antimicrobials are used in clinical practice.

Third, using whole-genome sequencing as a high-resolution typing tool allowed us to explore the mechanism by which antimicrobials promote ESBL-E carriage. This is a key question: antimicrobials could either act to promote healthcare-associated transmission by reducing colonization resistance or select for low-abundance resistant bacteria that were already present in the microbiota but not detected by bacterial culture on admission. Hospitalization exerted an effect to increase ESBL-E colonization in the absence of antimicrobial exposure, suggesting that transmission within the hospital is occurring. However, we found limited support for hospital-associated lineages or hospital-associated transmission clusters above the level of transmission in the community. This suggests that either ESBL-E acquisition had occurred in the community and was enriched by antimicrobial exposure in hospital; that the diversity of isolates transmitted in the hospital was represented by the diversity of isolates in the community (a distinct possibility in our setting); or our sparse sampling strategy missed hospital-associated transmission events. Genomic epidemiology studies of ESBL-E colonization and infection clearly demonstrate that true healthcare-associated transmission of ESBL-E occurs^22–24^ but few studies have longitudinal sampling pre-, during- and post-antimicrobial exposure. Defining the contribution of antimicrobial selection versus new ESBL-E acquisition events following antimicrobial exposure will guide prevention efforts and should be a priority for future studies—as should understanding the way in which antimicrobials may act to reduce colonization resistance and aid transmission. Healthcare-associated transmission could be reduced by infection prevention and control procedures but antimicrobial selection pressure amplifying minority ESBL-E carried before hospital admission would need new strategies to protect the microbiota against selection for ESBL-E, such as antimicrobial binding compounds^25^ or oral beta-lactamases^26^.

We demonstrate considerable within-participant ESBL-E bacterial diversity (as defined by SNP-clusters and popPUNK-clusters) over time, even in participants who remain colonized with the same genus; a further key question is whether this temporal bacterial diversity with preserved ESBL-E colonization could represent horizontal gene transfer of ESBL genes between bacteria. Horizontal gene transfer could also explain an apparent lack of hospital-associated transmission clusters, if ESBL genes disseminated into diverse clones in the healthcare setting. We find that within-participant the popPUNK-cluster and contig-cluster combination was conserved more than either popPUNK-cluster or contig-cluster alone, consistent with the hypothesis that within-participant persistence of ESBL, where it occurs, is caused by persistence of ESBL-containing bacteria rather than horizontal gene transfer of ESBL genes to differing bacterial hosts. This does not support the suggestion of horizontal gene transfer as primary mechanism of ESBL temporal persistence within-participant on the timescale of the study.

There are limitations to our study. Most importantly, due to resource limitation, we took only one colony pick from each patient-time point sample for sequencing and so we may have missed intrahost ESBL-E diversity^27,28^ and hence underestimated the numbers of transmission clusters. We used short-read sequencing and clustered ESBL-containing contigs as a proxy for mobile genetic elements but our approach probably under-represents transfer/rearrangement events in the flanking contexts around ESBL genes. Hence, inferring that inclusion in a specific contig-cluster represents a single stable/consistent genetic construct should be done very cautiously. We used an arbitrary SNP threshold of five SNPs to define SNP-clusters, a strong assumption, which could misclassify isolates; this cut-off (empirically derived) has been used by public health bodies in England and Canada to define possible *E. coli* outbreaks^29,30^. We used a map-to-reference approach to identify core-genome SNPs that could have introduced bias due to the choice of reference. We have looked at high-level clustering with popPUNK and it may be that a high-resolution clustering approach using local, lineage-specific references would give the resolution to identify more hospital-associated transmission events. The models of AMR carriage assumed a 100% sensitivity and specificity of sampling, which may not be valid. We were not able to disaggregate the effect of different antimicrobial agents because of the sample size. We did not collect data on sibling or family connections between hospitalized and community participants which could explain apparent community links. For hospitalized patients, we did not sample the ward environment, carers, staff, food or toilets; and our sampling strategy was sparse. In the analysis of associations of ESBL-E colonization at enrolment, we relied on self-reported antimicrobial exposure and hospitalization and it is possible in our setting that people may take medication without knowing the exact nature of it and that this may differ from true antimicrobial exposure. Hospitalization records were not available to exclude the possibility that antimicrobials were received, which could explain some of the association between hospitalization and ESBL-E colonization at enrolment.

In conclusion, we describe the dynamics of ESBL-E colonization in Malawian adults as they are exposed to both antimicrobial therapy and hospitalization. Antimicrobial therapy and hospitalization act rapidly to promote ESBL-E colonization. Antimicrobial therapy exerts a prolonged effect which means that truncated courses of antimicrobials may have a similar effect to longer ones, which has implications for stewardship protocols. Short-read whole-genome sequencing did not identify widespread, distinct hospital-associated lineages or that putative hospital-associated transmission clusters were more common than community SNP-clusters. Future work should define dynamics of intrahost ESBL-E diversity under antimicrobial pressure, using longitudinal sampling, metagenomic sequencing methods to describe diversity and long-read sequencing to characterize mobile genetic elements. This will facilitate development of clinically relevant AMR endpoints for clinical trials and the development of a sound evidence base for stewardship protocols at the individual level—an evidence base that is currently lacking.

## Methods

### Study setting and design

The study took place in QECH, Blantyre, Malawi, a government tertiary referral hospital for the Southern Region of Malawi and the only hospital providing free healthcare to the ~800,000 residents^31^ of urban Blantyre. Nursing ratios at QECH are usually around two trained nurses to a 60-patient ward and basic nursing care is provided by family members; food is supplied to all patients on the ward by the hospital and each ward has one toilet which is shared by all patients. Malawi is a low-income country in southeast Africa, with an estimated adult human immunodeficiency virus (HIV) prevalence of 9% (UNAIDS, Malawi Country Profile https://www.unaids.org/en/regionscountries/countries/malawi) and a high tuberculosis incidence of 133/100,000 person-years^32^. Blantyre has a subtropical climate with a rainy season from November to April.

Adults (>15 years) with sepsis, defined by fever and organ dysfunction criteria, were recruited from the emergency department of QECH 7:00-17:00 Monday to Friday as part of a study of sepsis aetiology, as described elsewhere^33^. Two comparator cohorts of participants were recruited: age- and sex-matched adults from QECH emergency department who had a plan from their attending clinical team to admit to hospital but no plan for antimicrobial administration; and community members matched by age, sex and home location to recruited sepsis patients. Exclusion criteria were: for the last two groups, antimicrobial exposure within the past 4 weeks (except co-trimoxazole preventative therapy (CPT) and antituberculous chemotherapy); hospitalized participants who lacked capacity to give informed consent and had no guardian to give proxy consent; participants who spoke neither English nor Chichewa; and participants who lived >30 km from Blantyre city. Geographic matching on home location between community members and sepsis patients was achieved by random walk from the houses of sepsis participants with initial direction established by spinning a bottle on the floor. Written informed consent was obtained from all participants. An admission questionnaire was administered to all participants at enrolment and hospitalized patients were reviewed daily by a study team member until discharge to extract details of antimicrobial therapy from the clinical record. All clinical decisions were at the discretion of the attending clinical team. Further review by the study team occurred at days 7, 28, 90 and 180, except for community members in whom the day 7 and day 90 visits were omitted. If participants failed to come to their scheduled visits, then they were traced by telephone or, if that failed, by home visit. Hospitalized patients were not financially compensated for their time but all other participants were at a rate of 500 Malawian Kwacha (MWK) for home visits and 2,000 MWK for hospital visits. Data were captured using a combination of direct electronic data entry by study team members onto tablet devices (open data kit^34^, Get ODK Inc.) and paper forms (TeleForm, Opentext).

### Ethics statement

The study was approved by the research ethics committees of the Liverpool School of Tropical Medicine (16-062) and Malawi College of Medicine (P.11/16/2063).

### Microbiologic methods

At each study visit (enrolment, days 7, 28, 80 and 190 for hospitalized participants and enrolment, days 28 and 190 for community members) stool was collected in a sterile polypropylene pot; if a participant was not able to provide a stool sample, then a rectal swab was taken by a trained study team member and stored in Amies medium for transport. Stool and rectal swab samples were stored at 4 °C before being batch processed weekly: samples were plated directly onto commercially available ESBL selective chromogenic agar (CHROMagar ESBL, CHROMagar) and cultured aerobically overnight. Morphologically distinct white or blue colonies were speciated with the API 20E system (Biomerieux, France); pink colonies were identified as *E. coli*. ESBL production was confirmed with the combination disc method on iso-sensitest agar with discs of cefotaxime (30 μg) and ceftazidime (30 μg) with and without clavulanic acid (10 μg), with ESBL production confirmed if there was a difference of 5 mm or more between the clavulanic acid and non-clavulanic acid discs for either cephalosporin. For organisms likely to carry a chromosomal *bla*_*ampC*_ beta-lactamase gene and hence able to hydrolyse cefotaxime and ceftazidime (defined for our purposes as *Enterobacter* spp., *Citrobacter freundii, Morganella morganii, Providencia stuartii, Serratia* spp. and *Hafnia alvei*); cefipime (30 μg), an AmpC-stable cephalosporin was used with and without clavulanic acid (10 μg) and ESBL production confirmed if there was a difference of 5 mm or more between the clavulanic acid and non-clavulanic acid discs. For a subsample of isolates, antimicrobial sensitivity testing (AST) using the disc diffusion method on iso-sensitest agar following British Society for Antimicrobial Chemotherapy guidelines (https://bsac.org.uk/) was carried out for meropenem, amikacin, chloramphenicol, ciprofloxacin, co-trimoxazole and gentamicin. The first 442 *E. coli* and 167 *K. pneumoniae* species complex isolates cultured in the study (this number determined by resource and logistic considerations) underwent AST.

### DNA extraction, sequencing and bioinformatic analysis

Due to resource and logistic constraints, not all samples could be taken forward for sequencing: 503/686 *E. coli* and 217/233 *K. pnemoniae* species complex isolates were randomly selected from the collection for sequencing. One of each morphologically distinct *K. pneumoniae* species complex and *E. coli* colony, respectively, from each selected sample was taken forward for DNA extraction and whole-genome sequencing. DNA was extracted from overnight nutrient broth culture using the Qiagen DNA mini kit as per the manufacturer’s instructions. Extracted DNA was shipped to the Wellcome Sanger Institute to undergo whole-genome sequencing using Illumina HiSeq X10 to produce 150 base pair paired end reads. Quality control, de novo assembly and construction of core-gene phylogeny are described elsewhere^15,16^; in brief, species was confirmed with Kraken v.0.10.6 and Bracken v.1.0 (ref. ^36^) before de novo assembly with SPAdes v.3.14 (ref. ^37^), with the modifications described in ref. ^38^ and annotation with prokka v.1.5 (ref. ^39^) using a genus-specific database from RefSeq. The Roary v.1.17 pan-genome pipeline^40^ was used to identify core genes, considering genes contained in at least 99% isolates to be core. Samples with assembly failure (<4 megabases (Mb) assembled length) and samples with >10% contamination (as defined by CheckM v.1.1.3, ref. ^41^) were excluded from the analysis. A total of 203 KpSC and 473 *E. coli* genomes passed quality control and were included in the analysis. A core-gene multiple sequence alignment was generated using mafft v.7.205 (ref. ^42^), SNP-sites identified using SNP-sites v.2.4.1 (ref. ^43^) and the resultant SNP alignment (99,693 variable sites from a core-gene alignment of 1.39 Mb for *E. coli* and 378,596 variable sites from a 2.82 Mb core-gene alignment for *K. pneumoniae* complex) used to infer a maximum-likelihood phylogenetic tree using IQ-TREE v.1.6.3 (ref. ^44^) with the ModelFinder module, which selected the generalized time reversible model with FreeRate heterogeneity with five parameters for *E. coli* and eight parameters for *K. pneumoniae* complex. A total of 1,000 ultrafast bootstrap replicates were generated. Trees were visualized with ggtree v.2.2.4 (ref. ^45^).

AMR genes and plasmid replicons were identified using ARIBA v.2.14.6 (ref. ^46^) and the curated ARG-ANNOT database used by SRST2 (ref. ^47^) and PlasmidFinder^48^ databases, respectively, on the sequence reads. ARIBA was also used to identify multilocus ST using the 7-gene *Klebsiella*^49^ and 7-gene Achtman^50^ *E. coli* schemes hosted at pubMLST (https://pubmlst.org/). Since individual AMR genes from mobile genetic elements can exist in a variety of genomic contexts (for example, chromosomal, different plasmid backbones), we clustered ESBL-containing contigs from the de novo assemblies (identified with BLAST blastn v.2.7.1 (ref. ^51^) using the curated ARG-ANNOT database used by SRST2) to form contig-clusters using cd-hit-est v.4.8.1 (ref. ^18^) with 95% sequence identity and otherwise default settings. We varied cd-hit sequence identity from 95% to 100% and the length cut-off parameter from 0 to 0.8 (that is, for length cut-off *x*, cluster members must be at least a fraction *x* of the longest cluster member) in sensitivity analysis. To understand the genomic environment of the ESBL gene in these contig-clusters we identified AMR genes, transposon and plasmid replicons on the cd-hit defined cluster representative sequence (that is, the longest contig in the cluster as per the cd-hit algorithm) in the ten most common contig-clusters. These ten clusters were found in 248/714 (35%) of samples. We used BLAST blastn with the curated ARG-ANNOT database used by SRST2 (ref. ^47^) for AMR genes, ISfinder database^52^ for insertion sequences and PlasmidFinder database^48^ for plasmid replicons, selecting the best match by bitscore for a given location. In these databases, insertion sequences and AMR genes have a hierarchical identity structure for genes so, if there were multiple equally good matches from the same family, then a given gene was identified to family level, otherwise to individual insertion sequence or AMR gene. Inc group was determined for plasmid replicons. To understand differences between members of the same contig-cluster we generated multiple sequence alignments for each cluster by mapping all contig-clusters to the cluster reference using minimap2 v.2.16 (ref. ^53^) with the flags -ax asm. Nucleotide diversity (at each base) for contig-cluster multiple sequence alignment was calculated using the PopGenome v.2.7.5 package in R^54^ and coverage of the reference by each other contig extracted from the alignment SAM file and plotted for each alignment of that contig to identify synteny.

To track bacteria within- and between-participants we used map-to-reference pseudosequences: we defined popPUNK-clusters using the popPUNK v.2.0.2 tool^17^ and defined SNP-clusters as isolates with ≤5 whole-genome SNPs. The popPUNK algorithm uses *k*-mer distances to cluster genomes on the basis of a best-fitting model approach, thus ensuring phylogenetically robust and reproducible high-level grouping of related genomes^17^. In contrast, delineation of putative transmission clusters involves analysis of very closely related genomes separated by only a few SNPs, for example ≤25 SNPs^55^. We used pairwise SNPs calculated over the core genome and applied a conservative SNP threshold (≤5) selected such that members of an SNP-cluster could represent transmission events (given a mutation rate in *E. coli* of 10^−6^–10^−7^ per base per year or 1–5 SNPs per year across the genome^56,57^). We used snippy v.4.6.0 to map reads to K-12 MG1655 *E. coli* (ENA accession U00096) and MGH78578 *K. pneumoniae* (ENA accession GCA_000016305.1) references and to call SNPs with default settings, including excluding sites with depth <10 as low coverage. The *E. coli* map-to-reference pseudosequences had a mean (s.d.) coverage and depth of 92% (2%) and 58× (8×) respectively, with only a median (IQR) 0.9% (0.4–1.5%) bases per genome excluded as low coverage and SNPs called at a mean (s.d.) depth of 55× (15×). The *K. pneumoniae* complex genomes had a mean (s.d.) coverage of 92% (3%) and 52× (16×), with median 0.4% (0.3-1%) low-coverage bases excluded per genome and SNPs called at a mean (s.d.) depth of 51× (21×). We then used popPUNK v.2.0.2 on these assemblies, forming a new database with minimum *k-*mer size 15 (and otherwise default settings) and clustering with the DBSCAN algorithm. Clusters and distributions of *k*-mer distance are shown in Supplementary Figs. 7 and 8. To compare SNP distances between samples, we used these snippy-generated assemblies to construct a multiple sequence alignment, filtered regions of presumed recombination with gubbins v.3.0.0 (ref. ^58^) and calculated pairwise SNP distances using snp-dist v.0.6.2 (https://github.com/tseemann/snp-dists) and considered two isolates with five or fewer SNPs difference across the genome to be likely to represent the same isolate. We hence used this SNP difference to define a ‘SNP-cluster’, clustering isolates with hierarchical clustering using the function stats::hclust in R. We performed sensitivity analysis and varied this SNP threshold from 0 to 10.

### Statistical analysis

All statistical analyses were carried out in R v.4.0.2. Summaries of variables are presented as proportions (with exact binomial CIs where appropriate) or medians with IQRs. Kruskal–Wallis and Fisher’s exact tests were used to test the equivalence of patient characteristics across the three study groups for continuous and categorical variables, respectively. Associations of baseline ESBL-E carriage were assessed using logistic regression, including all variables that were felt a priori to be associated with ESBL-E carriage as predictors and presenting results as odds ratios for predictor variables with 95% CIs.

To assess within-participant conservation of organism, popPUNK-cluster, contig-cluster and SNP-cluster, we plotted within-participant correlation curves, including all participants who were colonized with *E. coli* or *K. pneumoniae* at time *t* = 0 then using non-parametric LOESS regression as implemented in the R stats::loess function with parameters *n* = 80, span = 0.75 to estimate the proportion at a time *t* later who were colonized with the same organism, popPUNK-cluster, contig-cluster or SNP-cluster. To assess the probability of two within-participant samples containing the same cluster by chance we compared the within-participant cluster conservation proportion to the proportion of between-sample participants that contained the same cluster. Odds ratios with 95% CIs were used to assess the odds of within-participant conservation of popPUNK-cluster and contig-cluster together or each alone compared to between-participant conservation.

We assessed for hospital-associated lineages by mapping metadata to the core-gene trees, defining isolates as either in-hospital (if they were isolated from a sample taken in hospital), recent discharge (if they were isolated from a sample taken up to 120 d following hospital admission) or community (if they were neither in-hospital nor recent discharge). We tested the hypothesis that popPUNK- and contig-clusters are healthcare-associated by comparing the proportion of in-hospital and healthcare-associated isolates (defined as in-hospital or recent discharge) for each cluster to the proportion of the remaining samples, using a Bonferroni-corrected Fisher’s exact test.

We looked for putative transmission clusters by plotting SNP-clusters using the R packages igraph v.1.2.11 (ref. ^59^) and ggraph v.2.0.5. We used Fisher’s exact test to compare the proportion of isolates that were community-associated between isolates that were members of an SNP-cluster and those that were not.

### Modelling of ESBL-E carriage

#### Defining the likelihood of the model

To understand the dynamics of ESBL-E carriage, we extended the continuous-time Markov models as implemented in the MSM^14^ package in R. MSM allows stepwise constant time-varying continuous-time Markov models, whereas we aimed to assess the biologically plausible effect of allowing antimicrobial exposure to act with a non-stepwise time-varying effect.

We assumed a two-state system with *N* participants, where at time *t* participant *n* will be in a state $S_n(t)$—either ESBL-E colonized ($S_n(t) = 1$) or ESBL-E uncolonized ($S_n(t) = 0$). For each participant *n* we assume a measured value of $S_n(t)$ at $i_n$ time points, the times of which are given by $t_j^n,j = 1,2 \ldots i_n$ and so the $i_n$ values of $S_n(t_j^n),j = 1,2 \ldots i_n$ are known.

If we develop a model with parameters *θ* that predicts the probability of a particular participant being in a state $S_n(t_b)$ at a time point $t_b$, given that they were in a state $S_n(t_a)$ at an earlier time point $t_a$, then the likelihood of this observation is:

1 $$P(S_n(t_b)|S_n(t_a),\theta )$$

Where | indicates ‘conditional on’ as per standard probability notation. Assuming that all observations are independent, then the likelihood for any participant is the product of all the transitions for that participant; and the likelihood of the data we observe is the product of all transitions for all participants:

2 $$\mathop {\prod }\limits_{n = 1}^N \mathop {\prod }\limits_{k = 2}^i P(S_n(t_k^n)|S_n(t_{k - 1}^n),\theta )$$

We assume a Markov model as the data-generating process, where the instantaneous probability of transition from a state *i* to state *j* is given by $q_{ij}$ or traditionally in matrix notation as the Q-matrix^14,60^ (for a two-state system):

3 $$\mathbf{Q}\left( t \right) = \left( {\begin{array}{*{20}{c}} {q_{00}\left( t \right)} & {q_{01}\left( t \right)} \\ {q_{10}\left( t \right)} & {q_{11}\left( t \right)} \end{array}} \right) = \left( {\begin{array}{*{20}{c}} { - \lambda \left( t \right)} & {\lambda \left( t \right)} \\ {\mu \left( t \right)} & { - \mu \left( t \right)} \end{array}} \right)$$

where we have defined $\lambda (t)$ as the instantaneous rate of ESBL-E loss and $\mu (t)$ as the instantaneous rate of ESBL-E gain and used the fact that the rows of the Q-matrix must sum to one (that is, every participant has to be in one state or another). Bold face here is used for matrices. If we define the probability of a participant being in a state *i* at time 0 and a state *j* a time *t* as $p_{ij}(t) = P(t)$, then these probabilities are linked to the Q-matrix by the set of differential equations:

4 $$\frac{{\mathrm{d}\mathbf{P}(t)}}{{{\mathrm{d}}t}} = \mathbf{Q}(t)\mathbf{P}(t)$$

Or, simplified if participants start in a state 0 or 1 to:

5 $$\frac{{{\mathrm{d}}P_0(t)}}{{{\mathrm{d}}t}} = - \lambda (t)P_0(t) + \mu (t)P_1(t)$$

6 $$\frac{{{\mathrm{d}}P_1(t)}}{{{\mathrm{d}}t}} = \lambda (t)P_0(t) - \mu (t)P_1(t)$$

where $P_i(t)$ is the probability of being in state *i* at time *t*. These differential equations can be solved with numerical ordinary differential equation solvers for all state transitions and all patients to calculate the likelihood.

#### Incorporating covariates

Following msm and ref. ^60^ we incorporated covariates with a proportional hazard approach where the *k* covariates $x_k,k = 1,2 \ldots k$ can act upon the hazard of transition via:

7 $$\lambda (t) = \lambda _0{\mathrm{exp}}(\beta _1x_1(t) + ... + \beta _kx_k(t))$$

8 $$\mu (t) = \mu _0{\mathrm{exp}}(\alpha _1x_1(t) + ... + \alpha _kx_k(t))$$

where the $x_k$ take the value 0 when an exposure is absent and 1 when it is present—this is the stepwise constant model. Parameters $\lambda _0$ and $\mu _0$ are the instantaneous rate of ESBL-E loss and the instantaneous rate of ESBL-E gain, respectively, with all covariates set to 0. The parameters *β* and *α* can therefore be thought of as the log transform of the hazard ratio of ESBL-E loss and gain, respectively; and the parameters $\lambda _0$ and $\mu _0$ can be interpreted as the reciprocal of the mean time in the uncolonized or colonized state respectively with all covariates set to 0.

Finally, the motivation for developing this model was to allow a time-varying effect of antimicrobial exposure. Assuming that antimicrobial exposure begins at time $t_{{\mathrm{start}}}$ and ends at $t_{{\mathrm{end}}}$, the value of the covariate $x_{{\mathrm{antimicrobial}}}(t)$ takes the form of an exponential decay following exposure:

9 $$x_{{\mathrm{antimicrobial}}} = \left\{ {\begin{array}{*{20}{c}} 0 \\ 1 \\ {{\mathrm{exp}}\frac{{ - \left( {t - t_{{\mathrm{end}}}} \right)}}{\gamma }} \end{array}} \right.\begin{array}{*{20}{c}} {0 < t < t_{{\mathrm{start}}}} \\ {t_{{\mathrm{start}}} \le t \le t_{{\mathrm{end}}}} \\ {t > t_{{\mathrm{end}}}} \end{array}$$

where the parameter *γ* is the half-life of the decay of antimicrobial exposure effect, multiplied by the natural log of 2.

#### Fitting and comparing models

The models were coded and fit in a Bayesian framework in Stan v.2.19 (ref. ^61^) accessed via the Rstan v.2.19.2 interface in R and plotted using the bayesplot v.1.8 R package. All code and data to fit the models are contained in the blantyreESBL^35^ v.1.2 R package available at https://github.com/joelewis101/blantyreESBL. Weakly informative priors were used; a normal distribution with mean 0 and s.d. 2 for *α* and *β* (corresponding to a hazard ratio of 7.4), a normal distribution with mean 0 and s.d. 0.2 for *μ* and *λ* and a normal distribution with a mean of 0 and s.d. of 50 d for *γ*. In each case, models were fit with four chains of 1,000 iterations each with 500 warmup iterations. Convergence was evaluated by inspection of traceplots and the Gelman–Rubin statistic^62^ being close to 1. Posterior estimates of parameters were expressed as medians with 95% CrIs generated from the quantiles of the posterior, excluding warmup iterations. We fit two models: one with the stepwise constant covariates and one with exponentially decaying effect of antimicrobial exposure.

To compare between the two models we used leave-one-out cross-validation as implemented in the loo v.2.1.0 package in R^63^, quantifying model fit with an estimate of the ELPD and comparing models with the ELPD difference and standard error of the difference, where a difference in ELPD of greater than two times the standard error of the difference could be interpreted as evidence in favour of the better-fitting model^63^. We also used graphical posterior predictive checks, simulating the predicted prevalence of ESBL-E across the three arms of the study by generating a probability of ESBL-E carriage for each participant at each time point for each posterior samples (excluding warmup draws) and sampling from a Bernoulli distribution using the predicted probability. We simulated from the posterior by fixing covariate values, assuming a baseline prevalence of 50% ESBL carriage at *t* = 0 and using all posterior draw covariate values (excluding warmup draws) and solving the likelihood differential equations using the R package deSolve v.1.28 (ref. ^64^) to generate daily predicted probabilities of carriage at time *t*, with 95% CrIs defined by simple quantiles. Mean person-days of colonization were estimated by calculating the area under these time–probability curves using the DescTools v.0.99 R package. In sensitivity analysis to explore the effect of non-ceftriaxone antimicrobials in driving ESBL-E carriage we refit the best-fitting model but disaggregated antimicrobial exposure into ceftriaxone and non-ceftriaxone antimicrobials, then proceeded as above.

### Reporting summary

Further information on research design is available in the Nature Research Reporting Summary linked to this article.

## Supplementary information

Supplementary Information Supplementary Tables 1–4 and Figs. 1–26.

Reporting Summary

Supplementary Data Accession numbers of all included genomic data linked to metadata.

## Data Availability

All data to reproduce this analysis are available as the blantyreESBL v.1.2 R package available at https://joelewis101.github.io/blantyreESBL/ and on a mirrored Zendo repository (10.5281/zenodo.5554081). Reads from all isolates sequenced as part of this study have been submitted to the European Nucleotide Archive under project IDs PRJEB26677, PRJEB28522 and PRJEB36486 and accession numbers linked to metadata are available in the R package as well as in the Supplementary Data.

## Code availability

All data to reproduce this analysis are available as the blantyreESBL v.1.2 R package available at https://joelewis101.github.io/blantyreESBL/ and on a mirrored Zendo repository (10.5281/zenodo.5554081).
