# Colonisation dynamics of extended spectrum beta-lactamase-producing Enterobacterales in the gut of Malawian adults

**DOI:** 10.1038/s41564-022-01216-7

**Published:** 2022-09-05

**Authors:** Joseph M. Lewis, Madalitso Mphasa, Rachel Banda, Mathew A. Beale, Eva Heinz, Jane Mallewa, Christopher Jewell, Brian Faragher, Nicholas R. Thomson, Nicholas A Feasey

**Affiliations:** 1 Malawi-Liverpool Wellcome Clinical Research Programme, Blantyre, Malawi; 2 Liverpool School of Tropical Medicine, Liverpool, UK; 3 University of Liverpool, Liverpool, UK; 4 Wellcome Sanger Institute, Hinxton, UK; 5 Department of Medicine, College of Medicine, University of Malawi; 6 University of Lancaster, Lancaster, UK; 7 London School of Hygiene and Tropical Medicine, London, UK

**Keywords:** Whole-genome sequencing, Africa south of the Sahara, Drug resistance, microbial

## Abstract

Drug-resistant bacteria of the order Enterobacterales which produce extended-spectrum beta-lactamase enzymes (ESBL-Enterobacterales: ESBL-E) are global priority pathogens. Antimicrobial stewardship interventions proposed to curb their spread include shorter courses of antimicrobials to reduce selection pressure, but individual-level acquisition and selection dynamics are poorly understood. We sampled stool of 425 adults (aged 16-76) in Blantyre, Malawi, over six months and used multi-state modelling and whole-genome sequencing to understand colonisation dynamics of ESBL-E. Models suggest a prolonged effect of antimicrobials such that truncating an antimicrobial course at two days has a limited effect in reducing colonisation. Genomic analysis shows largely indistinguishable diversity of healthcare-associated and community-acquired isolates, hence some apparent acquisition of ESBL-E during hospitalisation may instead represent selection from a patient’s microbiota by antimicrobial exposure. Our approach could help guide stewardship protocols; interventions that aim to review and truncate courses of unneeded antimicrobials may be of limited use in preventing ESBL-E colonisation.

## Introduction

Antimicrobials are one of the most successful therapies available to modern medicine, but the spread of antimicrobial resistance (AMR) is a threat to their effective use. Significant global effort is being directed at antimicrobial stewardship programmes which the World Health Organisation considers a key tool in reducing AMR^1^. Antimicrobial stewardship at the individual level often emphasises rationalisation of antimicrobials through narrowing their spectrum of action as soon as possible after commencement of broad empiric antimicrobial therapy in severely unwell individuals. The time frame (e.g., 48 hours) for this is typically pragmatically selected to match likely availability of diagnostic test results. Rationalisation of therapy is partly based on the assumption that it will reduce emergence of AMR, but the mechanism by which antimicrobial exposure acts at the individual level to promote colonisation and/or infection with resistant pathogens, and the dynamics of colonisation and decolonisation, are not well understood^2–5^. Improved understanding of the dynamics of individual-level AMR-acquisition under antimicrobial pressure can therefore inform the design of stewardship protocols.

One setting in which antimicrobial stewardship is a significant challenge is in the treatment of severe febrile illness in the low- and middle-income countries of sub-Saharan Africa (sSA). In Blantyre, Malawi, for example, as in much of sSA, limited availability of diagnostics results in prolonged courses of broad-spectrum antimicrobials – largely ceftriaxone, a third-generation cephalosporin (3GC) antibiotic^6^ - for severe febrile illness. Ceftriaxone has been extensively used since its introduction to the Malawian national formulary in 2005^7^. but this has been associated with an increase in 3GC resistance^8^, particularly in bacteria of the order Enterobacterales. This is largely mediated by extended spectrum beta-lactamase (ESBL) enzymes^8–10^. ESBL-producing Enterobacterales (henceforth ESBL-E) are an increasing public health challenge throughout much of sSA ^11, 12^, and often have few or no locally available treatment options; in Blantyre, 91% of invasive *K*. *pneumoniae* are now 3GC resistant^8^, and strategies to reduce ESBL-E infections are needed.

Gut mucosal colonisation with ESBL-E is thought to precede invasive infection, is common across sSA, and has often been found to be associated with prior hospitalization and/or antimicrobial exposure^12,13^. An improved mechanistic understanding of colonisation dynamics following these exposures therefore has the potential to inform evidence-based interventions to reduce colonisation and hence opportunity for transmission. Here we present the results from a clinical study of longitudinal ESBL-E carriage in Blantyre, Malawi, sampling adults as they pass through the hospital and are exposed to antimicrobials. We use multi-state modelling^14^ and whole-genome sequencing as a high-resolution bacterial typing tool to describe and understand the dynamics of ESBL-E colonisation.

## Results

### Antimicrobial exposure drives increase in ESBL-E prevalence

**Extended Data Figure 1:**
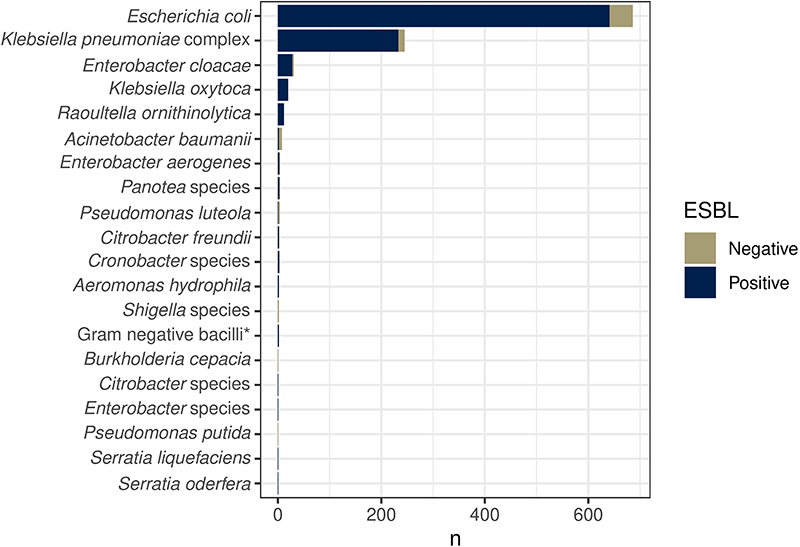
Species of bacteria isolated from stool. Samples were labelled “Gram negative bacilli” if they could not be speciated using the API system.

**Extended Data Figure 2:**
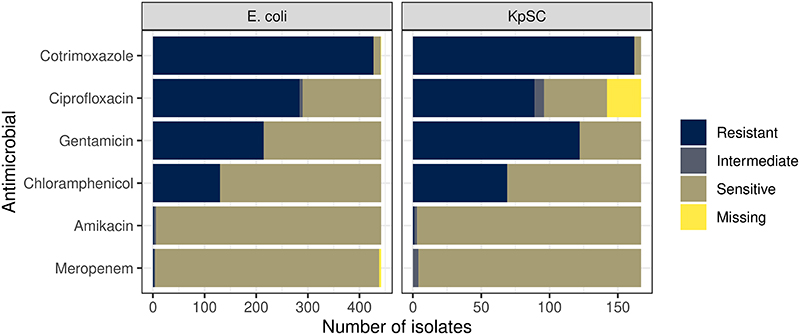
Results of antimicrobial sensitivity testing (AST). AST of cultured *E*. *coli* and *K*. *pneumoniae* sequence complex (KpSC) isolates using the disc diffusion method. A subset of isolates (442/473 E. coli and 167/203 KpSC) underwent AST.

**Figure 1:**
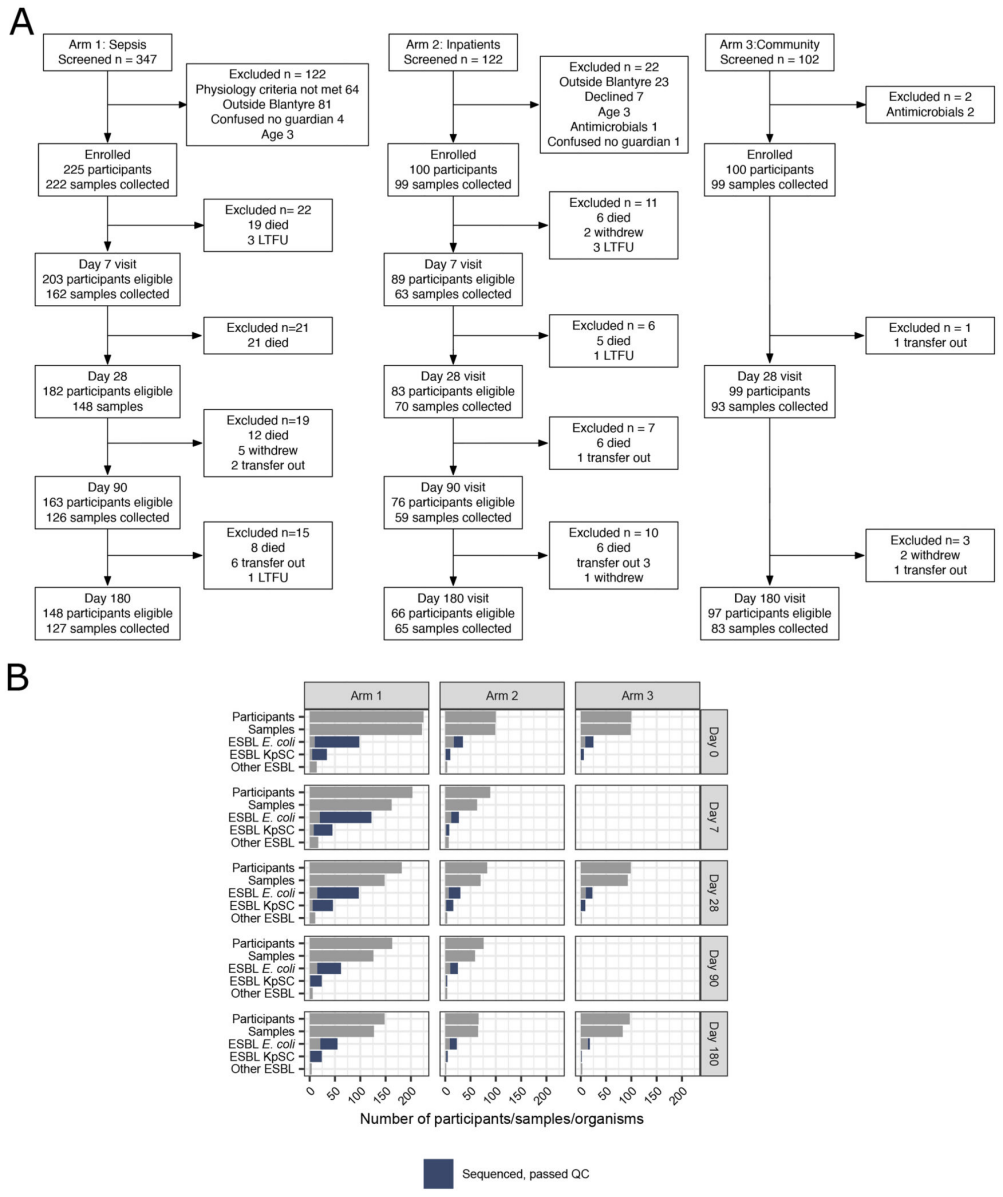
Flow of patients through study (A) and number of samples collected at each time point (B) showing number of samples in which ESBL *E. coli, Klebsiella pneumoniae* sequence complex or other species were identified were identified and how many *E*. *coli* or *Klebsiella pneumoniae* sequence complex isolates were sequenced and passed QC (blue colouring of bar). ESBL.= Extended spectrum beta lactamase; KpSC = *Klebsiella pneumoniae* sequence complex; QC = Quality Control.

Between 19^th^ February 2017 and 2^nd^ October 2018, we recruited 425 adults; i) 225 patients with sepsis and antimicrobial exposure, admitted to Queen Elizabeth Central hospital (QECH) Blantyre; ii) 100 antimicrobial-unexposed inpatients and iii) 100 antimicrobial-unexposed community participants (Table 1). There were 1631 study visits, with successful stool or rectal swab collection at 1417/1631 (87%) visits; missing samples were equally distributed across all study arms and visits (Figure 1, Supplementary Figure 1). At least one ESBL-E species was cultured in 723/1417 (51%) of samples. 1032 organisms were isolated, most commonly *Escherichia coli* (n = 686) and *Klebsiella pneumoniae* species complex (KpSC, n = 245, Figure 1, Supplementary Table 1 and Extended Data Figure 1). Phenotypic resistance to other antimicrobial classes (determined for 442 *E*. *coli* and 167 KpSC) was common (Extended Data Figure 2).

**Figure 2:**
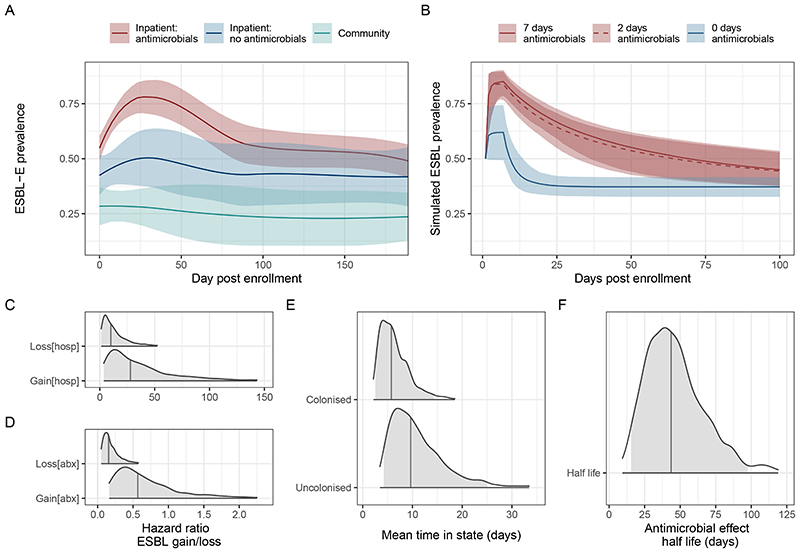
Prevalence and determinants of longitudinal ESBL-E carriage. **A:** ESBL prevalence stratified by the three study groups; inpatients exposed to antimicrobials (red), inpatients without antimicrobial exposure (blue), community members (green), showing sharp increase in prevalence following antimicrobial exposure. Prevalence is estimated using a LOESS non-parametric regression with 95% confidence interval. Community members are censored on antimicrobial exposure or hospitalization and antimicrobial-unexposed inpatients on antimicrobial exposure. **B:** simulated ESBL-E prevalence using final fitted model for a hypothetical cohort of patients with initial ESBL-E colonisation prevalence 50%, admitted to hospital for seven days and exposed to seven, two or zero days of antimicrobials, showing that there is little difference between seven and two days**. C-F:** posterior estimates of parameter values from final fitted model. Shaded grey areas shows 95% credible interval and grey vertical line shows median parameter estimate. **C-D:** hazard ratio of gain or loss of ESBL-E (expressed as natural logarithm) showing that antimicrobial exposure acts primarily to prolong carriage by reducing ESBL-E loss, whereas hospitalization acts to increase both gain and loss, with a net effect to increase prevalence. **E:** Mean time in colonised/uncolonised states with all covariate values set to 0 (i.e. no hospitalization or antimicrobial exposure). **F:** Half-life of effect of antimicrobial exposure, showing that antimicrobial exposure acts with a prolonged effect to prolong colonisation.

Baseline prevalence of ESBL-E colonization was 178/420 42% (95% CI 38-47%, Figure 2A and Supplementary Data Table 1). In multivariable modelling (Supplementary Data Table 2), co-trimoxazole preventative therapy exposure (CPT, administered lifelong for people living with HIV as per WHO guidelines) was associated with ESBL-E colonisation (aOR of colonisation 2.34 95% CI 1.00-5.66) as was use of unprotected water sources [aOR 2.96 95% CI 1.07-8.75], rainy season [aOR 2.21 95% CI 1.07-8.75], number of adults in the household [aOR 1.20 95% CI 1.03-1.40] and recent hospitalization [aOR 6.64 95% CI 1.98 – 30.75]).

**Extended Data Figure 3:**
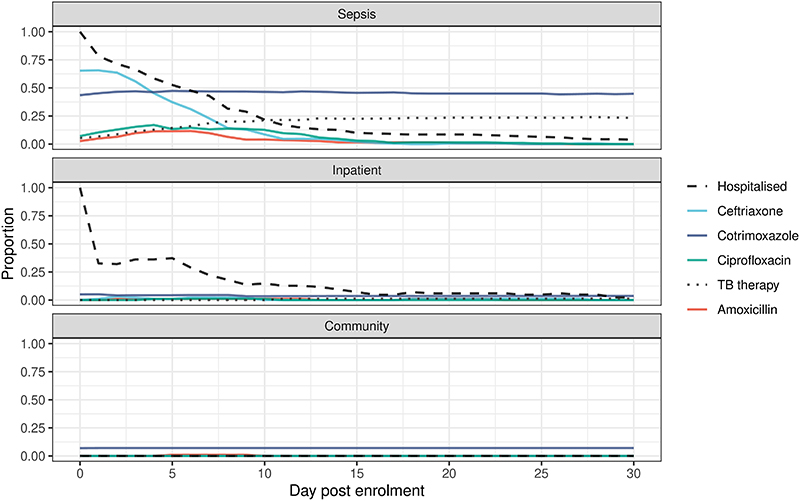
Participant antimicrobial exposure and hospitalisation. Stratified by study arm

Following enrolment, there was rapid increase in ESBL-E colonisation prevalence in antimicrobial exposed inpatients (109/222 [49%] day 0 to 127/162 [78%] day 7) compared to the antimicrobial-unexposed inpatients (41/99 [41%] day 0 to 32/62 [51%] day 7, Figure 2A and Supplementary Figure 2). Ceftriaxone was the most commonly received antimicrobial (183/225, 80%) followed by co-trimoxazole (110/225, 49%), ciprofloxacin (61/225 27%) and antitubercular chemotherapy (52/225, 23%), but person-days of co-trimoxazole exposure was higher because of chronic CPT administration (Extended Data Figure 3 and Supplementary Table 3). Median (IQR) length of hospital stay was longer in the antimicrobial-exposed (5 [IQR 2-10] days) compared to the antimicrobial-unexposed (2 [IQR 2-7] days) inpatient groups.

**Extended Data Figure 4:**
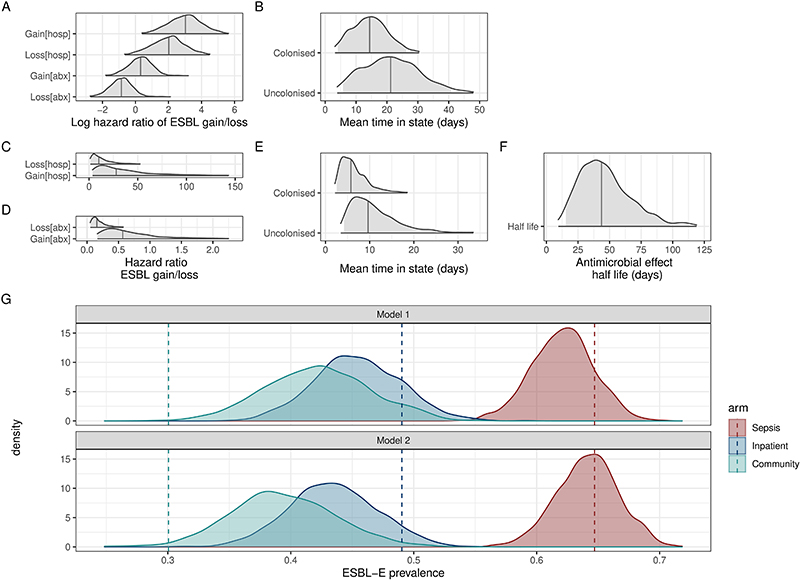
Comparing ESBL-E carriage models. Comparing a model of ESBL-E carriage that include a stepwise constant effect of hospitalisation and antimicrobial exposure (where the effect of covariates ceases when exposure ceases in Model 1) to a model that allows the effect of antimicrobial exposure to persist when exposure finishes, modelled as an exponential decay in Model 2. A-B: Parameter estimates from Model 1 expressed as natural logarithm of hazard ratio of gain or loss of ESBL-E for antimicrobial exposure [abx] and hospitalisation [hosp] (A) and mean time in the colonised or uncolonised state (B) with covariates set to 0 (i.e. no antimicrobials, not hospitalised). C-F: Parameter estimates from Model 2, with the same interpretation and the addition of the half-life (in days) of the decaying effect of antimicrobial exposure (F). G: Posterior parameter checks of two models showing actual prevalence of ESBL-E carriage stratified by study arm (dashed lines) with kernel density plots of predicted prevalence from fitted models, obtained by using all posterior parameter estimates (n=2000, discarding warmup iterations) to predict probability of ESBL-E from the actual data, and sampling from a binomial distribution using this probability. Model 1 underfits the antimicrobial-exposed arm of the study, which is improved by the addition of the prolonged effect of antimicrobials.

We used continuous-time multi-state Markov models to understand determinants of ESBL-E carriage, and to account for differences in exposures across the arms of the study. In this model, each patient is “colonised” or “non-colonised”, with the transition rate governed by a linear function of time-varying covariates (hospitalisation and antimicrobial exposure). When comparing a stepwise-constant covariate model (where the effect of hospitalization and antimicrobial exposure cease immediately as exposure ceases) to a model which included a prolonged effect of antimicrobial exposure, modelled as an exponential decay that continues to exert an effect when exposure ceases, the latter was a better fit to the data as assessed by leave-one-out cross validation (estimated expected pointwise log predictive value [ELPD] difference 10.5 [standard error 4.2] in favour of the exponential decay model) and posterior predictive checks (Extended Data Figure 4).

**Extended Data Figure 5:**
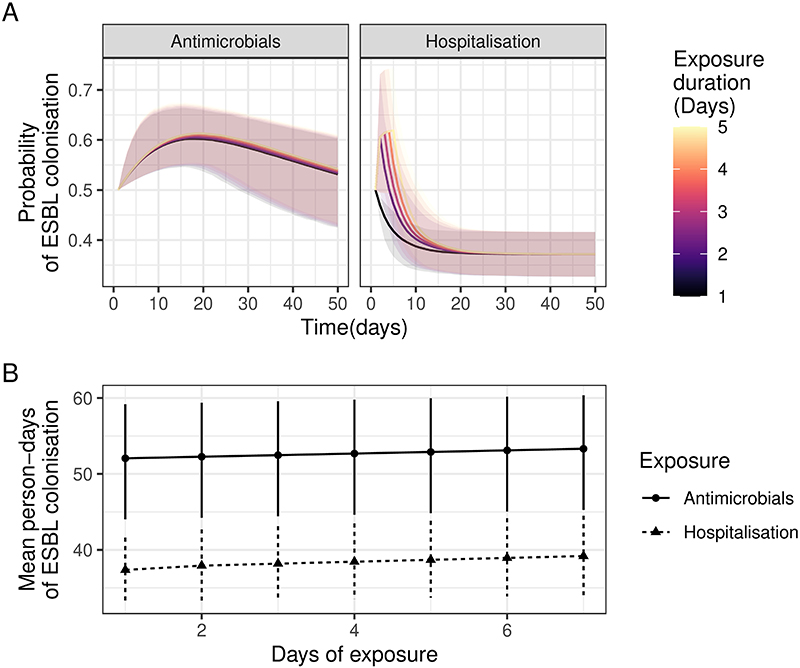
relative effects of hospitalisation and antimicrobial exposure. A: Predicted probability from final fitted model of ESBL colonisation assuming 0.5 probability of colonisation at time t=0 and between 1-5 days of antimicrobial exposure (left) or hospitalisation (right). B: Estimated mean person- days of colonisation for the exposures in A out to 100 days (i.e. the area under the curves in A top 100 days). In all panels confidence intervals are 95% confidence intervals; the full model posterior was used to estimate the quantities of interest and confidence intervals constructed from quantiles of estimates.

In this model, hospitalisation increased both ESBL-E gain and loss parameters resulting in a modest increase in overall carriage prevalence, whereas antibacterial therapy largely acted to prolong ESBL-E carriage by reducing loss and acted with a prolonged effect with half-life 43.7 (95% CrI 15.4-97.7) days (Figure 2C-E, Supplementary Data Table 4). Posterior plots of pairs of parameters revealed some non-identifiability between the gain and loss parameters, manifesting as correlation (Supplementary Figure 3). Overall, in terms of estimated person-days of colonisation, antimicrobial exposure had a greater effect than hospitalisation (Extended Data Figure 5). Posterior predictive simulations from the final fitted model (Figure 2B) considering a hypothetical seven-day hospital admission with seven, two, or zero days of antimicrobial therapy suggest that antimicrobial therapy and hospitalization act together to produce the observed rapid increase in ESBL-E, but that there is very little difference in ESBL-E prevalence carriage from truncating seven days of antimicrobial therapy to two days.

**Extended Data Figure 6:**
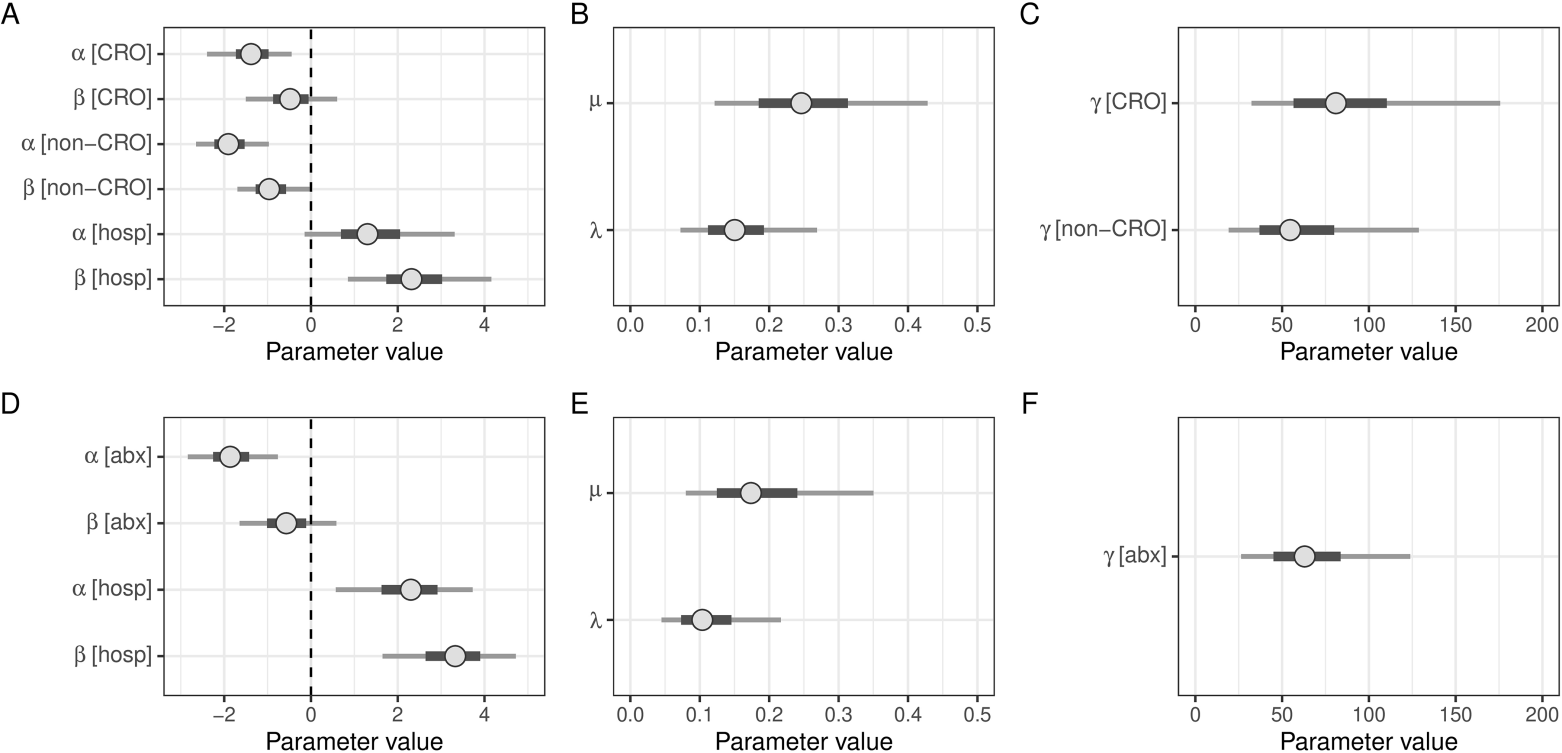
Parameter estimates from models considering ceftriaxone and non-ceftriaxone antimicrobials. (A-C) show parameter estimates from this model; (D-F) show original model. In each case, point shows posterior median and bar and whiskers show undertainty in parameter estimate by 50% and 95% credible interval, respectively, constructed from quantiles of the posterior estimates. Parameters are: a, loss parameters, b, gain parameters and g, decay parameter of effect of antimicrobials. [CRO] indicates that the parameter refers to ceftriaxone, [non-CRO] that the parameter refers to non-ceftriaxone antimicrobials, and [hosp] to hospitalisation.

In sensitivity analysis, we refit the final model but disaggregated antimicrobial exposure into ceftriaxone and non-ceftriaxone exposure. The effect of ceftriaxone was similar to non-ceftriaxone antimicrobials (Extended Data Figure 6) suggesting co-selection of ESBL-E carriage by exposure to non beta-lactam antimicrobials.

### Within-host ESBL persistence mechanism not horizontal gene transfer

**Extended Data Figure 7:**
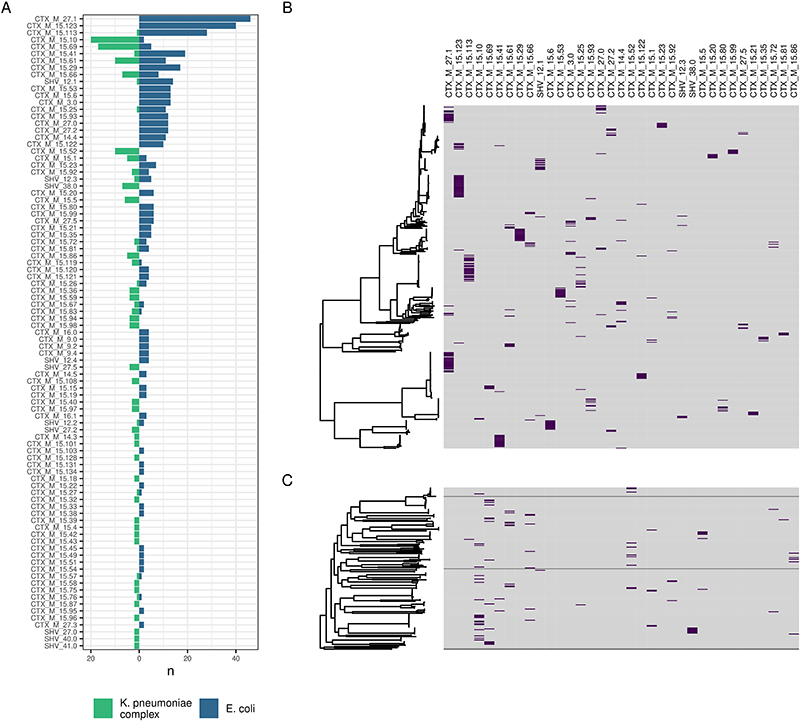
Distribution of contig-clusters between and within genera. (A) shows distribution of contig clusters by genus. (B-C) show contig-cluster presence (purple)-absence (grey) mapped back to core gene maximum likelihood phylogeny for *E*. *coli* (B) and *K*. *pneumoniae* subsp. *pneumoniae* (C)

Next we used short-read whole-genome sequencing to track bacteria and ESBL genes within study participants. Following quality control, 473 *E*. *coli* and 203 *K*. *pneumoniae* species complex (KpSC) genomes were included in the analysis with a median (IQR) 3 (2-4) *E*. *coli* isolates per participant from 230 participants and 2 (1-2) *KpSC* isolates per participant from 142 participants. Most (n=190) KpSC isolates were *K*. *pneumoniae* subsp. *pneumoniae*. An analysis of population structure, core gene phylogeny and AMR and plasmid gene content of these isolates has previously been made^15,16^ and AMR gene and plasmid incompatibility group (Inc-types) content is summarised in Supplementary Figures 4-6. To track bacteria within participant we mapped reads to reference genomes and defined high-level sequence clusters using popPUNK^17^, and SNP-clusters as isolates with whole-genome SNP distance ≤ 5. PopPUNK grouped *E*. *coli* into 87 clusters representing 58 sequence types (STs), and KpSC into 91 clusters representing 75 STs, 55 of these *K*. *pneumoniae* subsp. *pneumoniae* (Supplementary Figures 7-8). These clusters (henceforth popPUNK clusters) were largely concordant with the core-gene phylogenies (Supplementary Figure 9). To track ESBL genes and their genomic environment (because full plasmids usually fail to assemble into a single molecule with *de novo* assembly of short reads due to repeat-regions) we clustered *de novo* assembled contigs containing 3GC resistance genes using the cd-hit^18^ algorithm, including those from both KpSC and *E*. *coli*. 714 3GC-resistance gene containing contigs were identified in 672/676 samples; 18 different genes formed 195 clusters (henceforth, contig-clusters) of median size 1 (range 1-42, Supplementary Figure 10). They were genus- and lineage-associated (Extended Data Figure 7), though 21/195 (11%) of contig-clusters contained both *E*. *coli* and KpSC genomes. In sensitivity analysis, cluster membership was stable to increasing the sequence identity and length cut-off of the cd-hit algorithm (Supplementary Figure 11), though with some fragmentation of clusters apparent at sequence identity of 1.0. The nucleotide diversity and insertion sequence, AMR gene, and plasmid replicon content of the ten most common contig clusters (present in 248/714 [35%] of samples) is shown in Supplementary Figure 12-21. Generally, shorter assembled contigs terminated in insertion sequences (i.e. consistent with compound transposons), often IS26, and showed low nucleotide diversity to the portion of the cluster representative to which they were matched. Where nucleotide diversity was present, it was often flanking transposable elements, which could be consistent with transfer/rearrangement events. In some cases, the ESBL gene was assembled onto a contig with a plasmid replicon but this was not the norm.

**Figure 3:**
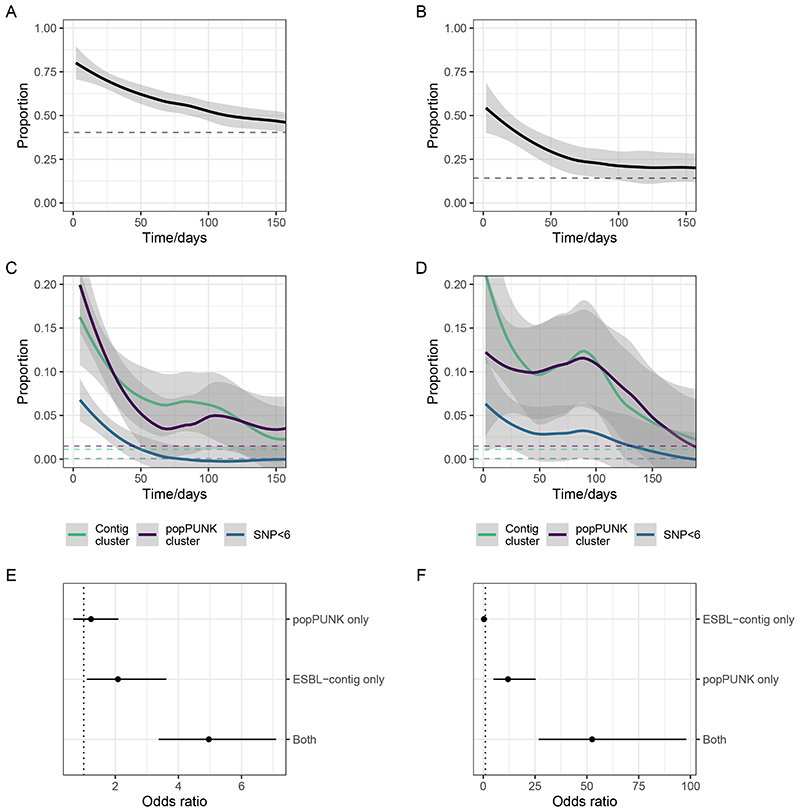
Within-participant dynamics of ESBL-E colonisation. A-B: Proportion of participants who, at time 0 have detectable ESBL producing *E*. *coli* (A) or *K*. *pneumoniae* sequence complex (KpSC) (B) who remain colonised as a function of time. Vertical dotted lines in each panel show the baseline proportion for the dataset of between-participant samples that contain the same genus. C-D: Proportion of participants with detectable ESBL producing *E*. *coli* (A) or KpSC (B) at time 0, who remain colonised with the same contig-cluster, popPUNK cluster, or an isolate of SNP distance ≤5 as a function of time with dotted lines showing the baseline (between-participant) proportion, as above. E-F: Odds ratio from logistic regression with 95% confidence intervals of within-participant sample pairs containing the same popPUNK cluster alone, contig-cluster alone, or both, compared to between-participant pairs, for *E*. *coli* (E) and KpSC (F) showing that the element that is most likely to be conserved is the popPUNK cluster-contig cluster combination. This analysis (E-F) uses the sample pair as the unit of analysis so each sample may be included more than once.

For participants colonised with *E*. *coli* or KpSC at a time t=0, the probability of remaining colonised returned to a baseline by 100-150 days (Figure 3A-B), but the probability of remaining colonised with the same contig-cluster or popPUNK cluster was lower, and the probability of remaining colonised with an organism differing by five or fewer SNPs was lower still (Figure 3C-D), suggesting significant within-participant strain diversity. Nevertheless, a temporal signal was present: two samples closer together in time were more likely to contain the same popPUNK, contig- and SNP-cluster, enabling us to seek hospital-associated transmission events. Sensitivity analysis varying the definition of SNP cluster from 0 to 20 SNPs did not alter these conclusions (Supplementary Figure 22). Comparing within-patient sample pairs to between-patient sample pairs the popPUNK-cluster contig-cluster combination was conserved more than either popPUNK-cluster or contig-cluster alone (Figure 3E-F), consistent with the hypothesis that within-participant persistence of ESBL, where it occurs, is caused by persistence of ESBL-containing bacteria rather than horizontal gene transfer and persistence of ESBL genes.

### Hospital linked lineages/transmission clusters are unusual

**Figure 4:**
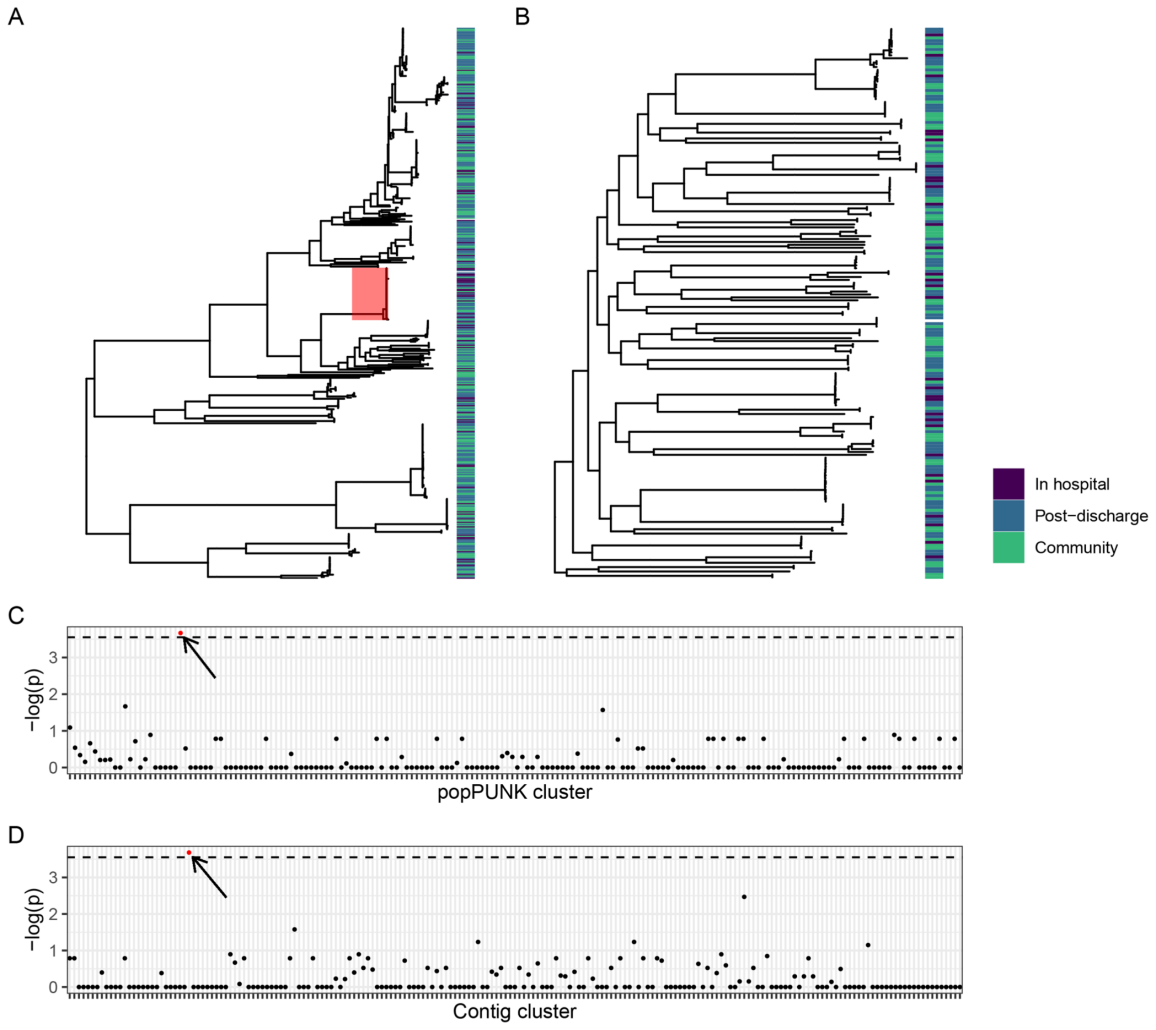
Hospital association of popPUNK clusters and contig-clusters. A-B: Maximum-likelihood core gene phylogenetic tree for *E*. *coli* (A) and *K*. *pneumoniae* subsp. *pneumoniae* (B) showing in-hospital (black), post discharge (dark grey) and community (light grey) isolates, where post-discharge is defined as up to 120 days post hospital discharge. Hospital-associated samples are distributed across the tree, but only the popPUNK cluster highlighted in red shows an association with in-hospital isolation. C-D: Manhattan plots showing p-value of two-sided Fisher’s exact test for association of popPUNK cluster (C) and contig-cluster (D) with in-hospital isolation. Dotted line shows Bonferroni-corrected value corresponding to p = 0.05. Only one popPUNK cluster is significantly associated with in-hospital isolation (highlighted in red on the plot, C, and core gene tree, A) at this level. Similarly, one contig-cluster is associated with in-hospital isolation, highlighted in red; this is the contig-cluster which is associated with the hospital-associated lineage.

Next, we examined any hospital association of popPUNK clusters. In-hospital and post-discharge isolates were distributed throughout the core-gene phylogenies and only one popPUNK cluster contained more hospital isolates than would be expected by chance following correction for multiple comparisons (Figure 4A-C). This corresponded to *E*. *coli* ST410. Similarly, one contig-cluster was associated with in-hospital isolation (Figure 3D); this *bla*^CTX-M-15^ containing contig-cluster was primarily associated with *E*. *coli* ST410 (CTX_M_15.113 in Extended Data Figure 6B). Sensitivity analysis aggregating in-hospital and post-discharge isolates to a “healthcare associated” category did not identify any distinctly healthcare-associated popPUNK or contig clusters (Supplementary Figure 23).

**Figure 5:**
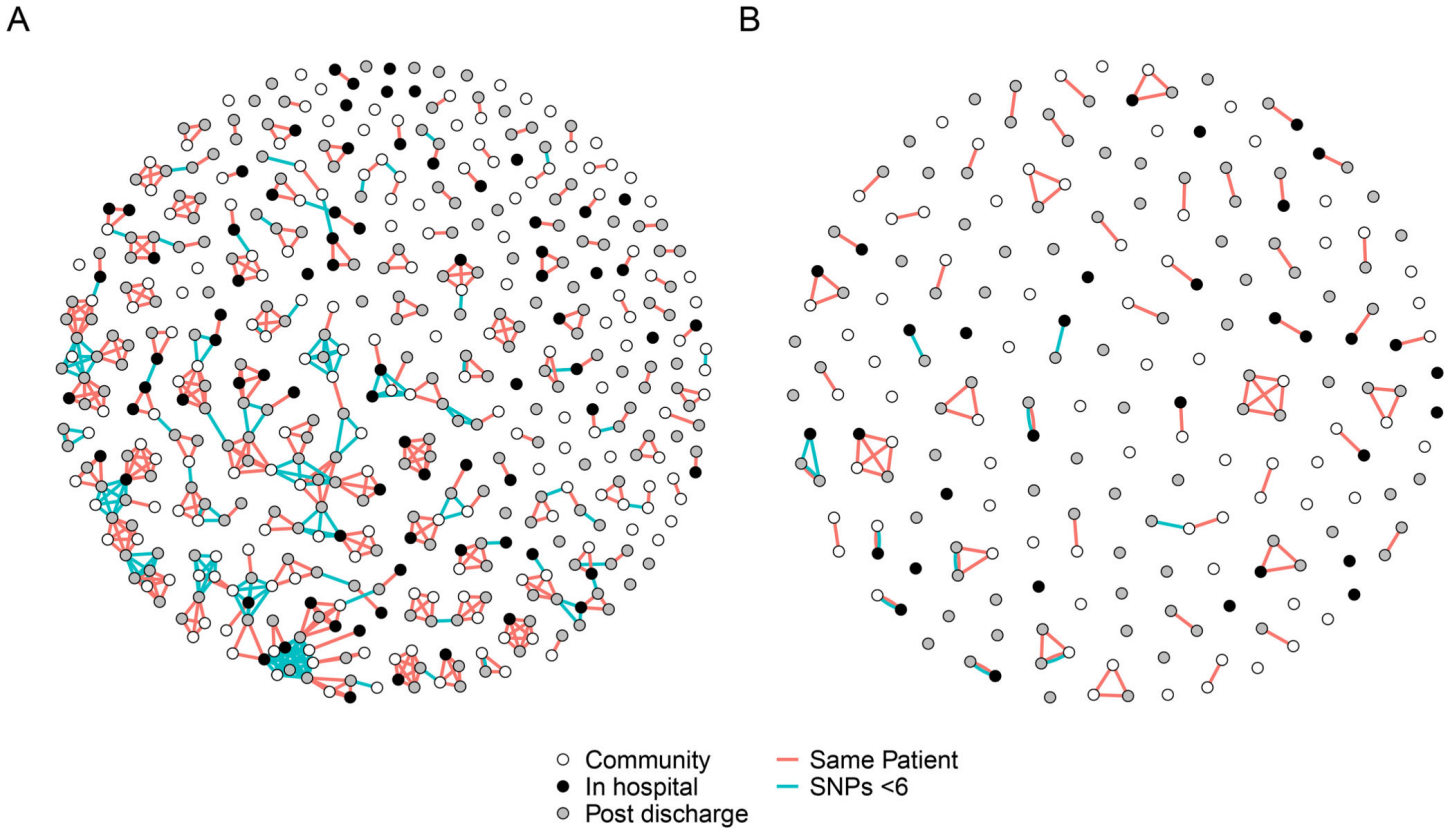
Network plot of SNP-clusters (putative transmission clusters) for *E*. *coli* (A) and *K*. *pneumoniae* sequence complex (B) showing that putative transmission clusters are not exclusively hospital associated. Points are samples, coloured by place of isolation (in-hospital [black], community [white], or up to 120 days post-discharge [grey]). Red lines link samples that are within a single participant. Blue lines link samples that are differ by five or fewer SNPs. The plot shows that most samples are not members of a SNP-cluster; that most SNP clusters encompass samples from different (rather than multiple samples from the same) participants; and that SNP-clusters are not exclusively hospital-associated, i.e. they contain in-hospital, community, and post-discharge samples.

As hospital associated popPUNK clusters were infrequent, we investigated putative hospital-related transmission SNP-clusters which could represent transmission clusters. 151/473 (32%) *E*. *coli* and 21/203 (10%) KpSC were members of a SNP-cluster and hence represent possible transmission events (Figure 5). The clusters were generally small (median size 2 [IQR 2-5] for *E*. *coli* and 2 [IQR 2-3] for KpSC) and, in *E. coli,* largely contained samples from different participants rather than the same participant: only 6% (10/175) of pairwise comparisons of within-SNP-cluster *E*. *coli* samples were from the same participant. Fewer KpSC formed a SNP-cluster but more were from the same participant (58% [7/12]) rather than between participants. Most clusters (149/192 [78%] *E*. *coli* and 31/57 [54%] KpSC) contained two or more healthcare associated isolates, which might represent transmission events. However, the proportion of samples that were members of a SNP-cluster were similar between healthcare associated isolates and community isolates. For *E*. *coli* 54/171 (32%) of community isolates versus 96/300 (32%) of healthcare-associated isolates were members of a SNP cluster (p = 1.00, Fisher’s exact test). For KpSC 4/74 (5%) of community isolates versus 17/128 (13%) of healthcare associated isolates were members of a SNP cluster (p = 0.15). This is not consistent with widespread hospital-associated transmission above the level of community transmission. Sensitivity analysis varying the SNP threshold from 0 to 10 did not significantly alter the conclusions (Supplementary Figures 24-26).

## Discussion

Combining longitudinal sampling, multi-state modelling and whole genome sequencing, we describe the dynamics of ESBL-E colonisation in Malawian adults. These findings advance our understanding of the effects of antimicrobial exposure on AMR-acquisition, with potentially significant implications for the directions of future research into the design of both antimicrobial stewardship and infection prevention and control interventions.

First, baseline sampling provides insight into drivers of ESBL-E colonisation in Blantyre. ESBL-E colonisation is very common and identification of community risk factors for baseline colonisation, suggests significant community transmission consistent with other studies across sSA^12^ and elsewhere^19^. Observed associations with unprotected water use for drinking and higher prevalence in rainy season suggest inadequate access to water, sanitation and hygiene (WASH) infrastructure and/or WASH behavioural practices may be contributing, and associations of colonisation with household crowding suggests within-household transmission.

Second, novel Markov models fitted to longitudinal sampling data allow insight into the dynamics of ESBL-E colonisation. We demonstrate a rapid increase in ESBL-E colonisation following hospital admission and antimicrobial exposure. Modelling suggests that both hospitalisation and antimicrobial exposure may act to drive this increase, though antimicrobial exposure has a greater effect by exerting an effect long after antimicrobial exposure finishes, with a half-life of 43.7 (95% CrI 15.4-97.7) days. Simulations suggest that, due to the sustained effect of antimicrobials, short courses of antimicrobials could exert a similar effect to prolonged courses in terms of ESBL-E carriage. This finding has clear implications for antimicrobial stewardship protocols, suggesting that truncating courses of antimicrobials may have limited effect on ESBL-E carriage compared to avoiding antimicrobial administration altogether. In addition, non-ceftriaxone antimicrobials exerted a similar effect on ESBL-E carriage as ceftriaxone; this, along with the high prevalence of resistance to other antimicrobial classes in these isolates, suggests that co-selection for ESBL-E by non-beta-lactam agents is occurring. Hence switching ceftriaxone to other classes of agent in treatment protocols may have a limited effect on ESBL-E carriage in this setting.

Previous ESBL-E longitudinal sampling and modelling studies examining the effect of antimicrobials on colonization have examined community and post-travel carriage in adults in the Netherlands^2,3,5^ and transmission of ESBL-E in neonatal units in the high prevalence setting of Cambodia^20^. In the former studies, some association of ESBL-E carriage with antimicrobial exposure was found, but antimicrobial exposure was not common, further, sampling was neither intensive nor linked to antimicrobial exposure to fully define the effects. In a Cambodian neonatal unit, antimicrobial therapy was robustly linked to an increased daily probability of acquiring *K*. *pneumoniae* colonisation but long-term sampling was not available to define post-antimicrobial effects as we have done here. Further work to understand the dynamics of ESBL-E colonisation and decolonisation under antimicrobial pressure to guide stewardship efforts should be a priority in other settings to guide antimicrobial stewardship programmes.

More broadly, these findings highlight a need to define and measure clinically relevant individual-level AMR-carriage as an endpoint in trials of antimicrobial treatment strategies. An expanding evidence base has demonstrated equivalence of clinical outcomes in a variety of clinical infection syndromes for shorter versus longer courses of antimicrobial therapy^21^, but a non-linear relationship between antimicrobial exposure and colonisation with AMR-bacteria (as we demonstrate here) may mean that seven compared to fourteen days of antimicrobials (for example) has little benefit in reducing ESBL-E colonisation. Defining clinically relevant AMR endpoints for trials and measuring them in order to understand their relationship with antimicrobial exposure is thus crucial for optimising the way in which antimicrobials are used in clinical practice.

Third, using whole-genome sequencing as a high-resolution typing tool allowed us to explore the mechanism by which antimicrobials promote ESBL-E carriage. This is a key question: antimicrobials could either act to promote healthcare-associated transmission by reducing colonisation resistance, or select for low abundance resistant bacteria that were already present in the microbiota but not detected by bacterial culture on admission. Hospitalisation exerted an effect to increase ESBL-E colonisation in the absence of antimicrobial exposure, suggesting that transmission within the hospital is occurring. However, we found limited support for hospital associated lineages or hospital-associated transmission clusters above the level of transmission in the community. This suggests either that ESBL-E acquisition had occurred in the community and was enriched by antimicrobial exposure in hospital; that the diversity of isolates transmitted in the hospital was represented by the diversity of isolates in the community (a distinct possibility in our setting); or that our sparse sampling strategy missed hospital-associated transmission events. Genomic epidemiology studies of ESBL-E colonisation and infection clearly demonstrate that true healthcare associated transmission of ESBL-E occurs^22–24^ but few studies have longitudinal sampling pre-, during- and post- antimicrobial exposure. Defining the contribution of antimicrobial selection versus novel ESBL-E acquisition events following antimicrobial exposure will guide prevention efforts and should be a priority for future studies – as should understanding the way in which antimicrobials may act to reduce colonisation resistance and aid transmission. Healthcare-associated transmission could be reduced by infection prevention and control procedures, but antimicrobial selection pressure amplifying minority ESBL-E carried prior to hospital admission would need novel strategies to protect the microbiota against selection for ESBL-E such as antimicrobial binding compounds^25^ or oral beta lactamases^26^.

We demonstrate considerable within-participant ESBL-E bacterial diversity (as defined by SNP- and popPUNK-clusters) over time even in participants who remain colonised with the same genus; a further key question is whether this temporal bacterial diversity with preserved ESBL-E colonisation could represent horizontal gene transfer of ESBL genes between bacteria. Horizontal gene transfer could also explain an apparent lack of hospital-associated transmission clusters, if ESBL genes disseminated into diverse clones in the healthcare setting. We find that within-participant the popPUNK-cluster contig-cluster combination was conserved more than either popPUNK-cluster or contig-cluster alone, consistent with the hypothesis that within-participant persistence of ESBL, where it occurs, is caused by persistence of ESBL-containing bacteria rather than horizontal gene transfer of ESBL genes to differing bacterial hosts. This does not support the hypothesis of horizontal gene transfer as primary mechanism of ESBL temporal persistence within-participant on the timescale of the study.

There are limitations to our study. Most importantly, due to resource limitation, we took only one colony pick from each patient-time point sample for sequencing and so we may have missed intra-host ESBL-E diversity^27,28^, and hence underestimated the numbers of transmission clusters. We used short read sequencing and clustered ESBL-containing contigs as a proxy for mobile genetic elements, but our approach likely under-represents transfer/rearrangement events in the flanking contexts around ESBL genes. Hence inferring that inclusion in a specific contig-cluster represents a single stable/consistent genetic construct should be done very cautiously. We used an arbitrary SNP threshold of 5 SNPs to define SNP-clusters, a strong assumption, which could misclassify isolates; this cut-off (empirically derived) has been used by public health bodies in England and Canada to define possible *E*. *coli* outbreaks^29,30^. We used a map-to-reference approach to identify core-genome SNPs that could have introduced bias due to the choice of reference. We have looked at high-level clustering with popPUNK and it may be that a high-resolution clustering approach using local, lineage-specific references would give the resolution to identify more hospital associated transmission events. The models of AMR carriage assumed a 100% sensitivity and specificity of sampling, which may not be valid. We were not able to disaggregate the effect of different antimicrobial agents because of the sample size. We did not collect data on sibling or family connections between hospitalised and community participants which could explain apparent community links. For hospitalised patients, we did not sample the ward environment, carers, staff, food or toilets; and our sampling strategy was sparse. In the analysis of associations of ESBL-E colonisation at enrolment, we relied on self-reported antimicrobial exposure and hospitalisation and it is possible in our setting where people may take medication without knowing the exact nature of it and that this may differ from true antimicrobial exposure. Hospitalisation records were not available to exclude the possibility that antimicrobials were received, which could explain some of the association between hospitalisation and ESBL-E colonisation at enrolment.

In conclusion, we describe the dynamics of ESBL-E colonisation in Malawian adults as they are exposed to both antimicrobial therapy and hospitalization. Antimicrobial therapy and hospitalisation act rapidly to promote ESBL-E colonisation. Antimicrobial therapy exerts a prolonged effect which means that truncated courses of antimicrobials may have a similar effect to longer ones, which has implications for stewardship protocols. Short-read whole-genome sequencing did not identify widespread, distinct hospital associated lineages or that putative hospital-associated transmission clusters were more common than community SNP-clusters. Future work should define dynamics of intra-host ESBL-E diversity under antimicrobial pressure, using longitudinal sampling, metagenomic sequencing methods to describe diversity and long-read sequencing to characterize mobile genetic elements. This will facilitate development of clinically relevant AMR endpoints for clinical trials and the development of a sound evidence base for stewardship protocols at the individual level – an evidence base which is currently lacking.

## Methods

### Study setting and design

The study took place in Queen Elizabeth Central Hospital (QECH), Blantyre, Malawi, a government tertiary referral hospital for the Southern Region of Malawi and the only hospital providing free healthcare to the ~800,000 residents^31^ of urban Blantyre. Nursing ratios at QECH are usually around two trained nurses to a 60 patient ward and basic nursing care and is provided by family members; food is supplied to all patients on the ward by the hospital and each ward has one toilet which is shared by all patients. Malawi is a low-income country in southeast Africa, with an estimated adult human immunodeficiency virus (HIV) prevalence of 9% (UNAIDS, Malawi Country Profile https://www.unaids.org/en/regionscountries/countries/malawi) and a high tuberculosis incidence of 133/100 000 person-years^32^. Blantyre has a subtropical climate with a rainy season from November to April.

Adults (> 15 years) with sepsis, defined by fever and organ dysfunction criteria, were recruited from the emergency department of QECH 0700-1700 Monday to Friday as part of a study of sepsis aetiology, as described elsewhere^33^. Two comparator cohorts of participants were recruited: age and sex matched adults from QECH emergency department who had a plan from their attending clinical team to admit to hospital but no plan for antimicrobial administration; and community members matched by age, sex and home location to recruited sepsis patients. Exclusion criteria for the latter two groups were antimicrobial exposure within the past four weeks (except co-trimoxazole preventative therapy [CPT] and antituberculous chemotherapy); hospitalised participants who lacked capacity to give informed consent and had no guardian to give proxy consent; participants who spoke neither English nor Chichewa; and participants who lived > 30km from Blantyre city. Geographic matching on home location between community members and sepsis patients was achieved by random walk from the houses of sepsis participants with initial direction established by spinning a bottle on the floor. Written informed consent was obtained from all participants. An admission questionnaire was administered to all participants at enrolment and hospitalised patients were reviewed daily by a study team member until discharge to extract details of antimicrobial therapy from the clinical record. All clinical decisions were at the discretion of the attending clinical team. Further review by the study team occurred at day 7, 28, 90 and 180, except for community members in whom the day 7 and 90 visits were omitted. If participants failed to come to their scheduled visits, then they were traced by telephone or, if that failed, by home visit. Hospitalised patients were not financially compensated for their time, but all other participants were at a rate of 500MWK for home visits and 2000MWK for hospital visits. Data were captured using a combination of direct electronic data entry by study team members onto tablet devices (ODK^34^, Get ODK inc. United States) and paper forms (TeleForm, Opentext, Canada).

### Ethical and data availability statements

The study was approved by the research ethics committees of the Liverpool School of Tropical Medicine (16-062) and Malawi College of Medicine (P.11/16/2063). All data and code to replicate this analysis are available as the *blantyreESBL* R v1.2^35^ package at https://github.com/joelewis101/blantyreESBL. All sequence data generated for this study has been deposited in the European Bioinformatics Archive (EBI); accession numbers linked to metadata are available in the R package and are available as supplementary data to this manuscript.

### Microbiologic methods

At each study visit (enrolment, day 7, 28, 80 and 190 for hospitalised participants and enrolment, day 28 and 190 for community members) stool was collected in a sterile polypropylene pot; if a participant was not able to provide a stool sample, then a rectal swab was taken by a trained study team member and stored in Amies medium for transport. Stool and rectal swab samples were stored at 4°C before being batch processed weekly: samples were plated directly onto commercially available ESBL selective chromogenic agar (CHROMagar ESBL, CHROMagar, France) and cultured aerobically overnight. Morphologically distinct white or blue colonies were speciated with the API 20E system (Biomerieux, France); pink colonies were identified as *E*. *coli*. ESBL production was confirmed with the combination disc method on iso-sensitest agar with discs of cefotaxime (30 micrograms) and ceftazidime (30 micrograms) with and without clavulanic acid (10 micrograms), with ESBL production confirmed if there was a difference of 5mm or more between the clavulanic acid and non-clavulanic acid discs for either cephalosporin. For organisms likely to carry a chromosomal *bla_amp_c* beta-lactamase gene and hence able to hydrolyse cefotaxime and ceftazidime (defined for our purposes as *Enterobacter* spp., *Citrobacter freundii*, *Morganella morganii*, *Providencia stuartii*, *Serratia* spp., *Hafnia alvei*); cefipime (30 micrograms), an AmpC-stable cephalosporin was used with and without clavulanic acid (10 micrograms), and ESBL production confirmed if there was a difference of 5mm or more between the clavulanic acid and non-clavulanic acid discs. For a subsample of isolates, antimicrobial sensitivity testing (AST) using the disc diffusion method on iso- sensitest agar following British Society for Antimicrobial Chemotherapy (BSAC) guidelines (https://bsac.org.uk/) was carried out for meropenem, amikacin, chloramphenicol, ciprofloxacin, co-trimoxazole and gentamicin. The first 442 E. coli and 167 K. pneumoniae sequence complex isolates cultured in the study (this number determined by resource and logistic considerations) underwent AST.

### DNA extraction, sequencing and bioinformatic analysis

Due to resource and logistic constraints, not all samples could be taken forward for sequencing: 503/686 *E*. *coli* and 217/233 *K. pnemoniae* sequence complex isolates were randomly selected from the collection for sequencing. One of each morphologically distinct *K*. *pneumoniae* species complex and *E*. *coli* colony, respectively, from each selected sample was taken forward for DNA extraction and whole-genome sequencing. DNA was extracted from overnight nutrient broth culture using the Qiagen DNA mini kit as per the manufacturer’s instructions. Extracted DNA was shipped to the Wellcome Sanger Institute to undergo whole-genome sequencing using Illumina HiSeq X10 to produce 150bp paired end reads. Quality control, de-novo assembly and construction of core gene phylogeny are described elsewhere^15,16^; in brief, species was confirmed with Kraken v0.10.6 and Bracken v1.0^36^ before de-novo assembly with SPAdes v3.14^37^, with the modifications described by Page et al.^38^ and annotation with prokka v1.5^39^ using a genus specific database from RefSeq. The Roary v1.17 pan-genome pipeline^40^ were used to identify core genes, considering genes contained in at least 99% isolates to be core. Samples with assembly failure (< 4Mb assembled length) and samples with > 10% contamination (as defined by CheckM v1.1.3^41^) were excluded from the analysis. 203 KpSC and 473 *E*. *coli* genomes passed QC and were included in the analysis. A core gene multiple sequence alignment was generated using mafft v7.205^42^, SNP-sites identified using SNP-sites v2.4.1^43^ and the resultant SNP alignment (99,693 variable sites from a core gene alignment of 1.39Mb bases for *E*. *coli* and 378,596 variable sites from a 2.82Mb core gene alignment for *K*. *pneumoniae* complex) used to infer a maximum-likelihood phylogenetic tree using IQ-TREE v1.6.3^44^ with and the ModelFinder module, which selected the generalised time reversible model with FreeRate heterogeneity with 5 parameters for *E*. *coli* and 8 parameters for *K*. *pneumoniae* complex. 1000 ultrafast bootstrap replicates were generated. Trees were visualized with ggtree v2.2.4^45^.

AMR genes and plasmid replicons were identified using ARIBA v2.14.6 ^46^ and the curated ARG-ANNOT database used by SRST2^47^ and PlasmidFinder^48^ databases, respectively, on the sequence reads. ARIBA was also used to identify multilocus sequence type (ST) using the 7-gene *Klebsiella*^49^ and 7-gene Achtman^50^ *E*. *coli* schemes hosted at pubMLST (https://pubmlst.org/). Since individual AMR genes from mobile genetic elements can exist in a variety of genomic contexts (e.g. chromosomal, different plasmid backbones, etc), we clustered ESBL-containing contigs from the *de novo* assemblies (identified with BLAST blastn v2.7.1^51^ using the curated ARG-ANNOT database used by SRST2) to form contig-clusters using cd-hitest v4.8.1^18^ with 95% sequence identity and otherwise default settings. We varied cd-hit sequence identity from 95% to 100% and the length cut-off parameter from 0 to 0.8 (i.e. for length cut-off *x,* cluster members must be at least a fraction *x* of the longest cluster member) in sensitivity analysis. To understand the genomic environment of the ESBL gene in these contig-clusters we identified AMR genes, transposon and plasmid replicons on the cd-hit defined cluster representative sequence (i.e. the longest contig in the cluster as per the cd-hit algorithm) in the ten most common contig-clusters. These ten clusters were found in 248/714 (35%) of samples. We used BLAST blastn with the curated ARG-ANNOT database used by SRST2^47^ for AMR genes, ISfinder database^52^ for insertion sequences and PlasmidFinder database^48^ for plasmid replicons, selecting the best match by bitscore for a given location. In these databases insertion sequences and AMR genes have a hierarchical identity structure for genes so if there were multiple equally good matches from the same family then a given gene was identified to family level, otherwise to individual insertion sequence or AMR gene. Inc group was determined for plasmid replicons. To understand differences between members of the same contig-cluster we generated multiple sequence alignments for each cluster by mapping all contig clusters to the cluster reference using minimap2 v2.16^53^ with the flags -ax asm. Calculated nucleotide diversity (at each base) for contig cluster multiple sequence alignment were calculated using the PopGenome v2.7.5 package in R^54^ and coverage of the reference by each other contig extracted from the alignment SAM file and plotted for each alignment of that contig to identify synteny.

To track bacteria within- and between-participant we used on map-to-reference pseudosequences: we defined popPUNK clusters using the popPUNK v2.0.2 tool ^17^ and defined SNP-clusters as isolates with ≤ 5 whole-genome SNPs. The popPUNK algorithm uses kmer distances to cluster genomes based on a best-fitting model approach, thus ensuring phylogenetically robust and reproducible high-level grouping of related genomes^17^. In contrast, delineation of putative transmission clusters involves analysis of very closely related genomes separated by only a small number of SNPs, e.g. ≤25 SNPs^55^. We used pairwise SNPs calculated over the core genome, and applied a conservative SNP threshold (≤ 5) selected such that members of a SNP-cluster could represent transmission events (given a mutation rate in *E*. *coli* of 10^-6^ -10^-7^ per base per year or 1-5 SNPs per year across the genome^56,57^).. We used snippy v4.6.0 to map reads to K-12 MG1655 *E*. *coli* (ENA accession U00096) and MGH78578 *K*. *pneumoniae* (ENA accession GCA_000016305.1) references, and to call SNPs with default settings, including excluding sites with depth < 10 as low coverage. The *E*. *coli* map-to-reference pseudosequences had a mean (SD) coverage and depth of 92% (2%) and 58x (8x) respectively, with only a median (IQR) 0.9% (0.4-1.5%) bases per genome excluded as low coverage and SNPs called at a mean (SD) depth of 55x (15x). The *K*. *pneumoniae* complex genomes had a mean (SD) coverage of 92% (3%) and 52x (16x), with median 0.4% (0.3-1%) low coverage bases excluded per genome and SNPs called at a mean (SD) depth of 51x (21x). We then used popPUNK v2.0.2 on these assemblies, forming a new database with minimum kmer size 15 (and otherwise default settings) and clustering with the DBSCAN algorithm. Clusters and distributions of kmer distance are shown in Supplementary Figures 7 and 8. To compare SNP distances between samples, we used these snippy-generated assemblies to construct a multiple sequence alignment, filtered regions of presumed recombination with gubbins v3.0.0^58^ and calculated pairwise SNP distances using snp-dist v0.6.2 (https://github.com/tseemann/snp-dists) and considered two isolates with 5 or fewer SNPs difference across the genome to be likely to represent the same isolate. We hence used this SNP difference to define a “SNP-cluster”, clustering isolates with hierarchical clustering using the function *stats::hclust* in R. We performed sensitivity analysis and varied this SNP threshold from 0 -10.

### Statistical analysis

All statistical analyses were carried out in R v4.0.2 (R foundation for statistical computing, Vienna, Austria). Summaries of variables are presented as proportions (with exact binomial confidence intervals where appropriate) or medians with interquartile ranges. Kruskal-Wallis and Fisher’s Exact tests were used to test the equivalence of patient characteristics across the three study groups for continuous and categorical variables, respectively. Associations of baseline ESBL-E carriage were assessed using logistic regression, including all variables that were felt *a priori* to be associated with ESBL-E carriage as predictors, and presenting results as odds ratios for predictor variables with 95% confidence intervals.

To assess within-participant conservation of organism, popPUNK cluster, contig-cluster, and SNP-cluster, we plotted within participant correlation curves, including all participants who were colonised with *E*. *coli* or *K*. *pneumoniae* at time t = 0 then using non-parametric LOESS regression as implemented in the R *stats::loess* function with parameters n= 80, span = 0.75 to estimate the proportion at a time t later who were colonised with the same organism, popPUNK cluster, contig-cluster, or SNP-cluster. To assess the probability of two within-participant samples containing the same cluster by chance we compared the within-participant cluster conservation proportion to the proportion of between-sample participants that contained the same cluster. Odds ratios with 95% confidence intervals were used to assess the odds of within-participant conservation of popPUNK cluster and contig-cluster together or each alone compared to between-participant conservation.

We assessed for hospital associated lineages by mapping metadata to the core gene trees, defining isolates as either in-hospital (if they were isolated from a sample taken in hospital) recent discharge (if they were isolated from a sample taken up to 120 days following hospital admission) or community (if they were neither in-hospital nor recent discharge). We tested the hypothesis that popPUNK and contig clusters are healthcare associated by comparing the proportion of in-hospital and healthcare-associated isolates (defined as in-hospital or recent discharge) for each cluster to the proportion of the remaining samples, using a Bonferroni-corrected Fisher’s exact test.

We looked for putative transmission clusters by plotting SNP clusters using the R packages *igraph v1.2.11^59^* and *ggraph v2.0.5*. We used Fisher’s exact test to compare the proportion of isolates that were community-associated between isolates that were members of a SNP-cluster and those that were not.

### Modelling of ESBL-E carriage

#### Defining the likelihood of the model

To understand the dynamics of ESBL-E carriage, we extended the continuous time Markov models as implemented in the MSM^14^ package in R. MSM allows stepwise constant time-varying continuous time Markov models, whereas we aimed to assess the biologically plausible effect of allowing antimicrobial exposure to act with a non-stepwise time-varying effect.

We assumed a two-state system with *N* participants, where at time *t* participant *n* will be in a state *S_n_*(*t*) - either ESBL-E colonised (*S_n_*(*t*) = 1) or ESBL-E uncolonised (*S_n_*(t) = 0). For each participant *n* we assume have a measured value of *S_n_*(*t*) at *i* time points, the times of which are given by $t_{j}^{n}$, *j* = 1, 2…*i_n_,* and so the i_n_ values of $S_{n}(t_{j}^{n})$, *j* = 1,2…*i_n_* are known.

If we develop a model with parameters *θ* that predicts the probability of a particular participant being in a state *S_n_*(*t_b_*) at a time point *t_b_* given that they were in a state *S_n_*(*t_a_*) at an earlier time point *t_a_* then then the likelihood of this observation is:

[Equation 1] $$P(S_{n}(t_{b})|S_{n}(t_{a}),\theta )$$

Where | indicates “conditional on” as per standard probability notation. Assuming that all observations are independent then the likelihood for any participant is the product of all the transitions for that participant; and the likelihood of the data we observe is the product of all transitions for all participant:

[Equation 2] $$\prod_{n=1}^{N}\prod_{k=2}^{i}\left(S_{n}(t_{k}^{n})|S_{n}(t_{k-1}^{n}),\theta \right)$$

We assume a Markov model as the data-generating process, where the instantaneous probability of transition from a state *i* to state *j* is given by *q*_ij_, or traditionally in matrix notation as the Q-matrix^14,60^ (for a two state system):

[Equation 3] $$Q(t)=\left(\begin{array}{cc} q_{00}(t) & q_{01}(t)\\ q_{10}(t) & q_{11}(t) \end{array}\right)=\left(\begin{array}{cc} -\lambda (t) & \lambda (t)\\ \mu (t) & -\mu (t) \end{array}\right)$$

Where we have defined *λ*(*t*) as the instantaneous rate of ESBL-E loss, and *μ*(*t*) as the instantaneous rate of ESBL-E gain, and used the fact that the rows of the Q-matrix must sum to one (i.e. every participant has to be in one state or another). If we define the probability of a participant being in a state *i* at time 0 and a state *j* a time *t* as *p_ij_*(*t*) = *P*(*t*), then these probabilities are linked to the Q-matrix by the set of differential equations:

[Equation 4] $$\frac{dP(t)}{dt}=Q(t)\cdot P(t)$$

Or, simplified if participants start in a state 0 or 1 to:

[Equation 5] $$\frac{dP_{0}(t)}{dt}=-\lambda (t)P_{0}(t)+\mu (t)P_{1}(t)$$

[Equation 6] $$\frac{dP_{1}(t)}{dt}=\lambda (t)P_{0}(t)-\mu (t)P_{1}(t)$$

Where *P_i_*(*t*) is the probability of being in state *i* at time *t*. These differential equations can be solved with numerical ordinary differential equation (ODE) solvers for all state transitions and all patients to calculate the likelihood.

#### Incorporating covariates

Following *msm* and Marshall and Jones^60^ we incorporated covariates with a proportional hazard approach where the *k* covariates *x_k_*, *k* = 1,2 … *k* can act upon the hazard of transition via:

[Equation 7] $$\lambda (t)=\lambda_{0}\text{exp}\left(\beta_{1}x_{1}(t)+\ldots +\beta_{k}x_{k}(t)\right)$$

[Equation 8] $$\mu (t)=\mu_{0}\text{exp}\left(\alpha_{1}x_{1}(t)+\ldots +\alpha_{k}x_{k}(t)\right)$$

Where the *x_k_* take the value 0 when an exposure is absent and 1 when it is present – this is the stepwise constant model. *λ*_0_ and *μ*_0_ are the instantaneous rate of ESBL-E loss, and the instantaneous rate of ESBL-E gain, respectively, with all covariates set to 0. The parameters *ß* and *a* can therefore be thought of as the log transform of the hazard ratio of ESBL-E loss and gain, respectively; and the parameters λ_0_ and μ_0_ can be interpreted as the reciprocal of the mean time in the uncolonised or colonised state respectively with all covariates set to 0.

Finally, the motivation for developing this model was to allow a time-varying effect of antimicrobial exposure. Assuming that antimicrobial exposure begins at time *t_start_* and ends at *t_end_*, the value of the covariate *x_antimicrobial_* (*t*) takes the form of an exponential decay following exposure:

[Equation 9] $$x_{antimicrobial\;}=\{\begin{array}{cc} 0 & 0<t<t_{start\;}\\ 1 & t_{start\;}\leq t\leq t_{end\;}\\ \text{exp}\;\frac{-\left(t-t_{end\;}\right)}{\gamma } & t>t_{end\;} \end{array}$$

Where the parameter *γ* is the half life of the decay of antimicrobial exposure effect, multiplied by the natural log of 2.

#### Fitting and comparing models

The models were coded and fit in a Bayesian framework in Stan v2.19^61^ accessed via the *Rstan* v2.19.2 interface in R, and plotted using the *bayesplot* v1.8 R package. All code and data to fit the models is contained in the blantyreESBL^35^ v1.2 R package available at https://github.com/joelewis101/blantyreESBL. Weakly informative priors were used; a normal distribution with mean 0 and standard deviation 2 for alpha and beta (corresponding (corresponding to a hazard ratio of 7.4), a normal distribution with mean 0 and standard distribution 0.2 for mu and lambda and a normal distribution with a mean of 0 and standard deviation of 50 days for gamma. In each case models were fit with four chains of 1000 iterations each with 500 warmup iterations. Convergence was evaluated by inspection of traceplots and the Gelman-Rubin statistic^62^ being close to 1. Posterior estimates of parameters were expressed as medians with 95% credible intervals generated from the quantiles of the posterior, excluding warmup iterations. We fit two models: one with the stepwise-constant covariates and one with exponentially-decaying effect of antimicrobial exposure.

To compare between the two models we used leave one out cross validation as implemented in the *loo* v2.1.0 package in R^63^, quantifying model fit with an estimate of the expected log predictive density (ELPD) and comparing models with the ELPD difference and standard error of the difference, where a difference in ELPD of greater than two times the standard error of the difference could be interpreted as evidence in favour of the better fitting model^63^. We also used graphical posterior predictive checks, simulating the predicted prevalence of ESBL-E across the three arms of the study by generating a probability of ESBL-E carriage for each participant at each time point for each posterior samples (excluding warmup draws) and sampling from a Bernoulli distribution using the predicted probability. We simulated from the posterior by fixing covariate values, assuming a baseline prevalence of 50% ESBL carriage at t=0 and using all posterior draw covariate values (excluding warmup draws) and solving the likelihood differential equations using the R package *deSolve* v1.28^64^ to generate daily predicted probabilities of carriage at time t, with 95% confidence intervals defined by simple quantiles. Mean person-days of colonisation were estimated by calculating the area under these time-probability curves using the DescTools v0.99 R package. In sensitivity analysis to explore the effect of non-ceftriaxone antimicrobials in driving ESBL-E carriage we refit the best fitting model but disaggregated antimicrobial exposure into ceftriaxone and non-ceftriaxone antimicrobials, then proceeded as above.

## Extended Data

## Supplementary Material

Extended Data Figure Legends

Sample accession numbers

Supplementary Data

## Figures and Tables

**Table 1:** Baseline characteristics of included participants

Variable	Sepsis, receiving antibiotics (n=225)	Inpatient, not receiving antibiotics (n=100)	Community, not receiving antibiotics (n=100)	p	Total (n = 425)
**Demographics**					
Age (yrs)	35.9 (27.8-43.5)	40.4 (29.1-48.3)	32.5 (24.0-38.4)	<0.001	35.6 (26.9-43.9)
Male	114/225 (51%)	51/100 (51%)	40/100 (40%)	0.163	205/425 (48%)
**HIV status**					
HIV-positive	143/225 (64%)	12/100 (12%)	18/100 (18%)	<0.001	173/425 (41%)
HIV-negative	70/225 (31%)	77/100 (77%)	22/100 (22%)		169/425 (40%)
HIV Unknown	12/225 (5%)	11/100 (11%)	60/100 (60%)		83/425 (20%)
ART status^*^					
Current CPT	98/141 (70%)	5/12 (42%)	7/18 (39%)	0.013	110/171 (64%)
Current ART	117/143 (82%)	9/12 (75%)	18/18 (100%)	0.082	144/173 (83%)
Months on ART	28.7 (3.7-72.6)	35.1 (2.9-79.8)	31.5 (13.0-79.9)	0.698	29.5 (3.8-72.8)
**Healthcare exposure**					
Antibiotics within 28 days^†^	60/225 (27%)	0/100 (0%)	0/100 (0%)	<0.001	60/425 (14%)
Hospitalised within 28 days	18/225 (8%)	1/100 (1%)	0/100 (0%)	<0.001	19/425 (4%)
Current TB treatment	10/225 (4%)	0/100 (0%)	4/100 (4%)	0.083	14/425 (3%)
**Household**					
Number of adults	2.0 (2.0-3.0)	3.0 (2.0-4.0)	2.0 (2.0-4.0)	0.907	3.0 (2.0-4.0)
Number of children	2.0 (1.0-3.0)	2.0 (1.0-3.0)	2.0 (1.0-3.0)	0.395	2.0 (1.0-3.0)
Keep animals	71/225 (32%)	43/100 (43%)	15/100 (15%)	<0.001	129/425 (30%)
*Poultry*	*46/71 (65%)*	*34/43 (79%)*	*10/15 (67%)*		*90/129 (70%)*
*Dogs*	*18/71 (25%)*	*11/43 (26%)*	*9/15 (60%)*		*38/129 (29%)*
*Goats*	*12/71 (17%)*	*7/43 (16%)*	*1/15 (7%)*		*20/129 (16%)*
*Other*	*3/71 (4%)*	*6/43 (14%)*	*0/15 (0%)*		*9/129 (7%)*
Electricity in house	119/225 (53%)	41/100 (41%)	58/100 (58%)	0.041	218/425 (51%)
Flush toilet^‡^	14/225 (6%)	5/100 (5%)	1/100 (1%)	0.110	20/425 (5%)
Protected water source^§^	216/225 (96%)	92/100 (92%)	98/100 (98%)	0.124	406/425 (96%)
Treat drinking water with chlorine	19/225 (8%)	5/100 (5%)	0/100 (0%)	0.004	24/425 (6%)

P vales are from two sided Fisher’s Exact test or Kruskal-Wallis for categorical or continuous variables, respectively. ART = Antiretroviral therapy, CPT = Co-trimoxazole preventative therapy, TB = tuberculosis. Numeric variables are presented as median (IQR) and categorical variables as proportions. P-values are from Fisher’s exact test or Kruskal-Wallis tests (categorical or continuous variables respectively) across the three groups; p-value for HIV status compares distribution of HIV status across the three groups. In some cases denominator may be less than the total number of participants due to missing data.

* Denominator for ART status is HIV reactive participants only

† Excluding TB treatment and CPT

‡ Flush toilet vs latrine (pit or hanging) or no toilet

§ Protected water source includes borehole, water piped into or outside dwelling or public standpipe; unprotected sources include surface water or unprotected springs.

## Data Availability

All data to reproduce this analysis are available as the blantyreESBL v1.2 R package available at https://joelewis101.github.io/blantyreESBL/, and on a mirrored Zendo repository (https://doi.org/10.5281/zenodo.5554081). Reads from all isolates sequenced as part of this study have been submitted to the European Nucleotide Archive under project IDs PRJEB26677, PRJEB28522 and PRJEB36486 and accession numbers linked to metadata are available in the R package as well as as supplementary data to this manuscript.

## Code Availability

All data to reproduce this analysis are available as the blantyreESBL v1.2 R package available at https://joelewis101.github.io/blantyreESBL/, and on a mirrored Zendo repository (https://doi.org/10.5281/zenodo.5554081).
