# Supplementary material for: Colonisation dynamics of extended spectrum beta-lactamase-producing Enterobacterales in the gut of Malawian adults
Source: Nat Microbiol. Author manuscript; Available in PMC 2022 Sep 30. (PMC9519460; doi:10.1038/s41564-022-01216-7)
Supplement: Extended Data Figure Legends [file EMS151721-supplement-Extended_Data_Figure_Legends.docx]

**Extended Data Figure 1: Species of bacteria isolated from stool.** Samples were labelled “Gram negative bacilli” if they could not be speciated using the API system.

**Extended Data Figure 2: Results of antimicrobial sensitivity testing (AST).** AST of cultured *E. coli* and *K. pneumoniae* sequence complex (KpSC) isolates using the disc diffusion method. A subset of isolates (442/473 E. coli and 167/203 KpSC) underwent AST.

**Extended Data Figure 3: Participant antimicrobial exposure and hospitalisation**. Stratified by study arm

**Extended Data Figure 4: Comparing ESBL-E carriage models**. Comparing a model of ESBL-E carriage that include a stepwise constant effect of hospitalisation and antimicrobial exposure (where the effect of covariates ceases when exposure ceases in Model 1) to a model that allows the effect of antimicrobial exposure to persist when exposure finishes, modelled as an exponential decay in Model 2. A-B: Parameter estimates from Model 1 expressed as natural logarithm of hazard ratio of gain or loss of ESBL-E for antimicrobial exposure [abx] and hospitalisation [hosp] (A) and mean time in the colonised or uncolonised state (B) with covariates set to 0 (i.e. no antimicrobials, not hospitalised). C-F: Parameter estimates from Model 2, with the same interpretation and the addition of the half-life (in days) of the decaying effect of antimicrobial exposure (F). G: Posterior parameter checks of two models showing actual prevalence of ESBL-E carriage stratified by study arm (dashed lines) with kernel density plots of predicted prevalence from fitted models, obtained by using all posterior parameter estimates (n=2000, discarding warmup iterations) to predict probability of ESBL-E from the actual data, and sampling from a binomial distribution using this probability. Model 1 underfits the antimicrobial-exposed arm of the study, which is improved by the addition of the prolonged effect of antimicrobials.

**Extended Data Figure 5: relative effects of hospitalisation and antimicrobial exposure**. A: Predicted probability from final fitted model of ESBL colonisation assuming 0.5 probability of colonisation at time t=0 and between 1-5 days of antimicrobial exposure (left) or hospitalisation (right). B: Estimated mean person-days of colonisation for the exposures in A out to 100 days (i.e. the area under the curves in A top 100 days). In all panels confidence intervals are 95% confidence intervals; the full model posterior was used to estimate the quantities of interest and confidence intervals constructed from quantiles of estimates.

**Extended Data Figure 6: Parameter estimates from models considering ceftriaxone and non-ceftriaxone antimicrobials.** (A-C) show parameter estimates from this model; (D-F) show original model. In each case, point shows posterior median and bar and whiskers show undertainty in parameter estimate by 50% and 95% credible interval, respectively, constructed from quantiles of the posterior estimates. Parameters are: a, loss parameters, b, gain parameters and g, decay parameter of effect of antimicrobials. [CRO] indicates that the parameter refers to ceftriaxone, [non-CRO] that the parameter refers to non-ceftriaxone antimicrobials, and [hosp] to hospitalisation.

**Extended Data Figure 7: Distribution of contig-clusters between and within genera**. (A) shows distribution of contig clusters by genus*.* (B-C) show contig-cluster presence (purple)-absence (grey) mapped back to core gene maximum likelihood phylogeny for *E. coli* (B) and *K. pneumoniae* subsp. *pneumoniae* (C)
