# Supplementary material for: Colonisation dynamics of extended spectrum beta-lactamase-producing Enterobacterales in the gut of Malawian adults
Source: Nat Microbiol. Author manuscript; Available in PMC 2022 Sep 30. (PMC9519460; doi:10.1038/s41564-022-01216-7)
Supplement: Supplementary Data [file EMS151721-supplement-Supplementary_Data.pdf]

# Supplementary material: Colonisation dynamics of extended spectrum beta-lactamase-producing Enterobacterales in the gut of Malawian adults


*1. Supplementary Figures and Tables* 2
1.1 Stool sample collection, exposures and ESBL-E results 2
Supplementary Figure 1: Distribution of stool sample collection dates 2
Supplementary Table 1: ESBL carriage prevalence. 3
Supplementary Table 2: Univariable and multivariable associations of baseline ESBL colonisation 4
Supplementary Table 3: Antimicrobial and hospital exposure stratified by arm 5
Supplementary Figure 2: Samples collected and proportion positive to day 10. 6
1.2 Modelling longitudinal ESBL-E carriage 6
Supplementary Table 4: Parameter estimates from final model. 6
Supplementary Figure 3: Pairs plot of posterior parameter estimates. 7
1.3 Genomic data: AMR genes and plasmid replicons 8
Supplementary Figure 4: Distribution of identified AMR genes in *E. coli* isolates. 8
Supplementary Figure 5: Distribution of identified AMR genes in *K. pmeumoniae* sequence complex isolates. 9
Supplementary Figure 6: Distribution of plasmid incompatibility groups. 10
1.4 PopPUNK and contig-cluster analysis 11
Supplementary Figure 7: *Klebsiella pneumoniae* sequence complex popPUNK clusters 11
Supplementary Figure 8: *E. coli* popPUNK clusters. 11
Supplementary Figure 9: PopPUNK clusters mapped to core gene phylogeny. 12
Supplementary Figure 10: Descriptive statistics of contig-clusters. 13
Supplementary Figure 11: Stability of contig clusters to varying cd-hit parameters. 14
Supplementary Figure 12: Multiple sequence alignment of contig cluster CTXM27.1. 15
Supplementary Figure 13: Multiple sequence alignment of contig cluster CTXM15.123. 16
Supplementary Figure 14: Multiple sequence alignment of contig cluster CTXM15.113. 17
Supplementary Figure 15: Multiple sequence alignment of contig cluster CTXM15.66 18
Supplementary Figure 16: Multiple sequence alignment of contig cluster CTXM15.10. 19
Supplementary Figure 17: Multiple sequence alignment of contig cluster CTXM15.69 20
Supplementary Figure 18: Multiple sequence alignment of contig cluster CTXM15.41 21
Supplementary Figure 19: Multiple sequence alignment of contig cluster CTXM15.61 22
Supplementary Figure 20: Multiple sequence alignment of contig cluster CTXM15.29. 23
Supplementary Figure 21: Multiple sequence alignment of contig cluster SHV12.1 24
1.7 Sensitivity analyses 25
Supplementary Figure 22: Effect of varying SNP cluster definition of within-participant correlation. 25
Supplementary Figure 23: Associations of popPUNK cluster (A) and contig cluster (B) to healthcare associated isolates. 26
Supplementary Figure 24: Effect of varying SNP cluster definition on between-participant clustering (*E. coli*) 27
Supplementary Figure 25: Effect of varying SNP cluster definition on between-participant clustering (K. pneumoniae sequence complex). 28
Supplementary Figure 26: Effect of varying SNP cluster definition on cluster epidemiology. 29

# 1. Supplementary Figures and Tables

## 1.1 Stool sample collection, exposures and ESBL-E results

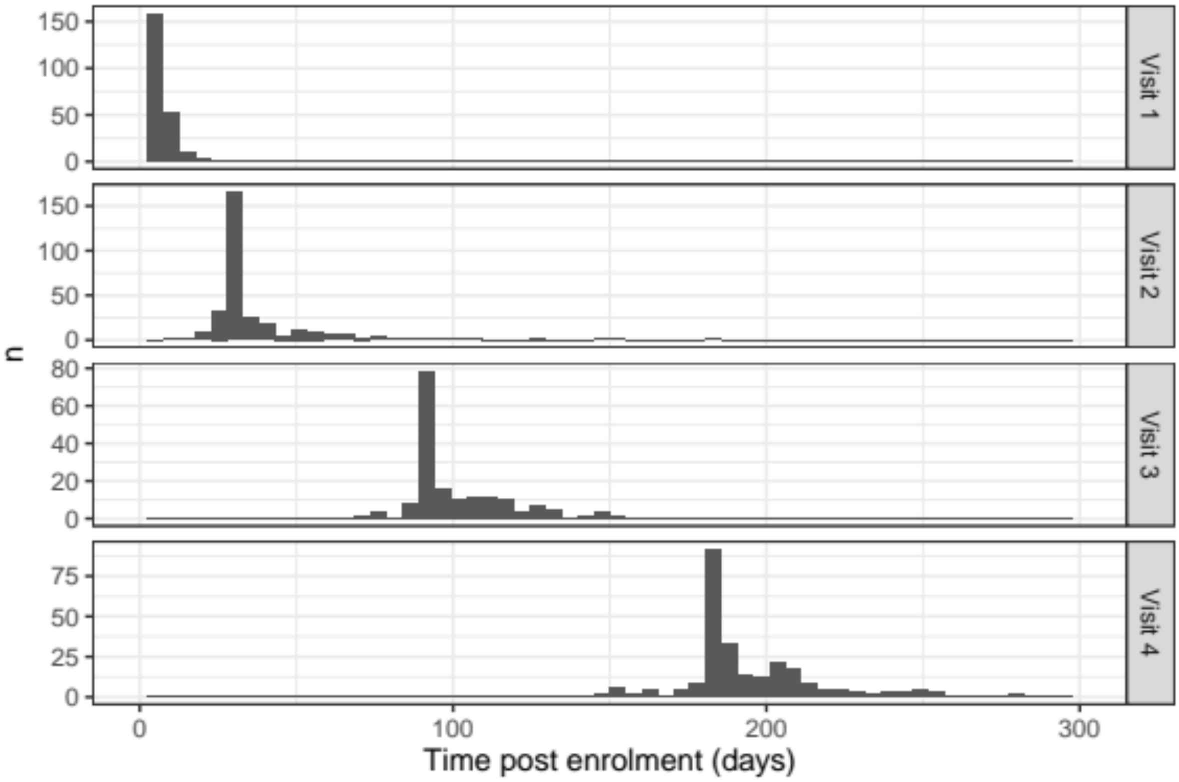


Supplementary Figure 1: Distribution of stool sample collection dates

Supplementary Table 1: ESBL carriage prevalence.

| | Sepsis | | | | Inpatient | | | | Community | | | |
|---|---|---|---|---|---|---|---|---|---|---|---|---|
| **Visit** | **n** | **ESBL** | ***E. coli*** | **KpSC** | **n** | **ESBL** | ***E. coli*** | **KpSC** | **n** | **ESBL** | ***E. coli*** | **KpSC** |
| Baseline | 222 | 109 (49%) | 98 (44%) | 34 (15%) | 99 | 41 (41%) | 35 (35%) | 10 (10%) | 99 | 28 (28%) | 25 (25%) | 6 (6%) |
| Day 7 | 162 | 127 (78%) | 122 (75%) | 45 (28%) | 63 | 32 (51%) | 27 (43%) | 8 (13%) | - | - | - | - |
| Day 28 | 148 | 106 (72%) | 97 (66%) | 46 (31%) | 71 | 37 (52%) | 30 (42%) | 16 (23%) | 92 | 29 (32%) | 23 (25%) | 9 (10%) |
| Day 90 | 126 | 71 (56%) | 62 (49%) | 24 (19%) | 60 | 29 (48%) | 25 (42%) | 4 (7%) | - | - | - | - |
| Day 180 | 127 | 61 (48%) | 55 (43%) | 24 (19%) | 65 | 29 (45%) | 23 (35%) | 5 (8%) | 82 | 23 (28%) | 18 (22%) | 2 (2%) |

ESBL = Extended-spectrum beta-lactamase, KpSC = *K. pneumoniae* complex.

Supplementary Table 2: Univariable and multivariable associations of baseline ESBL colonisation

| | Univariable | | Multivariable | |
|---|---|---|---|---|
| **Variable** | **OR (95% CI)** | **p-value** | **aOR (95% CI)** | **p-value** |
| **Study Arm** | | | | |
| Inpatient (vs community) | 1.79 (1.00-3.26) | 0.054 | 1.68 (0.81-3.53) | 0.164 |
| Sepsis (vs community) | **2.45 (1.48-4.12)** | **<0.001** | 1.08 (0.54-2.22) | 0.822 |
| **Demographics** | | | | |
| Age (per year) | 1.00 (0.99-1.02) | 0.709 | 1.00 (0.98-1.02) | 0.922 |
| Male sex (vs female) | 1.23 (0.84-1.82) | 0.287 | 1.44 (0.94-2.21) | 0.098 |
| **HIV status** | | | | |
| HIV+ (vs HIV-) | **1.68 (1.09-2.59)** | **0.018** | 1.21 (0.48-2.99) | 0.679 |
| HIV unknown (vs HIV-) | 0.71 (0.40-1.24) | 0.229 | 1.08 (0.54-2.16) | 0.820 |
| ART (vs none) | **1.99 (1.32-3.00)** | **0.001** | 1.07 (0.35-3.23) | 0.905 |
| CPT (vs none) | **2.46 (1.58-3.86)** | **<0.001** | 2.34 (1.00-5.66) | 0.053 |
| **Healthcare exposure** | | | | |
| Current TB treatment | 1.02 (0.33-2.99) | 0.971 | 0.51 (0.13-1.80) | 0.300 |
| Antibiotics*† | **1.81 (1.05-3.16)** | **0.034** | 1.24 (0.64-2.41) | 0.528 |
| Hospitalisation† | **7.87 (2.57-34.22)** | **0.001** | **6.64 (1.98-30.75)** | **0.005** |
| **Household** | | | | |
| Unprotected water source | 2.43 (0.96-6.64) | 0.068 | **2.96 (1.07-8.75)** | **0.040** |
| Treat water (vs not) | 1.16 (0.50-2.66) | 0.725 | 0.95 (0.37-2.37) | 0.913 |
| Flushing toilet (vs. not) | 0.72 (0.29-1.80) | 0.481 | 1.11 (0.41-3.04) | 0.842 |
| Adults (per 1) | 1.14 (0.99-1.31) | 0.064 | **1.20 (1.03-1.40)** | **0.024** |
| Children (per 1) | 1.00 (0.87-1.14) | 0.979 | 0.98 (0.84-1.13) | 0.747 |
| Keep animals (vs. not) | 1.33 (0.88-2.03) | 0.176 | 1.15 (0.72-1.84) | 0.552 |
| **Season** | | | | |
| Rainy season‡ (vs. dry) | **2.05 (1.38-3.06)** | **<0.001** | **2.21 (1.40-3.50)** | **<0.001** |

CPT = Co-trimoxazole preventative therapy, ART = antiretroviral therapy, TB = tuberculosis. Entries in bold are those for which 95% confidence intervals do not cross 1. P-values are from logistic regression using *glm* in R v4.0.2 testing the hypothesis that there is no correlation with the outcome.

* Antibiotics includes TB therapy but excludes CPT. † Hospitalisation and antimicrobial exposure are self reported by participants within 28 days prior to enrolment. ‡ Rainy season defined as November-April

Supplementary Table 3: Antimicrobial and hospital exposure stratified by arm

| | Number exposed | | | Exposure (person-days) | | | Median (IQR) exposure length (days) | | |
|---|---|---|---|---|---|---|---|---|---|
| **Exposure** | **Sepsis** | **Inpatient** | **Community** | **Sepsis** | **Inpatient** | **Community** | **Sepsis** | **Inpatient** | **Community** |
| Total at risk | 225 | 100 | 100 | 33797 | 14336 | 21983 | 183 (63-203) | 182 (97-187) | 200 (185-219) |
| **Exposures** | | | | | | | | | |
| Hospitalised | 225 | 100 | 1 | 1727 | 500 | 1 | 5 (2-10) | 2 (2-7) | 1 (1-1) |
| Ceftriaxone | 183 | 7 | 0 | 997 | 26 | 0 | 5 (3-7) | 3 (2-4) | - |
| Co-trimoxazole | 110 | 6 | 7 | 14447 | 549 | 1388 | 180 (27-190) | 86 (6-177) | 190 (183-206) |
| Ciprofloxacin | 61 | 2 | 0 | 398 | 12 | 0 | 7 (5-7) | 6 (6-6) | - |
| TB therapy | 52 | 2 | 0 | 6843 | 291 | 0 | 178 (58-180) | 146 (133-158) | - |
| Amoxicillin | 38 | 3 | 1 | 235 | 21 | 5 | 7 (5-7) | 5 (5-8) | 5 (5-5) |
| Fluconazole | 27 | 0 | 0 | 118 | 0 | 0 | 3 (2-5) | - | - |
| Metronidazole | 24 | 2 | 0 | 148 | 10 | 0 | 6 (2-7) | 5 (5-5) | - |
| Artesunate | 11 | 0 | 0 | 25 | 0 | 0 | 2 (2-3) | - | - |
| Co-amoxiclav | 10 | 2 | 0 | 40 | 12 | 0 | 5 (2-5) | 6 (6-6) | - |
| LA | 7 | 0 | 0 | 19 | 0 | 0 | 3 (2-3) | - | - |
| Doxycycline | 7 | 0 | 0 | 34 | 0 | 0 | 3 (2-6) | - | - |
| Erythromycin | 5 | 0 | 0 | 38 | 0 | 0 | 7 (5-11) | - | - |
| Gentamicin | 4 | 0 | 0 | 15 | 0 | 0 | 4 (3-5) | - | - |
| Streptomycin | 2 | 0 | 0 | 16 | 0 | 0 | 8 (7-9) | - | - |
| Penicillin | 2 | 0 | 0 | 5 | 0 | 0 | 2 (2-3) | - | - |
| Flucloxacillin | 2 | 0 | 0 | 5 | 0 | 0 | 2 (2-3) | - | - |
| Azithromycin | 2 | 2 | 0 | 7 | 12 | 0 | 4 (3-4) | 6 (6-6) | - |
| Amphotericin | 2 | 0 | 0 | 8 | 0 | 0 | 4 (4-4) | - | - |
| Aciclovir | 2 | 0 | 0 | 47 | 0 | 0 | 24 (16-31) | - | - |
| Quinine | 1 | 0 | 0 | 1 | 0 | 0 | 1 (1-1) | - | - |

TB = tuberculosis, LA = Lumefantrine artemether. Median exposure length includes only those exposed. Total at risk shows the total number of participants and participant-days of follow up included in the study.

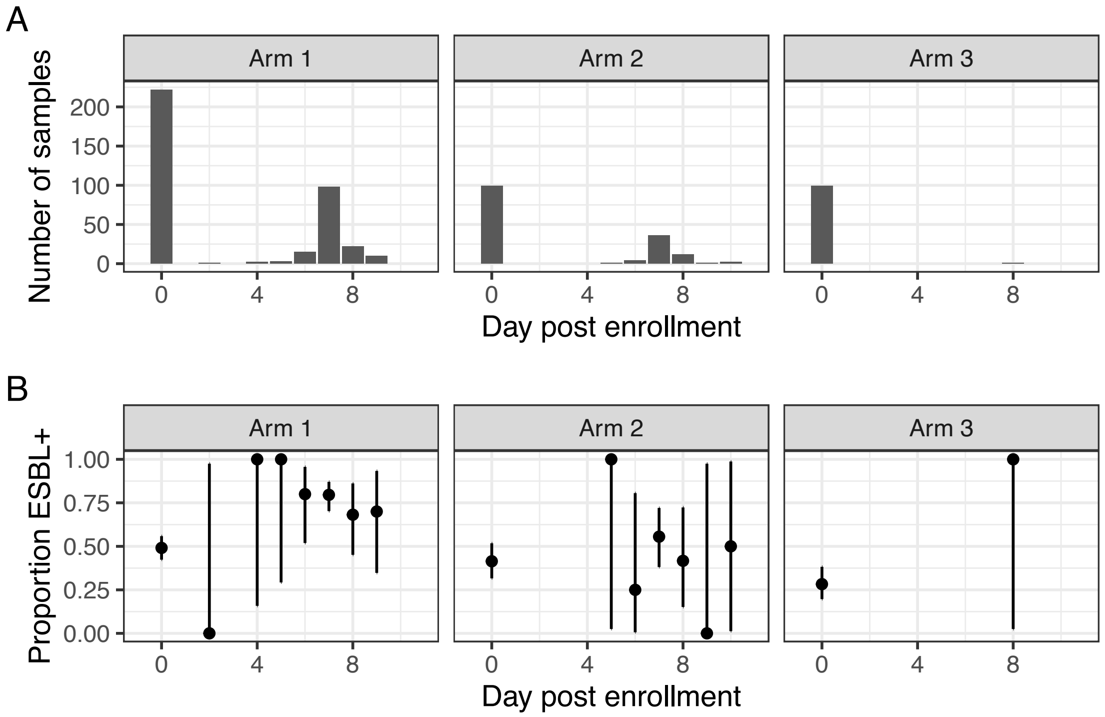


Supplementary Figure 2: Samples collected and proportion positive to day 10. Number of samples collected per day over first ten days of study (A) and proportion of samples each day from which at least one ESBL-E was isolated with exact binomial confidence intervals(B).

## 1.2 Modelling longitudinal ESBL-E carriage

Supplementary Table 4: Parameter estimates from final model. In the mathematical notation used in the methods section hazard ratios are the exponential of the parameters $\alpha$ and $\beta$ in the model; half life is equal to $\gamma$log2); mean time in state assumes all other covariates are equal to zero and is then the reciprocal of $\lambda$ or $\mu$.

| Variable | Value |
|---|---|
| **Effect of Antibacterials** | |
| Hazard ratio ESBL-E Loss | 0.16 (0.05-0.58) |
| Hazard ratio ESBL-E Gain | 0.57 (0.16-2.25) |
| Half life of effect (days) | 43.7 (15.4-97.7) |
| **Effect of Hospitalisation** | |
| Hazard ratio ESBL-E Loss | 10.01 (1.24-52.34) |
| Hazard ratio ESBL-E Gain | 27.82 (3.60-143.18) |
| **Mean time in state** | |
| Colonised (days) | 9.7 (4.2-25.1) |
| Uncolonised (days) | 5.8 (2.5-14.3) |

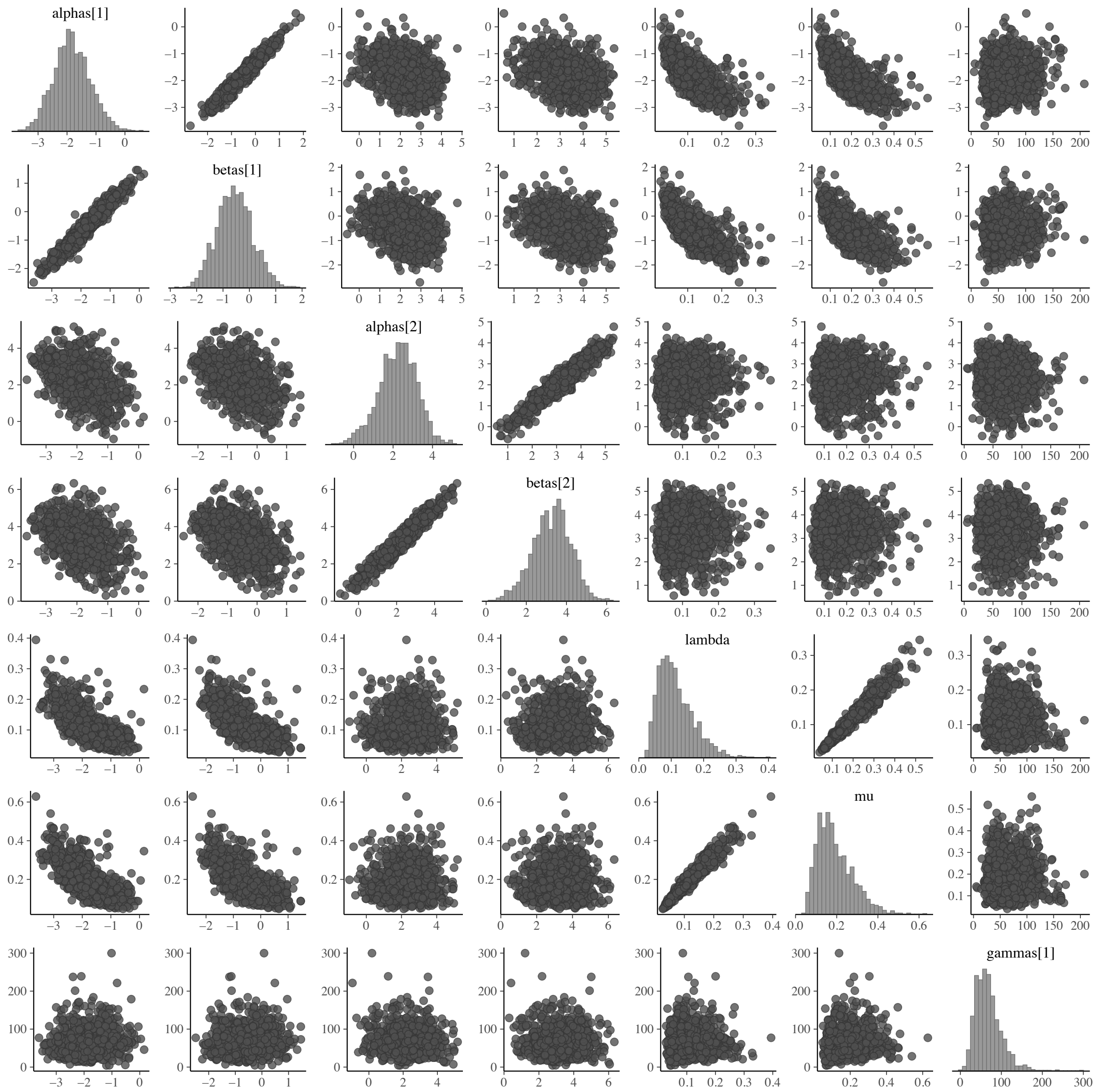


Supplementary Figure 3: Pairs plot of posterior parameter estimates. This shows non-identifiability of gain and loss parameters manifesting as strong correlation. Alpha[1] and beta[1] are log hazard ratio of hospitalisation and alpha[2] and beta[2] of antimicrobial exposure. Lamda and mu are baseline instantaneous ESBL-E loss and gain and gamma is the scaled half-life of the effect of antimicrobial exposure

## 1.3 Genomic data: AMR genes and plasmid replicons

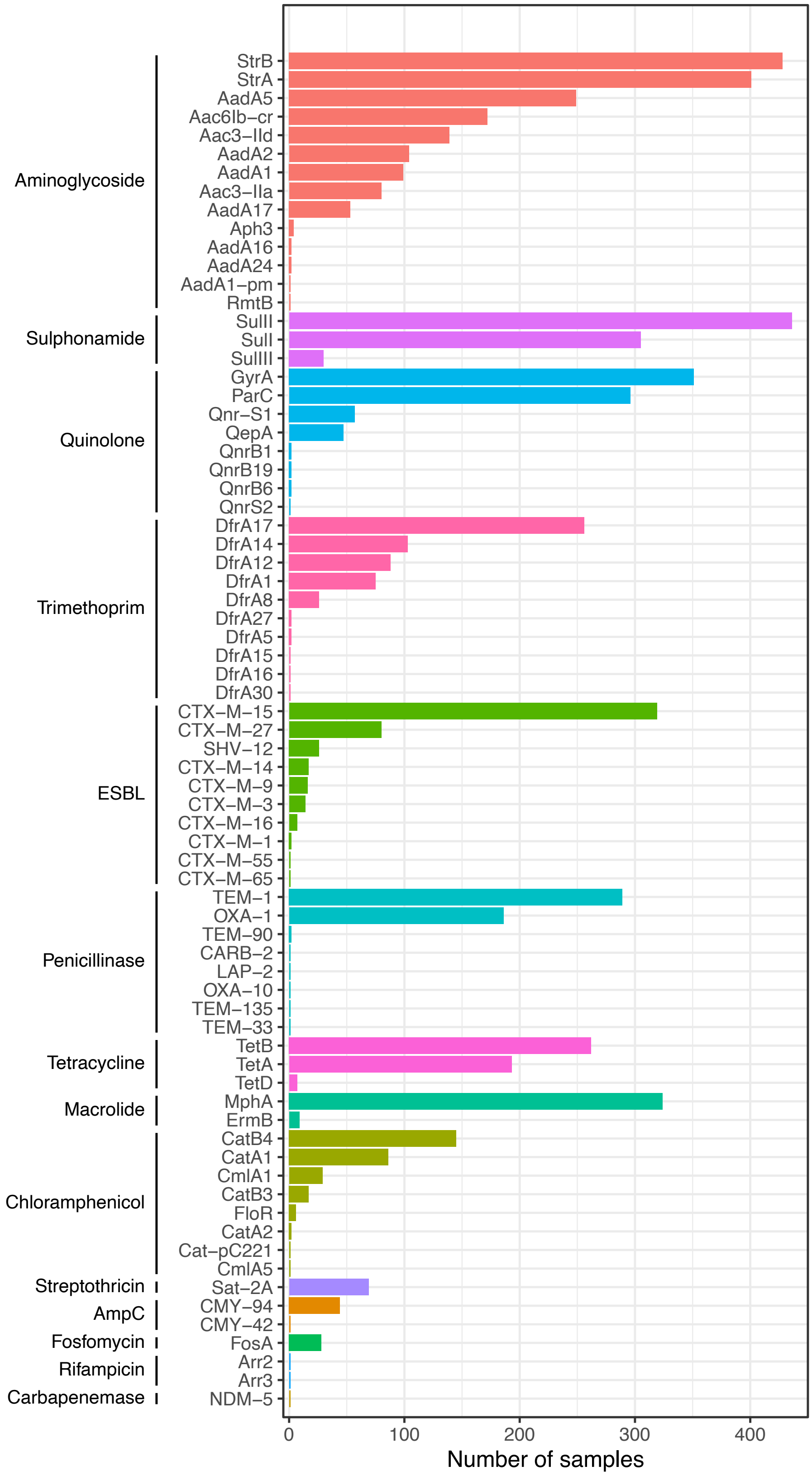


Supplementary Figure 4: Distribution of identified AMR genes in *E. coli* isolates.

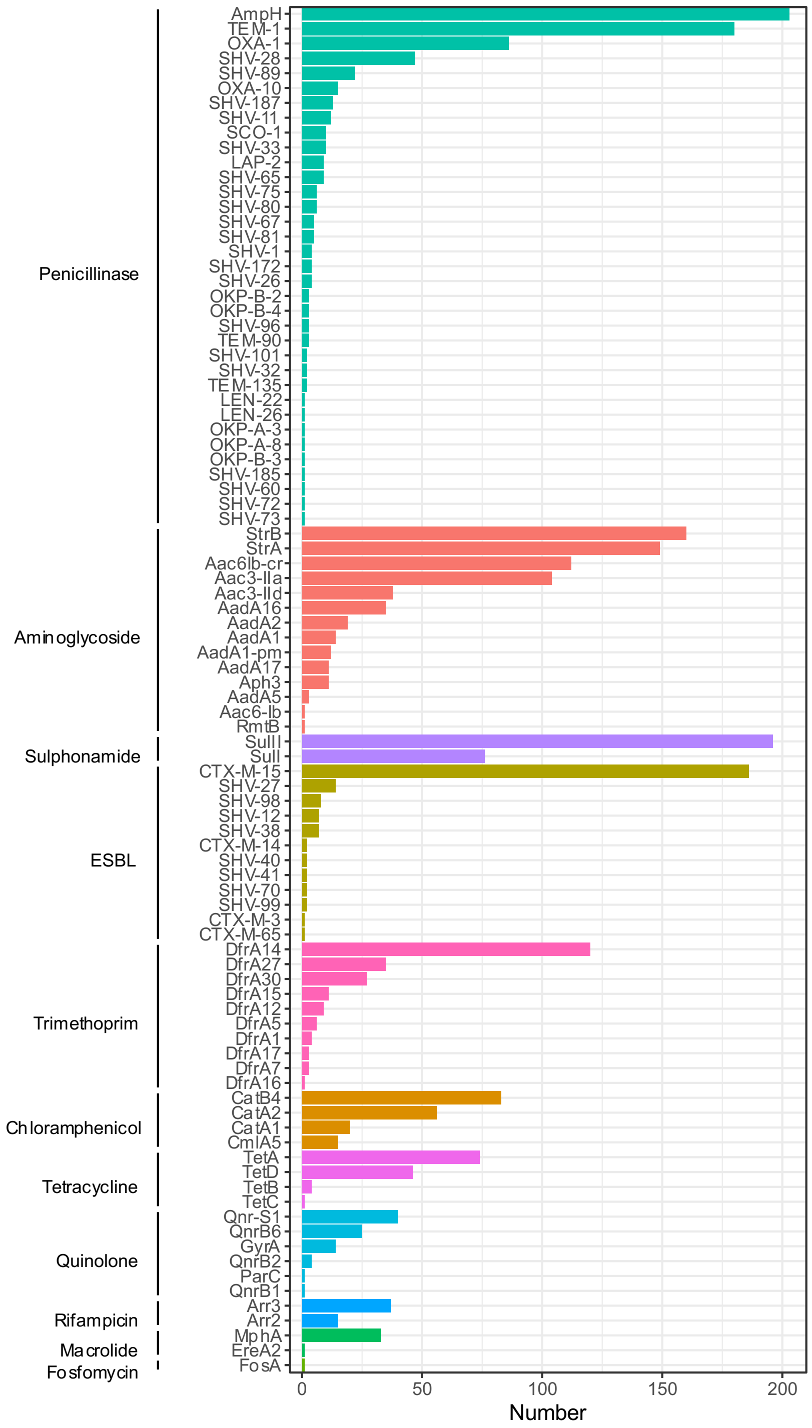


Supplementary Figure 5: Distribution of identified AMR genes in *K. pmeumoniae* sequence complex isolates.

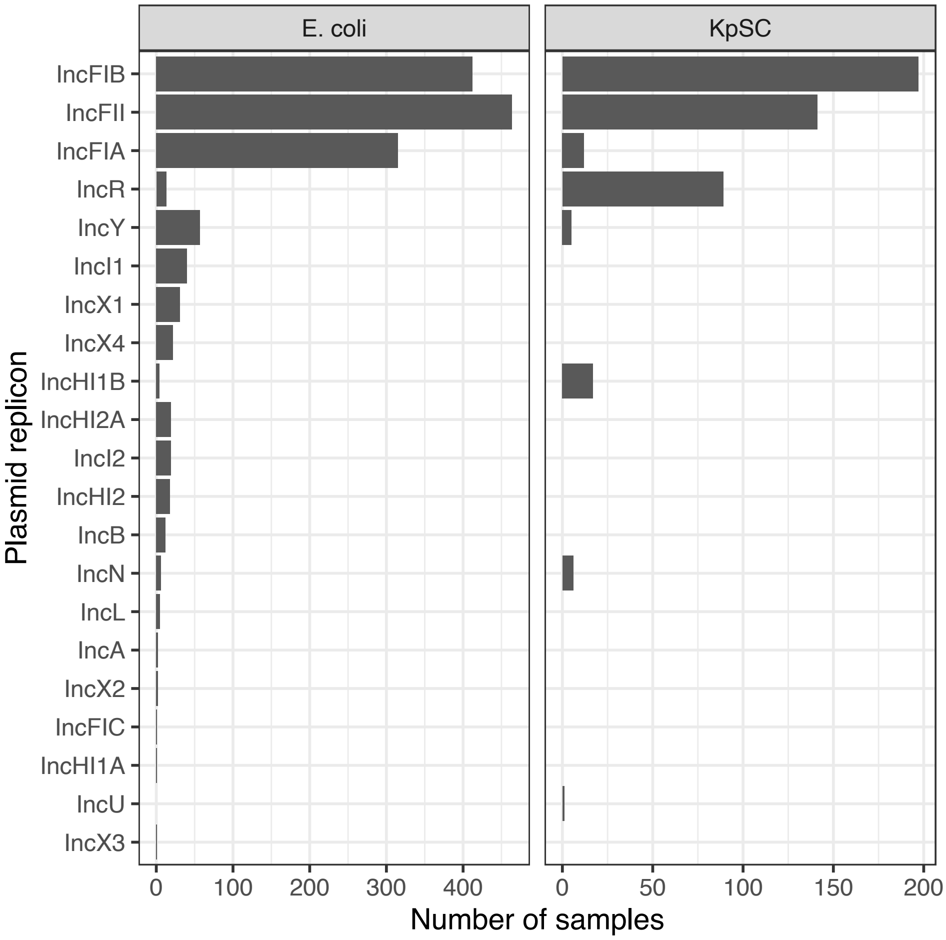


Supplementary Figure 6: Distribution of plasmid incompatibility groups. KpSC = *K. pneumoniae* sequence complex.

### 1.4 PopPUNK and contig-cluster analysis

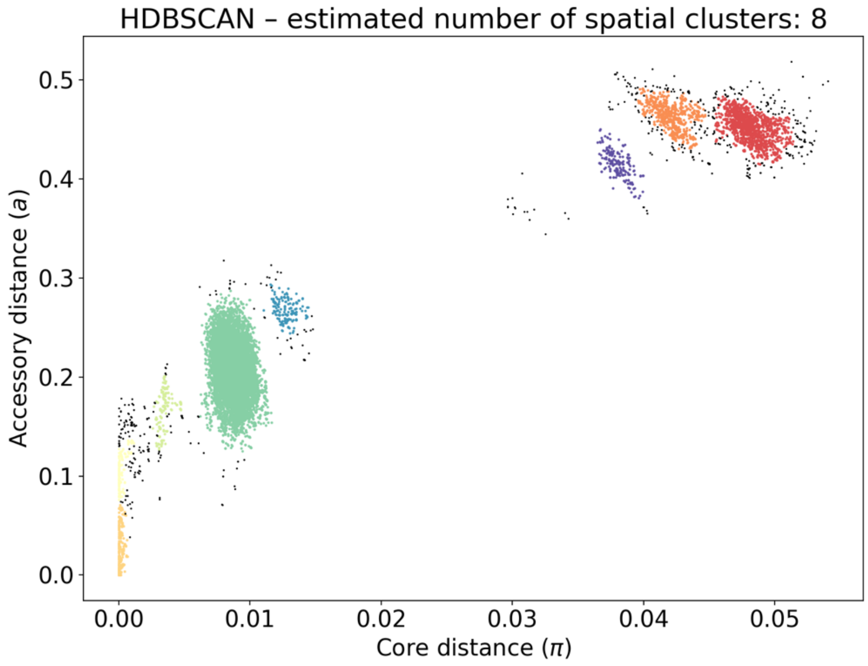


Supplementary Figure 7: *Klebsiella pneumoniae* sequence complex popPUNK clusters. Plot shows core and accessory genome distance (as defined by the popPUNK algorithm) with clusters indicated by colour.

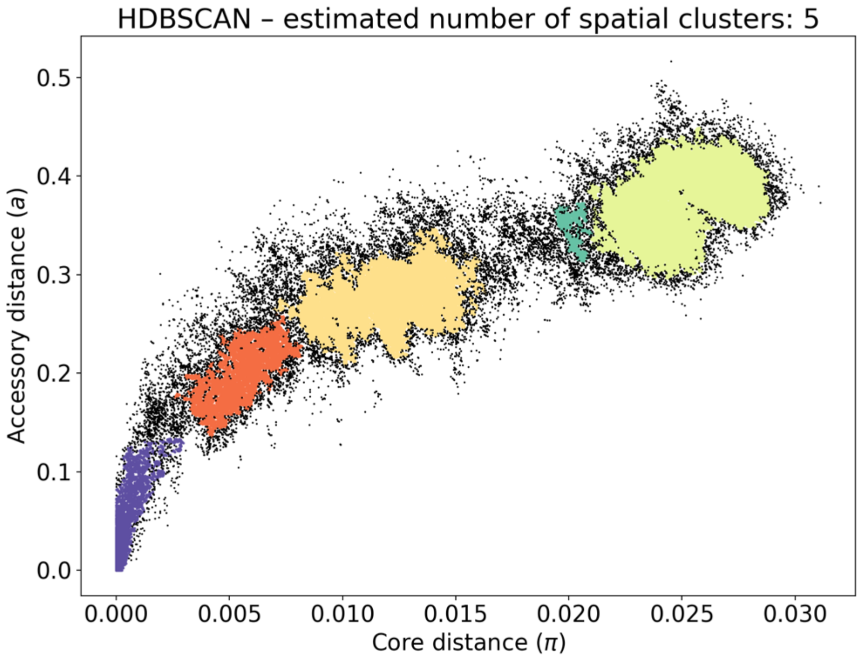


Supplementary Figure 8: *E. coli* popPUNK clusters. Plot shows core and accessory genome distance (as defined by the popPUNK algorithm) with clusters indicated by colour.

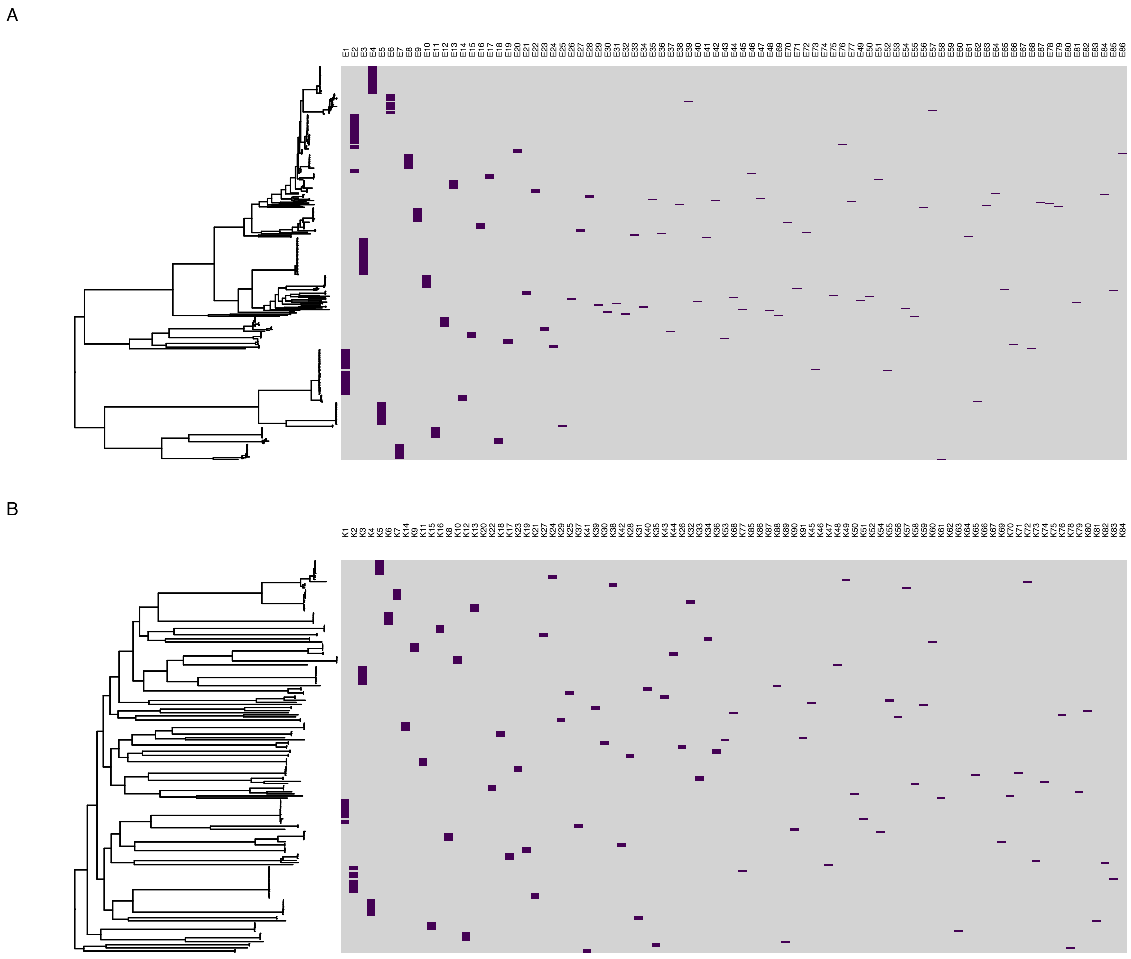


Supplementary Figure 9: PopPUNK clusters mapped to core gene phylogeny. popPUNK sequence clusters across the core-gene maximum-likelihood phylogeny for *E. coli* (A) and *K. pneumoniae* subsp. *pneumoniae* (B). popPUNK sequence cluster name shown at top of heatmap and purple indicates cluster membership. The sequence clusters correspond well to lineages inferred from the phylogeny.

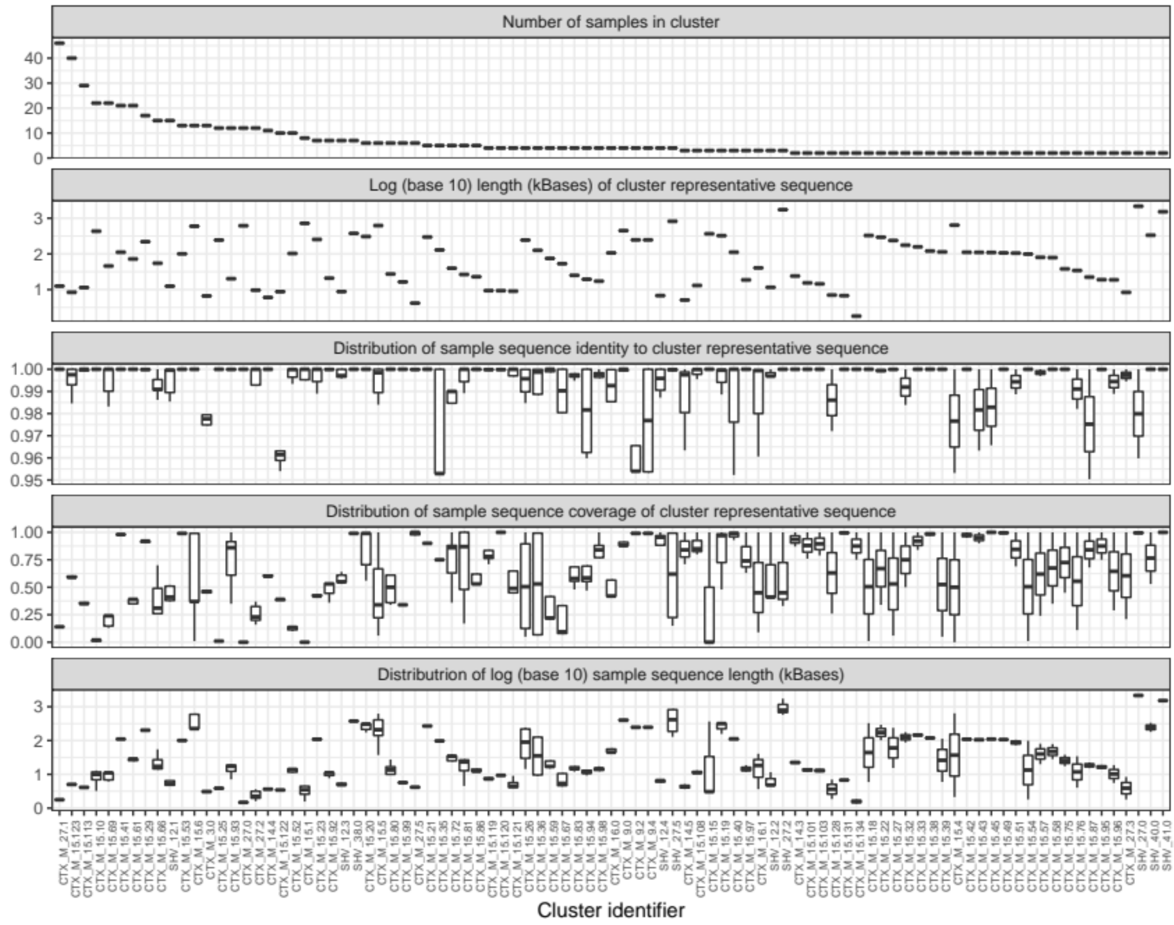


Supplementary Figure 10: Descriptive statistics of contig-clusters.

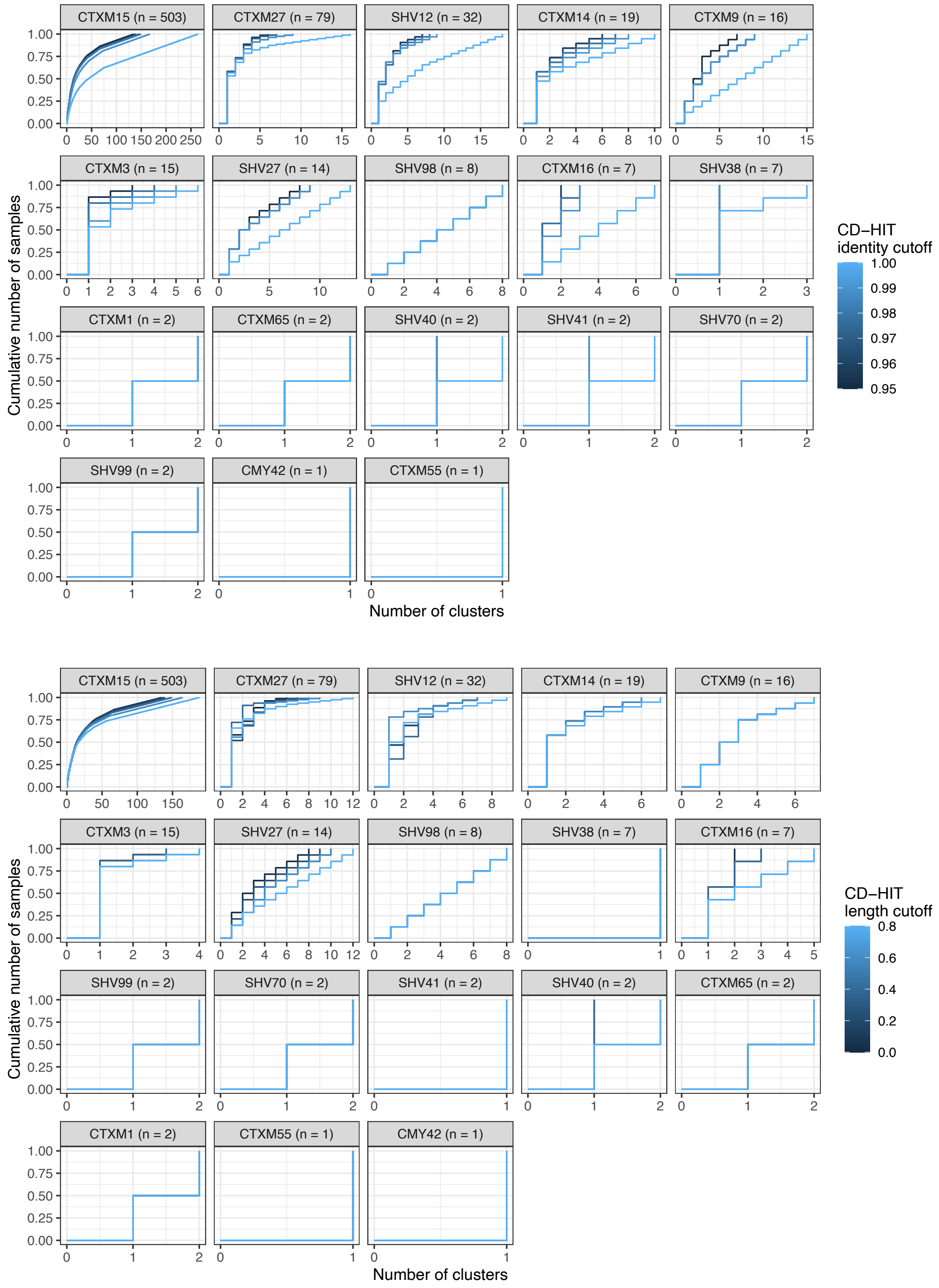


Supplementary Figure 11: Stability of contig clusters to varying cd-hit parameters.

Cumulative number of samples which are members of a contig cluster (y axis) as the number of contig clusters increases (x axis) where clusters are arranged from largest to smallest with varying cd-hit sequence identity cutoff for cluster membership (top) and length cutoff for cluster membership (i.e. smaller contigs must be at least length cutoff to be included in a cluster, bottom). Each panel shows a different ESBL gene ordered from most common (top left) to least (bottom right) with number of samples per gene shown in panel heading.

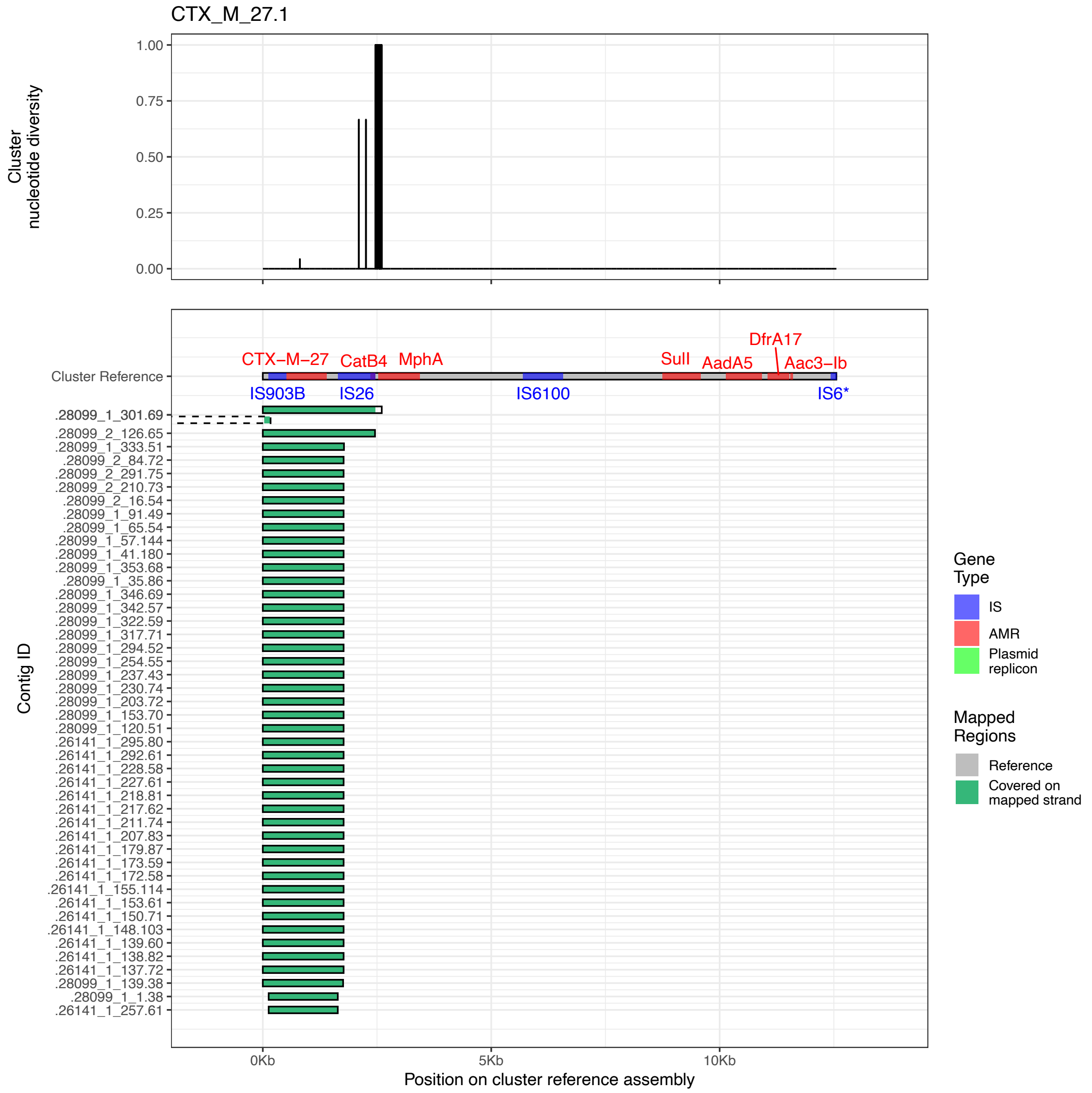


Supplementary Figure 12: Multiple sequence alignment of contig cluster CTXM27.1. AMR genes/insertion sequences identified to family level only are shown with an asterisk. Secondary alignments are shown with dashed lines

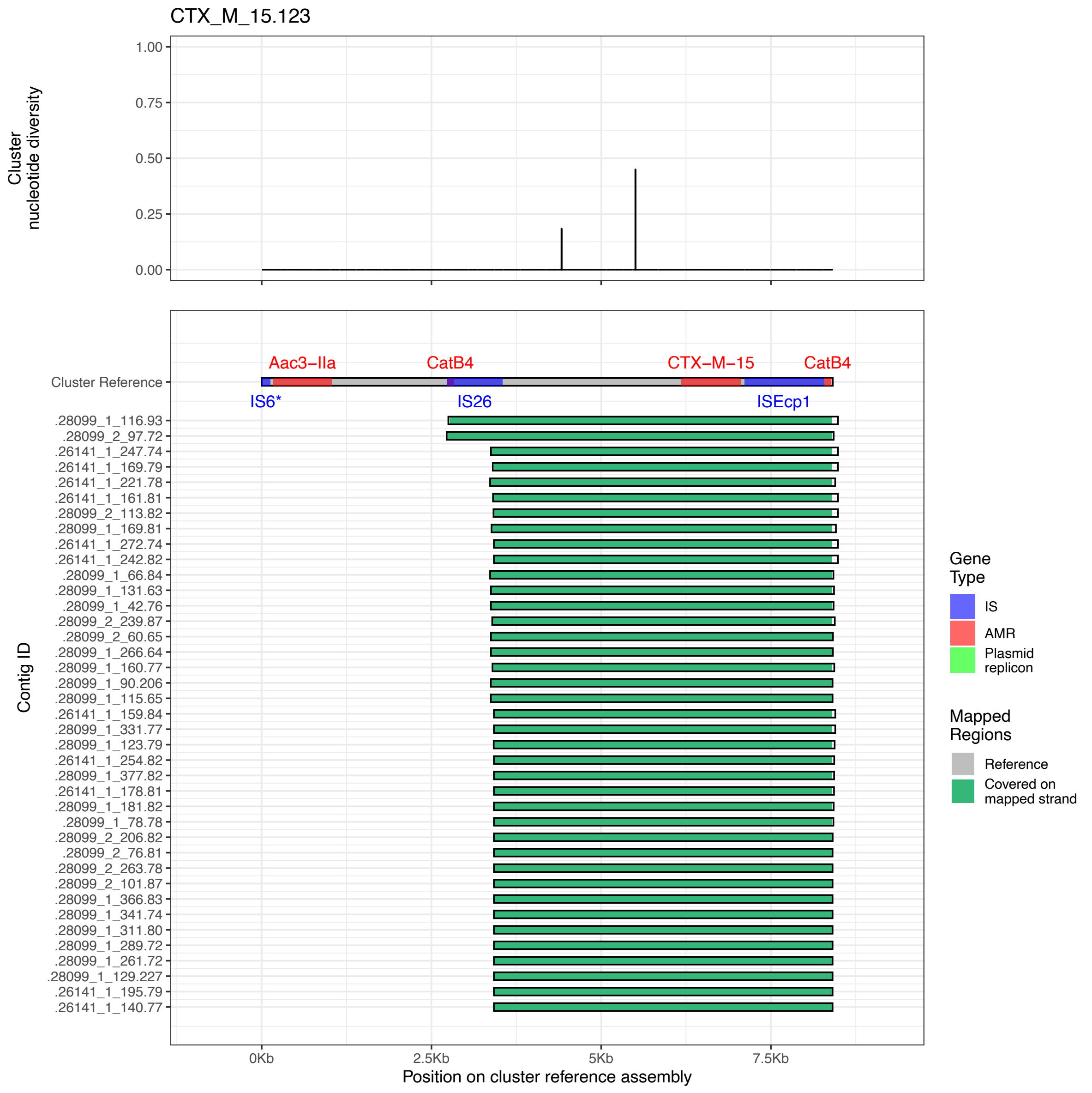


Supplementary Figure 13: Multiple sequence alignment of contig cluster CTXM15.123. AMR genes/insertion sequences identified to family level only are shown with an asterisk.

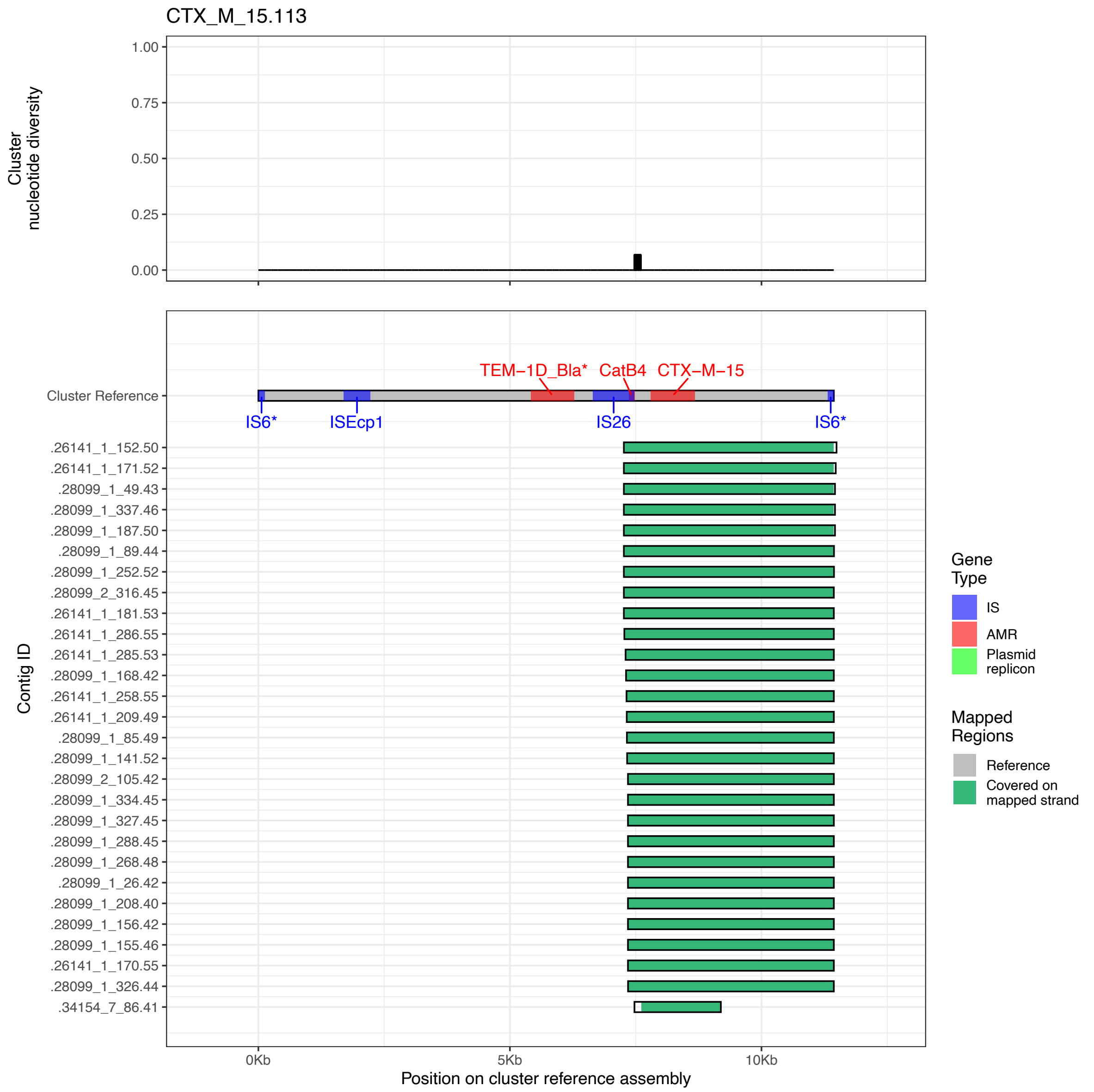


Supplementary Figure 14: Multiple sequence alignment of contig cluster CTXM15.113. AMR genes/insertion sequences identified to family level only are shown with an asterisk.

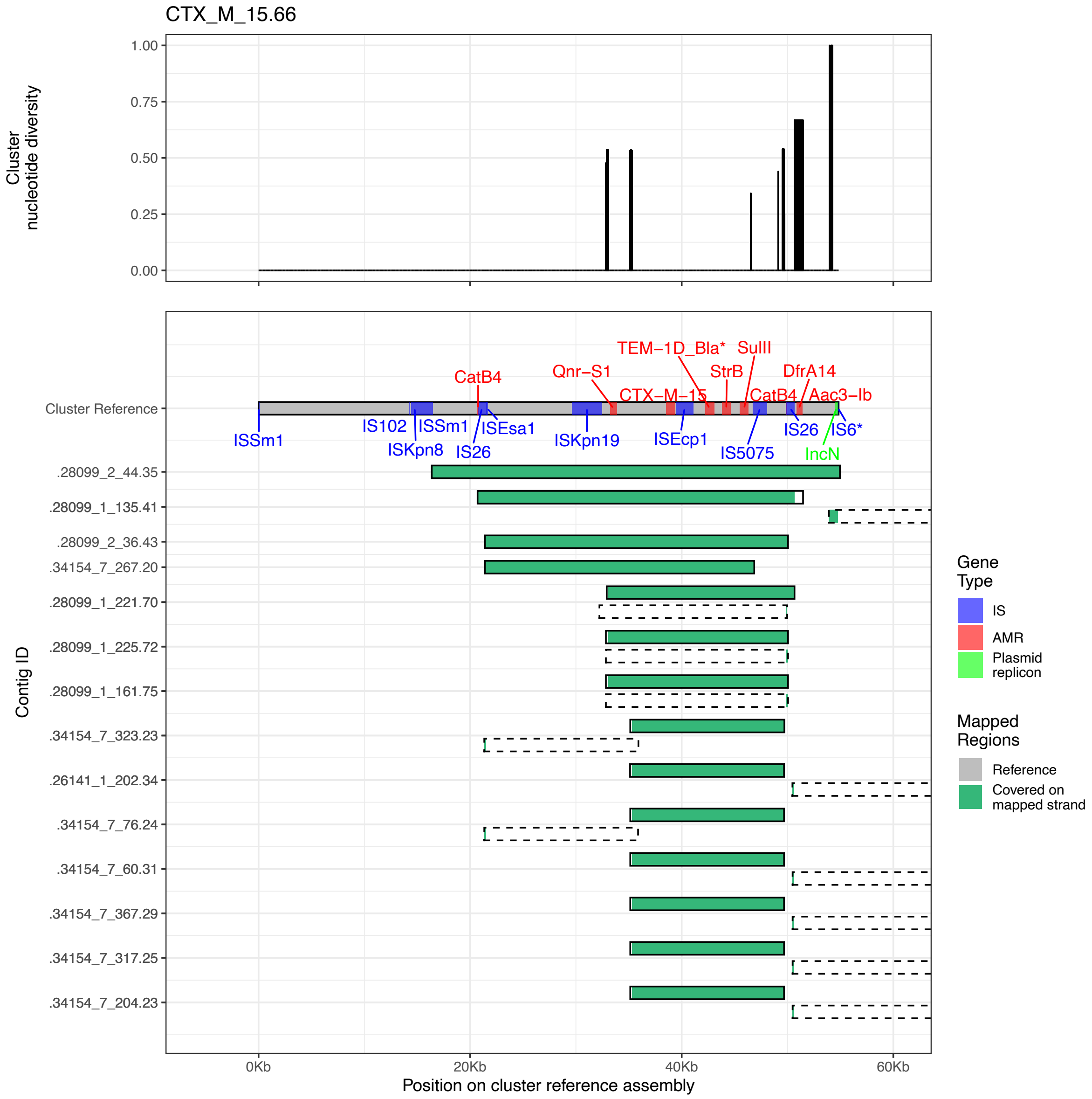


Supplementary Figure 15: Multiple sequence alignment of contig cluster CTXM15.66. AMR genes/insertion sequences identified to family level only are shown with an asterisk. Secondary alignments shown with dashed lines.

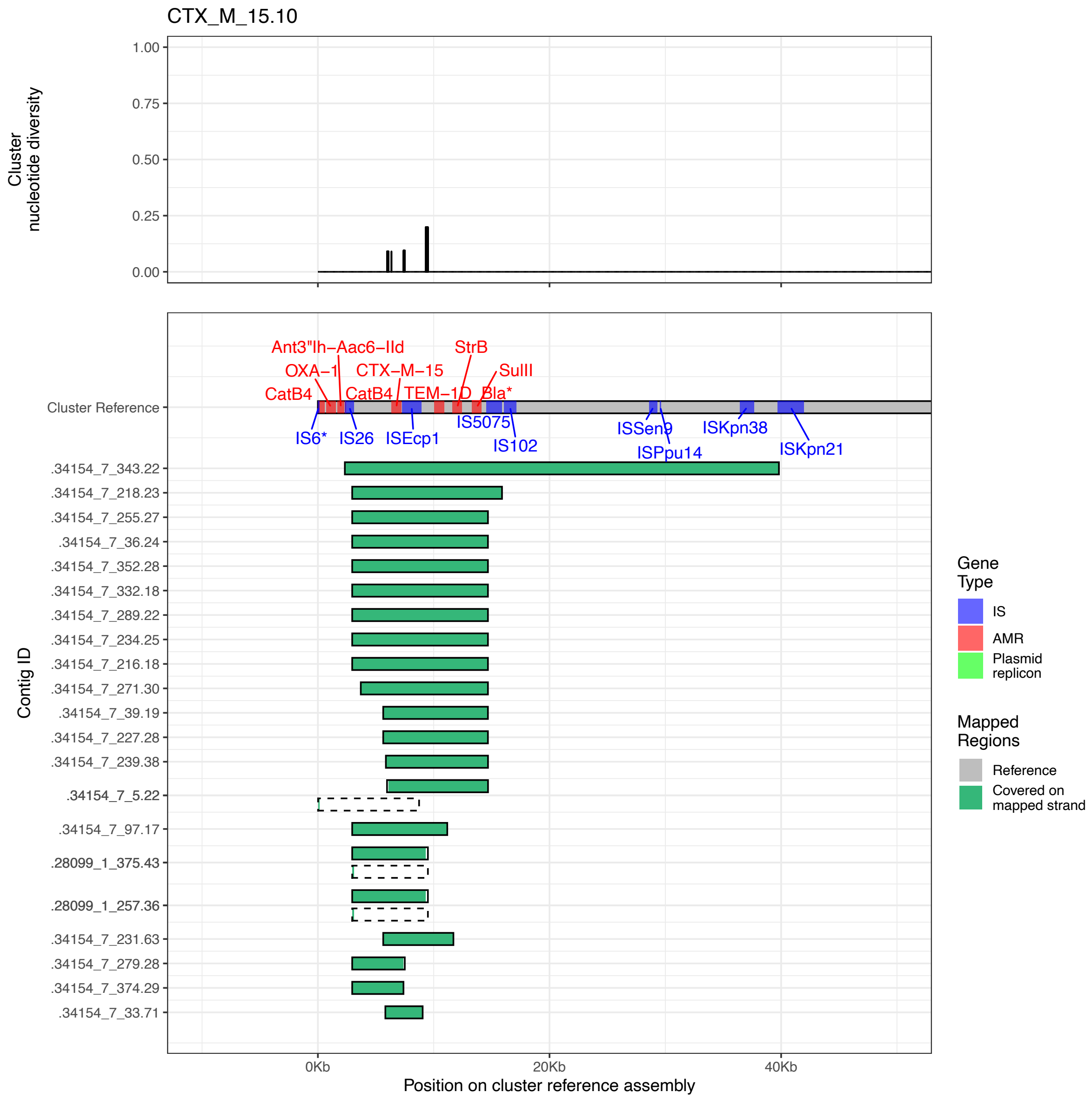


Supplementary Figure 16: Multiple sequence alignment of contig cluster CTXM15.10. AMR genes/insertion sequences identified to family level only are shown with an asterisk. Secondary alignments shown with dashed lines.

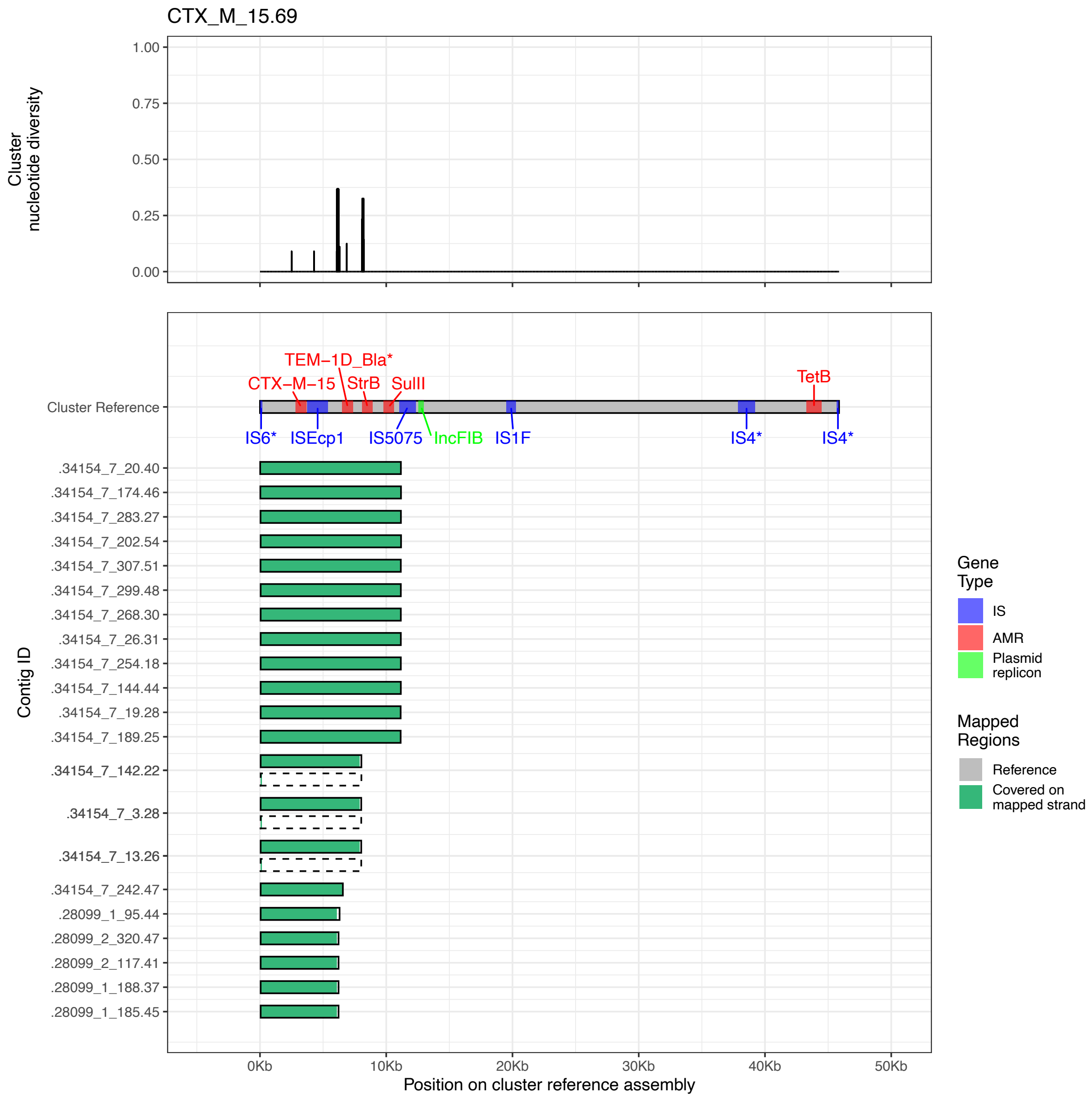


Supplementary Figure 17: Multiple sequence alignment of contig cluster CTXM15.69. AMR genes/insertion sequences identified to family level only are shown with an asterisk. Secondary alignments shown with dashed lines.

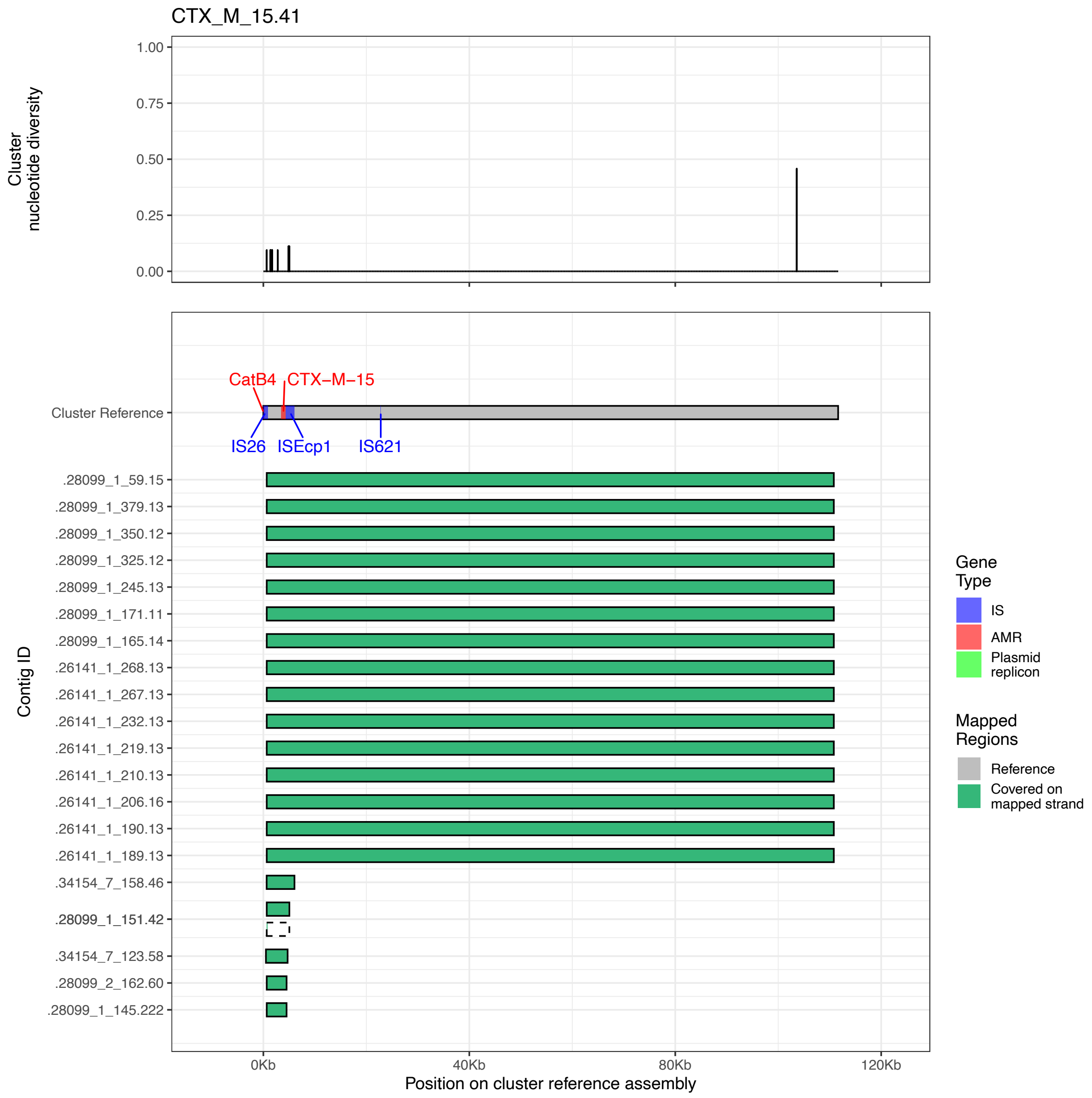


Supplementary Figure 18: Multiple sequence alignment of contig cluster CTXM15.41. AMR genes/insertion sequences identified to family level only are shown with an asterisk. Secondary alignments shown with dashed lines.

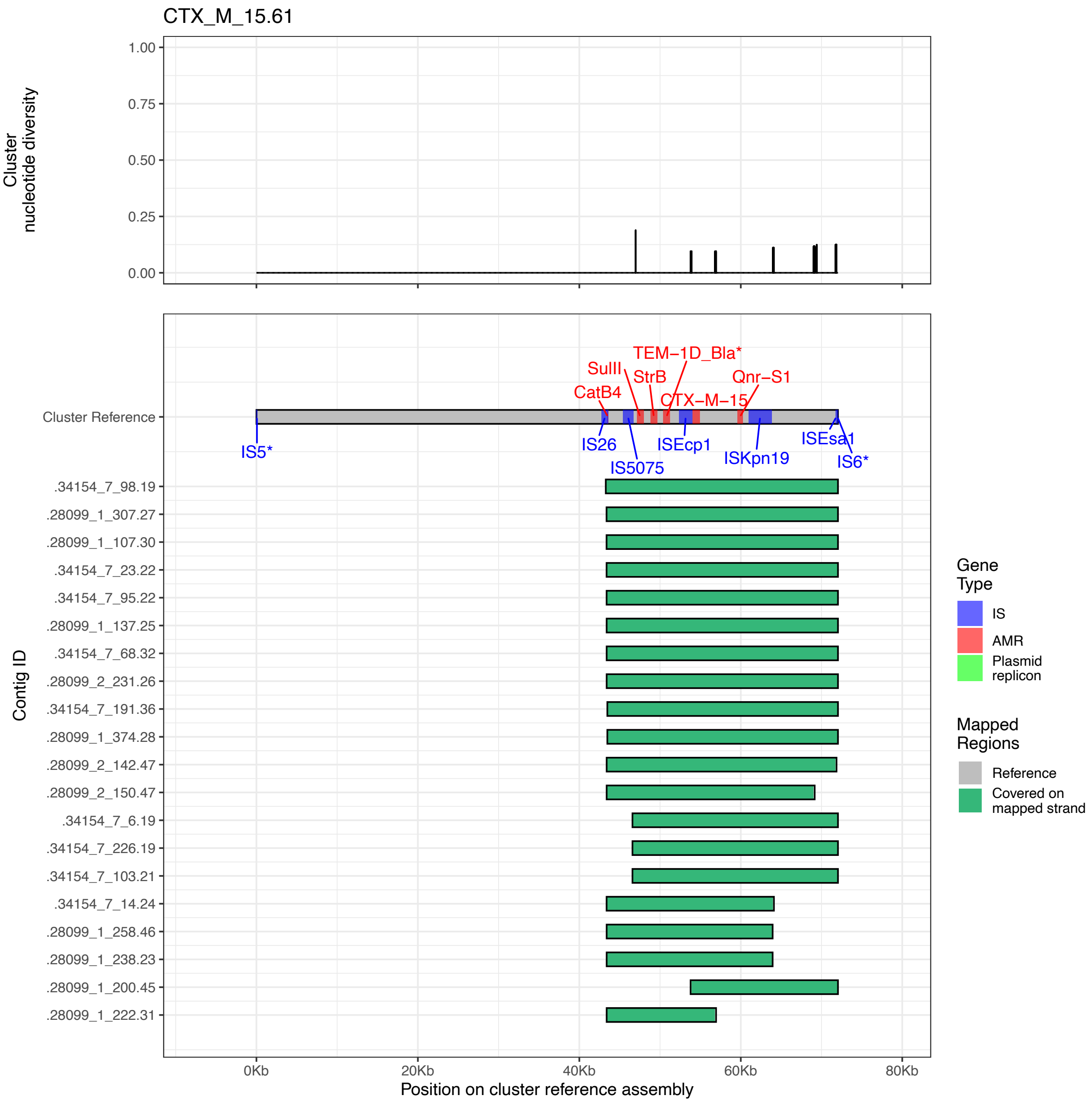


Supplementary Figure 19: Multiple sequence alignment of contig cluster CTXM15.61. AMR genes/insertion sequences identified to family level only are shown with an asterisk. Secondary alignments shown with dashed lines.

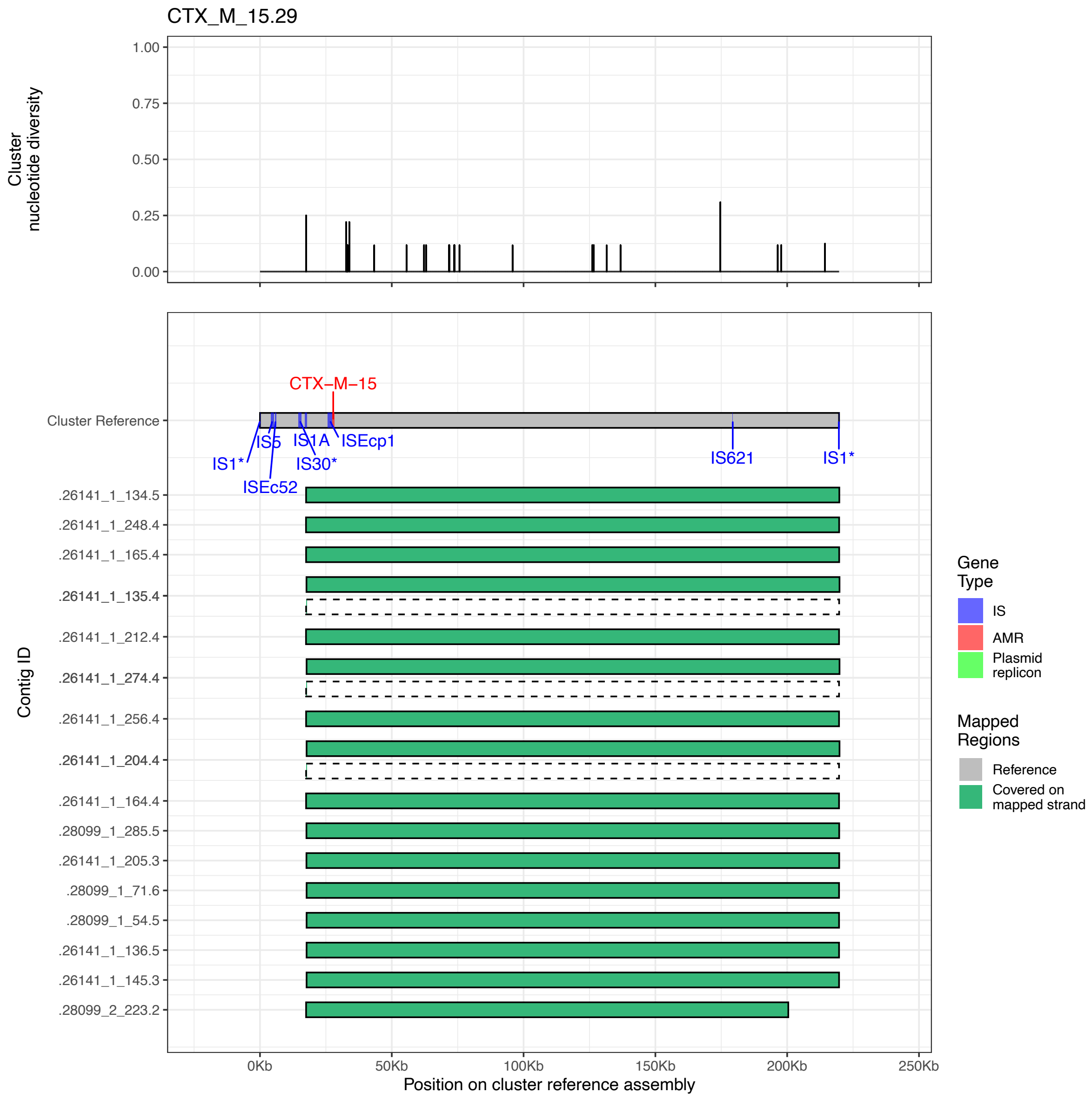


Supplementary Figure 20: Multiple sequence alignment of contig cluster CTXM15.29. AMR genes/insertion sequences identified to family level only are shown with an asterisk. Secondary alignments shown with dashed lines.

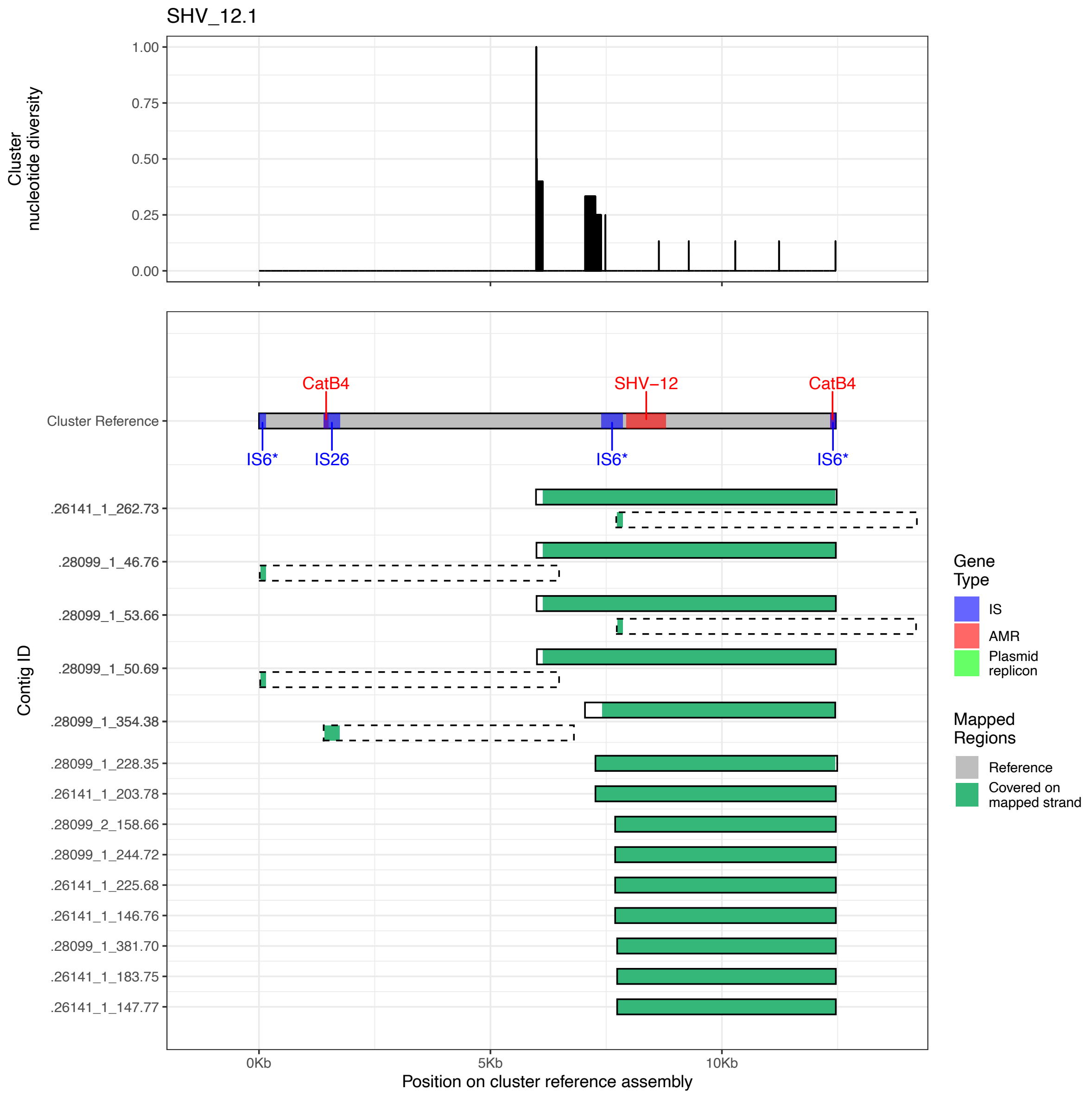


Supplementary Figure 21: Multiple sequence alignment of contig cluster SHV12.1. AMR genes/insertion sequences identified to family level only are shown with an asterisk. Secondary alignments shown with dashed lines.

## 1.7 Sensitivity analyses

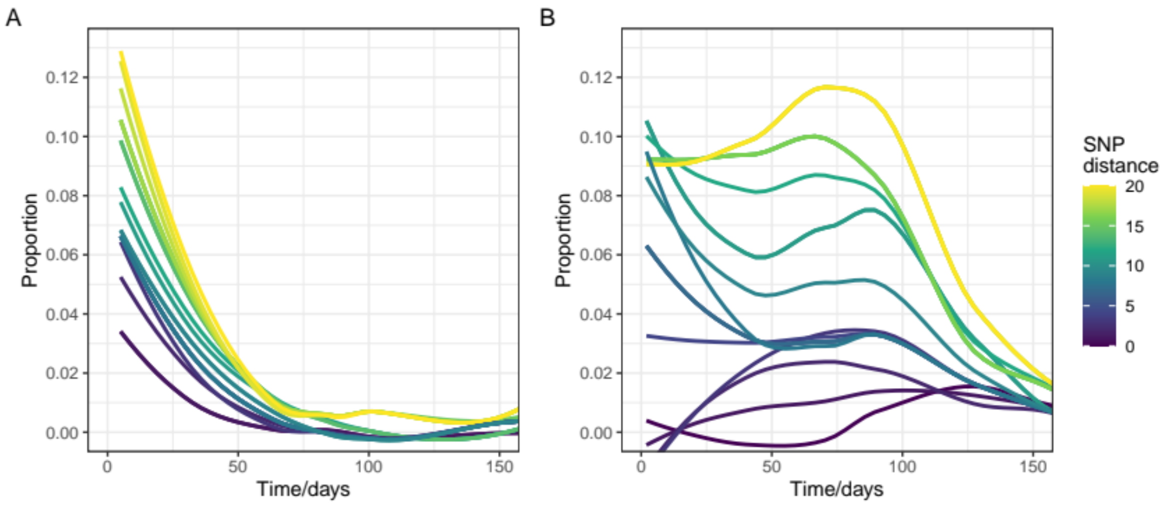


Supplementary Figure 22: Effect of varying SNP cluster definition of within-participant correlation. Sensitivity analysis examining effect of changing definition of SNP-cluster from 0 to 20 SNPs. Plots show the proportion of participants who are colonised with *E. coli* (A) or *K. pneumoniae* (B) at time t = 0 who are colonised with a bacterium from the same SNP cluster (and therefore possibly the same clone) and time *t* days later

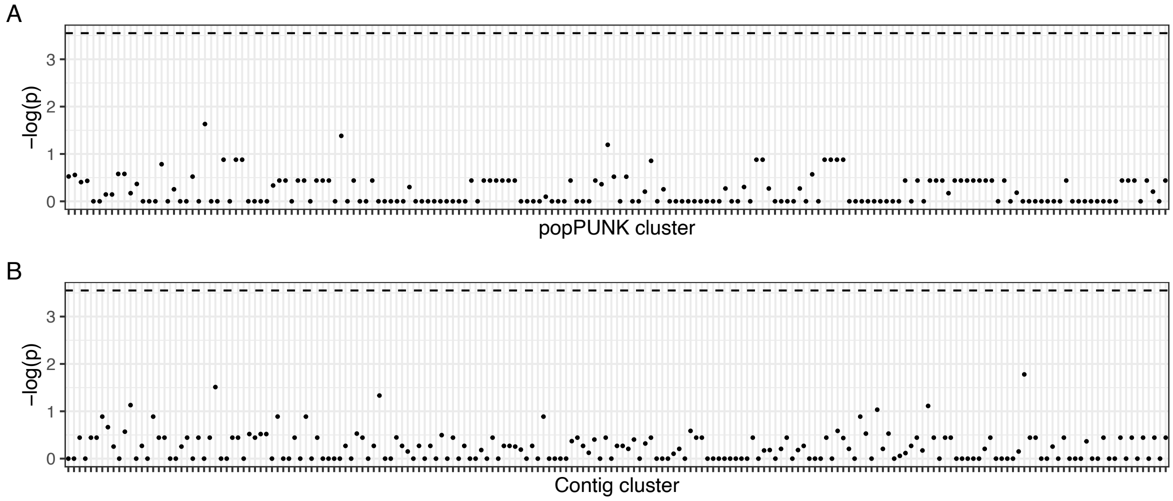


Supplementary Figure 23: Associations of popPUNK cluster (A) and contig cluster (B) to healthcare associated isolates. Healthcare associated here is defined as either in-hospital or recent discharge (within 120 days of admission to hospital). Dotted horizontal line represents Bonferroni-corrected p = 0.05 significance threshold.

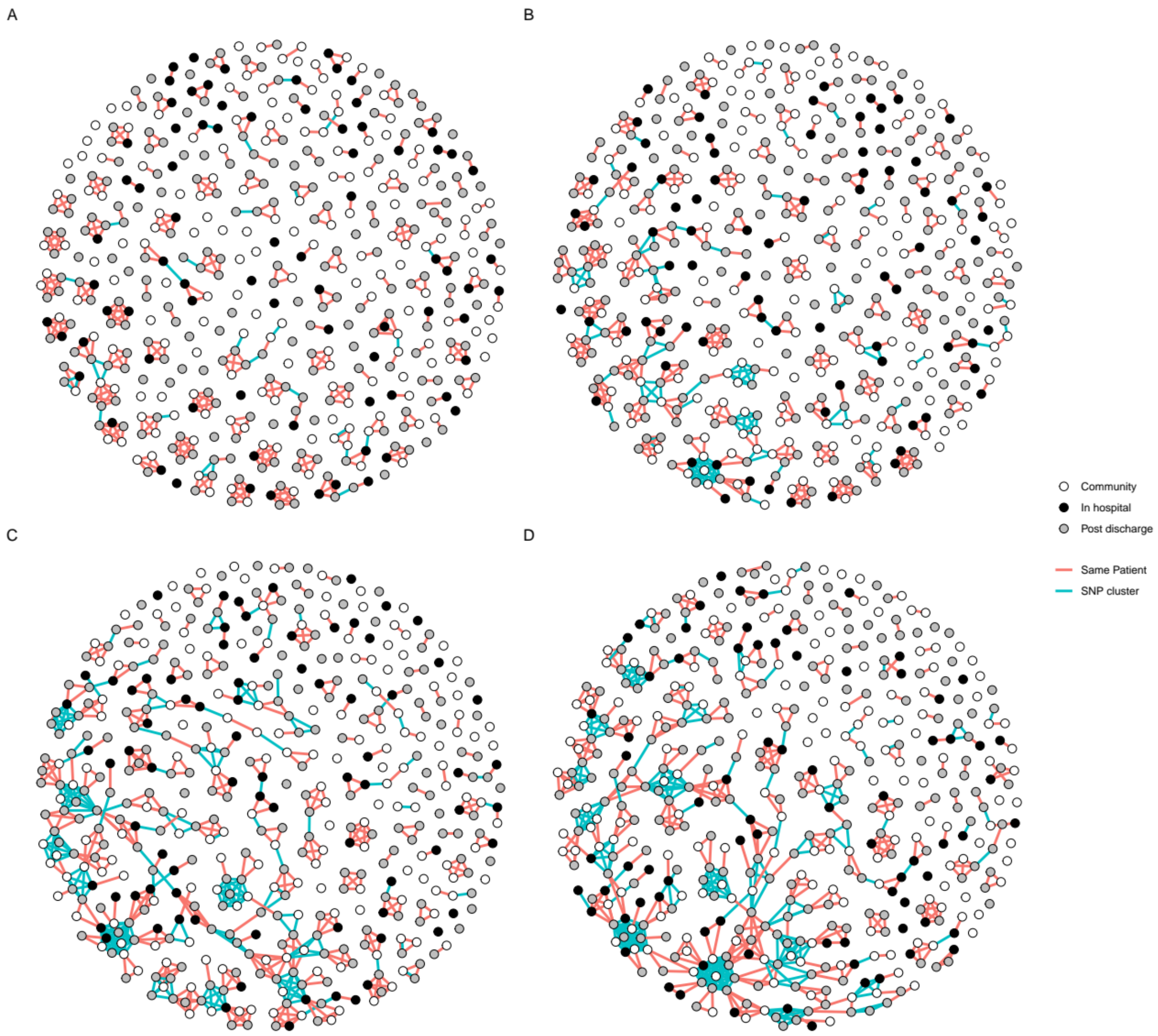


Supplementary Figure 24: Effect of varying SNP cluster definition on between-participant clustering (*E. coli*). Sensitivity analysis showing changing network plot for *E. coli* as the definition of SNP cluster is changed from 0 (A) to 3 (B), 7 (C) or 10 (D). Points are samples, coloured by place of isolation (in-hospital [black], community [white], or up to 120 days post-discharge [grey]). Red lines link samples that are within a single participant. Blue lines link samples that are differ by 0 (A), 3 (B), 7 (C), (D) or fewer SNPs.

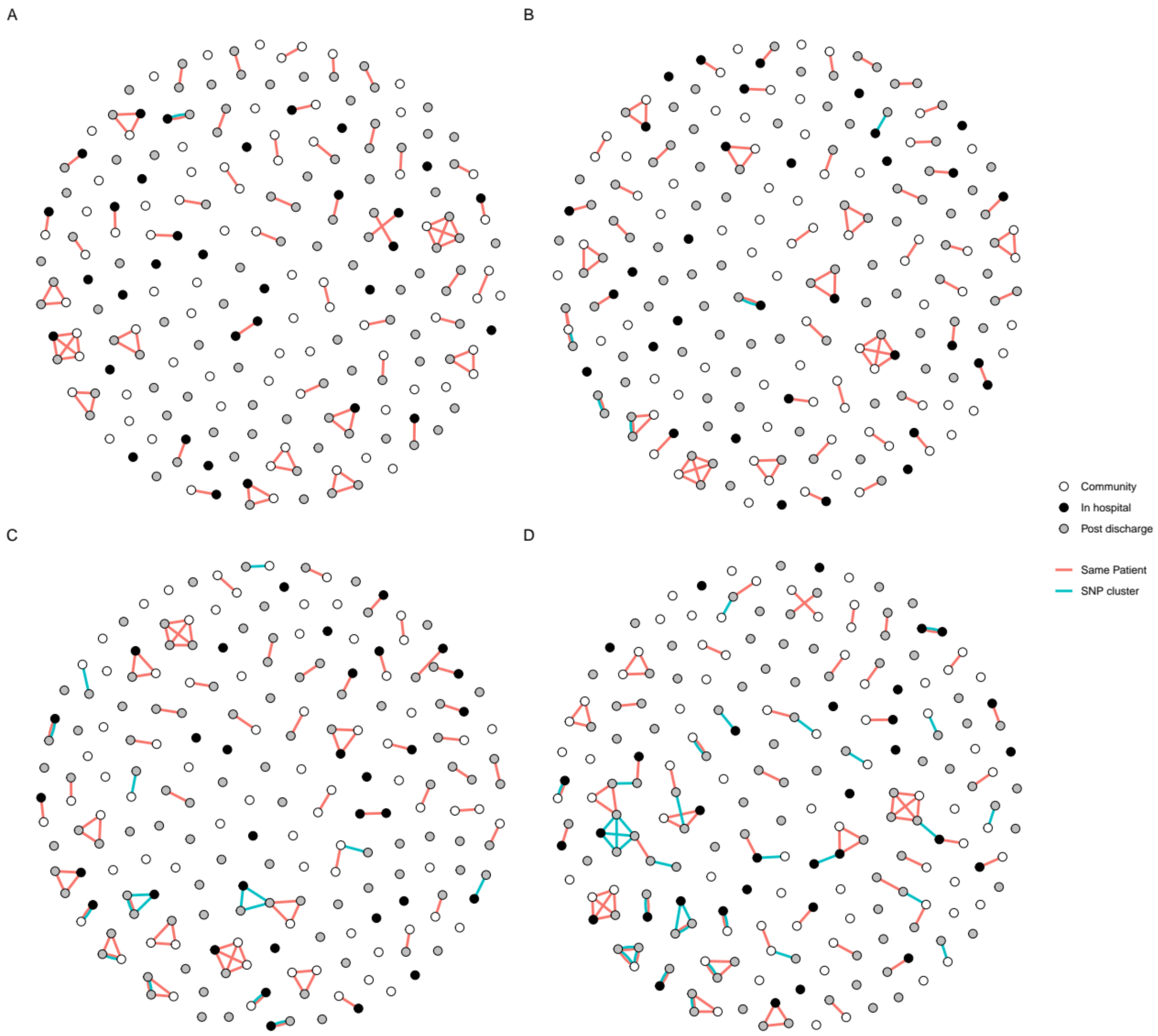


Supplementary Figure 25: Effect of varying SNP cluster definition on between-participant clustering (K. pneumoniae sequence complex). Sensitivity analysis showing changing network plot for *K. pneumoniae* as the definition of SNP cluster is changed from 0 (A) to 3 (B), 7 (C) or 10 (D). Points are samples, coloured by place of isolation (in-hospital [black], community [white], or up to 120 days post-discharge [grey]). Red lines link samples that are within a single participant. Blue lines link samples that are differ by 0 (A), 3 (B), 7 (C), 10 (D) or fewer SNPs.

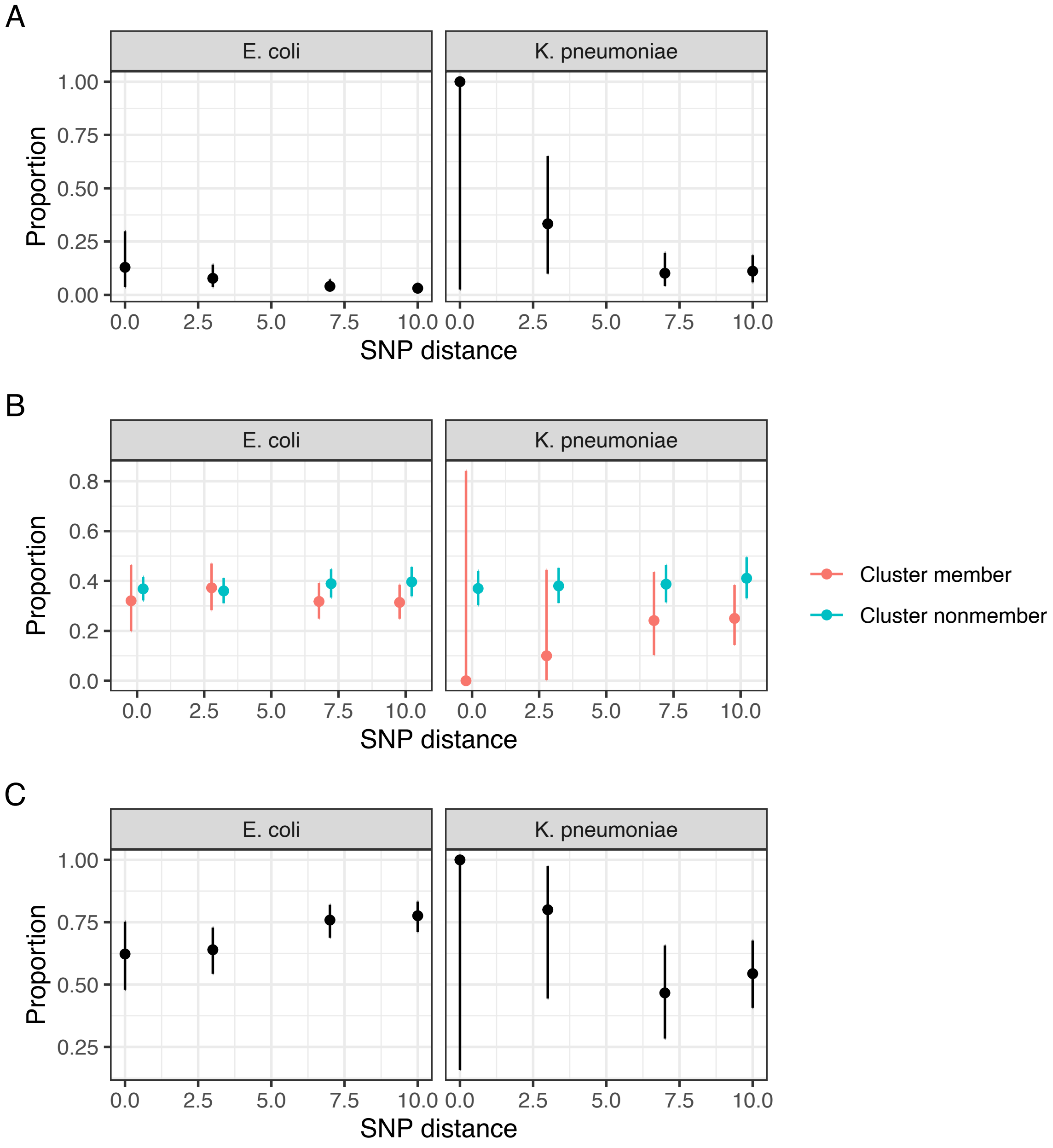


Supplementary Figure 26: Effect of varying SNP cluster definition on cluster epidemiology.
Sensitivity analysis examining epidemiology of SNP-clusters (i.e. putative transmission clusters) as the SNP-threshold varies from 0 to 10. (A) shows the proportion of pairwise comparisons of within-SNP-cluster isolates that are within a single participant; most putative transmission clusters are between-, rather than within- participants. (B) shows the proportion of isolates that are community associated stratified by whether they are members of a putative transmission cluster or not; the proportion is similar at all threshold values, which is not suggestive of hospital-associated transmission. (C) shows proportion of SNP clusters that contain two  or more hospital associated isolates. All confidence intervals are exact binomial confidence intervals.
