# Supplementary material for: Colonization dynamics of extended-spectrum beta-lactamase-producing Enterobacterales in the gut of Malawian adults
Source: Nat Microbiol. 2022 Sep 5;7(10):1593–604. doi: 10.1038/s41564-022-01216-7 (PMC9519460; doi:10.1038/s41564-022-01216-7)
Supplement: Supplementary file 2 [file 41564_2022_1216_MOESM2_ESM.pdf]

nature portfolio

Corresponding author(s): Joseph Lewis

Last updated by author(s): Jul 7, 2022

Reporting Summary

Nature Portfolio wishes to improve the reproducibility of the work that we publish. This form provides structure for consistency and transparency in reporting. For further information on Nature Portfolio policies, see our Editorial Policies and the Editorial Policy Checklist.

Statistics

For all statistical analyses, confirm that the following items are present in the figure legend, table legend, main text, or Methods section.

n/a	Confirmed	
☐	☒	The exact sample size (*n*) for each experimental group/condition, given as a discrete number and unit of measurement
☒	☐	A statement on whether measurements were taken from distinct samples or whether the same sample was measured repeatedly
☐	☒	The statistical test(s) used AND whether they are one- or two-sided<br>*Only common tests should be described solely by name; describe more complex techniques in the Methods section.*
☐	☒	A description of all covariates tested
☐	☒	A description of any assumptions or corrections, such as tests of normality and adjustment for multiple comparisons
☐	☒	A full description of the statistical parameters including central tendency (e.g. means) or other basic estimates (e.g. regression coefficient) AND variation (e.g. standard deviation) or associated estimates of uncertainty (e.g. confidence intervals)
☐	☒	For null hypothesis testing, the test statistic (e.g. F, t, r) with confidence intervals, effect sizes, degrees of freedom and P value noted<br>*Give P values as exact values whenever suitable.*
☐	☒	For Bayesian analysis, information on the choice of priors and Markov chain Monte Carlo settings
☐	☒	For hierarchical and complex designs, identification of the appropriate level for tests and full reporting of outcomes
☐	☒	Estimates of effect sizes (e.g. Cohen's d, Pearson's r), indicating how they were calculated

Our web collection on statistics for biologists contains articles on many of the points above.

Software and code

Policy information about availability of computer code

Data collection	ODK v1.1 (Get ODK inc. United States) and TeleForm v11.0 (Opentext, Canada).
Data analysis	Kraken v0.10.6, Bracken v1.0, SPAdes v3.14, Prokka v1.5, Roary v1.17, CheckM v1.1.3, mafft v7.205, snp-sites v2.4.1. IQ-TREE v1.6.3, ggtree v2.2.4, ARIBA v2.14.6, blastn v2.7.1, cd-hit-est v4.8.1, minimap2 v2.16, PopGenome v2.7.5 popPUNK v2.0.2, snippy v4.6.0, gubbins v3.0.0 , R v4.0.2, Stan v2.19, igraph v1.2.11, ggraph v2.0.5.<br><br>Analysis code to replicate the analysis is available at https://joelewis101.github.io/blantyreESBL/

For manuscripts utilizing custom algorithms or software that are central to the research but not yet described in published literature, software must be made available to editors and reviewers. We strongly encourage code deposition in a community repository (e.g. GitHub). See the Nature Portfolio guidelines for submitting code & software for further information.

Data

Policy information about availability of data

All manuscripts must include a data availability statement. This statement should provide the following information, where applicable:

- Accession codes, unique identifiers, or web links for publicly available datasets
- A description of any restrictions on data availability
- For clinical datasets or third party data, please ensure that the statement adheres to our policy

All data to reproduce this analysis are available as the blantyreESBL v1.2 R package available at https://joelewis101.github.io/blantyreESBL/, and on a mirrored Zendo repository (https://doi.org/10.5281/zenodo.5554081). Reads from all isolates sequenced as part of this study have been submitted to the European Nucleotide Archive under project IDs PRJEB26677, PRJEB28522 and PRJEB36486 and accession numbers linked to metadata are available in the R package as well as as supplementary data to this manuscript.

Human research participants

Policy information about studies involving human research participants and Sex and Gender in Research.

Reporting on sex and gender	Sex was based on self-reporting and is reported in Table 1.
Population characteristics	This information is provided in Table 1 of the manuscript
Recruitment	Adults (> 15 years) with sepsis, defined by fever and organ dysfunction criteria, were recruited from the emergency department of QECH 0700-1700 Monday to Friday as part of a study of sepsis aetiology, as described elsewhere33. Two comparator cohorts of participants were recruited: age and sex matched adults from QECH emergency department who had a plan from their attending clinical team to admit to hospital but no plan for antimicrobial administration; and community members matched by age, sex and home location to recruited sepsis patients. Exclusion criteria for the latter two groups were antimicrobial exposure within the past four weeks (except co-trimoxazole preventative therapy [CPT] and antituberculous chemotherapy); hospitalised participants who lacked capacity to give informed consent and had no guardian to give proxy consent; participants who spoke neither English nor Chichewa; and participants who lived > 30km from Blantyre city. Geographic matching on home location between community members and sepsis patients was achieved by random walk from the houses of sepsis participants with initial direction established by spinning a bottle on the floor. Written informed consent was obtained from all participants.<br><br>Bias from these recruitment criteria are unlikely - bias in ESBL carriage rates would need participants to differentially agree to recruitment depending on ESBL carriage status, which was unknown to them.
Ethics oversight	The study was approved by the research ethics committees of the Liverpool School of Tropical Medicine (16-062) and Malawi College of Medicine (P.11/16/2063)

Note that full information on the approval of the study protocol must also be provided in the manuscript.

Field-specific reporting

Please select the one below that is the best fit for your research. If you are not sure, read the appropriate sections before making your selection.

☒ Life sciences ☐ Behavioural & social sciences ☐ Ecological, evolutionary & environmental sciences

For a reference copy of the document with all sections, see nature.com/documents/nr-reporting-summary-flat.pdf

Life sciences study design

All studies must disclose on these points even when the disclosure is negative.

Sample size	The study described in this manuscript co-recruited 225 participants with sepsis recruited for a sepsis aetiolology study (published here: https://academic.oup.com/cid/advance-article/doi/10.1093/cid/ciab710/6354427). The study was powered to detect ESBL in stool at 28 days. Assuming 30% of participants with sepsis acquire ESBL-E by 28 days, and that 50% of them die by day 28, 100 antibiotic unexposed hospitalised and 100 community members (and antimicrobial unexposed) participants would give 80% power to detect a 50% relative difference in acquisition.
Data exclusions	No data were exuded form the analysis
Replication	Experimental replication is not applicable to this epidemiological/modelling study. However, all data and code have been made available to allow replication of our findings.
Randomization	This is a nonrandomised study, hence modeling techniques were used to account for differences between groups.

Blinding	This is a nonblinded study. Blinding is not relevant to an observational study such as this and it is not clear how or why it would be implemented.

Reporting for specific materials, systems and methods

We require information from authors about some types of materials, experimental systems and methods used in many studies. Here, indicate whether each material, system or method listed is relevant to your study. If you are not sure if a list item applies to your research, read the appropriate section before selecting a response.

Materials & experimental systems

n/a	Involved in the study
☒	☐ Antibodies
☒	☐ Eukaryotic cell lines
☒	☐ Palaeontology and archaeology
☒	☐ Animals and other organisms
☒	☐ Clinical data
☒	☐ Dual use research of concern

Methods

n/a	Involved in the study
☒	☐ ChIP-seq
☒	☐ Flow cytometry
☒	☐ MRI-based neuroimaging
